# *KIAA1109* Variants Are Associated with a Severe Disorder of Brain Development and Arthrogryposis

**DOI:** 10.1016/j.ajhg.2017.12.002

**Published:** 2017-12-28

**Authors:** Lucie Gueneau, Richard J. Fish, Hanan E. Shamseldin, Norine Voisin, Frédéric Tran Mau-Them, Egle Preiksaitiene, Glen R. Monroe, Angeline Lai, Audrey Putoux, Fabienne Allias, Qamariya Ambusaidi, Laima Ambrozaityte, Loreta Cimbalistienė, Julien Delafontaine, Nicolas Guex, Mais Hashem, Wesam Kurdi, Saumya Shekhar Jamuar, Lim J. Ying, Carine Bonnard, Tommaso Pippucci, Sylvain Pradervand, Bernd Roechert, Peter M. van Hasselt, Michaël Wiederkehr, Caroline F. Wright, DDD Study, Ioannis Xenarios, Gijs van Haaften, Charles Shaw-Smith, Erica M. Schindewolf, Marguerite Neerman-Arbez, Damien Sanlaville, Gaëtan Lesca, Laurent Guibaud, Bruno Reversade, Jamel Chelly, Vaidutis Kučinskas, Fowzan S. Alkuraya, Alexandre Reymond

**Affiliations:** 1 Center for Integrative Genomics, University of Lausanne, 1015 Lausanne, Switzerland; 2 Department of Genetic Medicine and Development, University of Geneva Medical School, 1211 Geneva, Switzerland; 3 Department of Genetics, King Faisal Specialist Hospital and Research Center, Riyadh 11211, Saudi Arabia; 4 Institut de Génétique et de Biologie Moléculaire et Cellulaire (IGBMC), CNRS UMR 7104, INSERM Unité 964, 67404 Illkirch Cedex, France; 5 Laboratoire de Diagnostic Génétique, Hôpitaux Universitaires de Strasbourg, 67000 Strasbourg, France; 6 Department of Human and Medical Genetics, Faculty of Medicine, Vilnius University, 08661 Vilnius, Lithuania; 7 Department of Genetics and Center for Molecular Medicine, University Medical Center Utrecht, 3584 CX Utrecht, the Netherlands; 8 KK Women’s and Children’s Hospital, Singapore 229899, Singapore; 9 Lee Kong Chian School of Medicine, Nanyang Technological University-Imperial College London, Singapore 639798, Singapore; 10 Service de Génétique, Hospices Civils de Lyon, 69002 Lyon, France; 11 Centre de Recherche en Neurosciences de Lyon, INSERM U1028, UMR CNRS 5292, Université Claude Bernard Lyon 1, 69675 Bron Cedex, France; 12 Département de Pathologie, Hospices Civils de Lyon, 69002 Lyon, France; 13 Department of Obstetrics and Gynecology, King Faisal Specialist Hospital and Research Center, Riyadh 11211, Saudi Arabia; 14 Swiss Institute of Bioinformatics (SIB), 1015 Lausanne, Switzerland; 15 Duke-NUS Medical School, Singapore 169857, Singapore; 16 Institute of Medical Biology, A^∗^STAR, Singapore 138648, Singapore; 17 Sant’Orsola-Malpighi Hospital, Medical Genetics Unit, Pavillon 11, 2nd floor, Via Massarenti 9, 40138 Bologna, Italy; 18 Deciphering Developmental Disorders (DDD) Study, Wellcome Trust Sanger Institute, Wellcome Trust Genome Campus, Hinxton, Cambridge CB10 1SA, UK; 19 Department of Clinical Genetics, Royal Devon and Exeter NHS Foundation Trust, Exeter EX1 2ED, UK; 20 Center for Fetal Diagnosis and Treatment, Children’s Hospital of Philadelphia, Philadelphia, PA 19104, USA; 21 Département d’imagerie pédiatrique et fœtale, Centre Pluridisciplinaire de Diagnostic Prénatal, Hôpital Femme Mère Enfant, Université Claude Bernard Lyon 1, 69677 Bron Cedex, France; 22 Institute of Molecular and Cell Biology (IMCB), A^∗^STAR (Agency for Science, Technology and Research), 61 Biopolis Drive, Singapore 138673, Singapore; 23 Department of Paediatrics, Yong Loo Lin School of Medicine, National University of Singapore, Singapore 119228, Singapore; 24 Amsterdam Reproduction & Development, Academic Medical Centre & VU University Medical Center, 1105 AZ Amsterdam, the Netherlands; 25 Department of Anatomy and Cell Biology, College of Medicine, Alfaisal University, Riyadh 11533, Saudi Arabia; 26 Saudi Human Genome Program, King Abdulaziz City for Science and Technology, Riyadh 12371, Saudi Arabia

**Keywords:** brain malformations, clubfoot, arthrogryposis, whole-exome sequencing, hydrocephaly, cerebellar hypoplasia

## Abstract

Whole-exome and targeted sequencing of 13 individuals from 10 unrelated families with overlapping clinical manifestations identified loss-of-function and missense variants in *KIAA1109* allowing delineation of an autosomal-recessive multi-system syndrome, which we suggest to name Alkuraya-Kučinskas syndrome (MIM 617822). Shared phenotypic features representing the cardinal characteristics of this syndrome combine brain atrophy with clubfoot and arthrogryposis. Affected individuals present with cerebral parenchymal underdevelopment, ranging from major cerebral parenchymal thinning with lissencephalic aspect to moderate parenchymal rarefaction, severe to mild ventriculomegaly, cerebellar hypoplasia with brainstem dysgenesis, and cardiac and ophthalmologic anomalies, such as microphthalmia and cataract. Severe loss-of-function cases were incompatible with life, whereas those individuals with milder missense variants presented with severe global developmental delay, syndactyly of 2^nd^ and 3^rd^ toes, and severe muscle hypotonia resulting in incapacity to stand without support. Consistent with a causative role for *KIAA1109* loss-of-function/hypomorphic variants in this syndrome, knockdowns of the zebrafish orthologous gene resulted in embryos with hydrocephaly and abnormally curved notochords and overall body shape, whereas published knockouts of the fruit fly and mouse orthologous genes resulted in lethality or severe neurological defects reminiscent of the probands’ features.

## Introduction

The advent of high-throughput sequencing led to the delineation of multiple syndromes. Neurological genetic diseases are the main class of these Mendelian disorders^1^ with, for example, approximately 700 different genes confidently associated with intellectual disability (ID) and developmental delay notwithstanding that about 50% of yet unexplained ID-affected case subjects are predicted to have a genetic basis in genes remaining to be discovered.^2^, ^3^ Neurodevelopmental disorders characterized by brain malformations represent an important group among these unexplained conditions and are likely associated with mutations in genes implicated in cortical or cerebellar development. They can be classified into four main categories depending on the origin of the defect.^4^ First are disorders due to abnormal proliferation of neuronal and glial cells including brain under-growth (microcephaly) or overgrowth (megalencephaly). Second are neuronal migration disorders that include: (1) lissencephaly, i.e., the absence or decrease of gyration responsible for a smooth brain; (2) cobblestone cortical malformations; and (3) neuronal heterotopia, i.e., the abnormal localization of a neuronal population. Third are pathologies characterized by malformations caused by postmigrational abnormal cortical organization, mainly polymicrogyria, i.e., the increase of small gyration. The last category regroups malformations of the mid-hindbrain with early anteroposterior and dorsoventral patterning defects. This phenotypic heterogeneity is paralleled by molecular heterogeneity as more than 100 genes have been implicated to date.^4^, ^5^, ^6^, ^7^ The causative genes can be arranged into specific biological pathways (for instance synapse structure, cellular growth regulation, apoptosis, cell-fate specification, actin cytoskeleton, and microtubule assembly) that do not necessarily correlate with the type of malformations described above, as emerging evidence suggests that brain disorders are far more heterogeneous than the classification suggests.^4^, ^8^

We report, through description of 19 affected individuals, an autosomal-recessive brain malformation disorder with arthrogryposis caused by variants within *KIAA1109* (MIM: 611565).

## Material and Methods

### Enrollment

Families were recruited in Lithuania, the United Kingdom, France, Saudi Arabia, the USA, and Singapore. The institutional review boards of the Vilnius University Faculty of Medicine, NHS Foundation Trust, Hôpitaux Universitaires de Strasbourg, King Faisal Specialist Hospital and Research Center, the Children’s Hospital of Philadelphia, “Hospices Civils de Lyon,” and KK Women’s and Children’s Hospital approved this study. Participants were enrolled after written informed consent was obtained from parents or legal guardians. The clinical evaluation included medical history interviews, a physical examination, medical imaging as appropriate, and review of medical records.

### Exome Sequencing and Analysis

To uncover genetic variants associated with the phenotypes of the two affected members of the Lithuanian (LT) family, we sequenced their exomes and that of their parents, as described.^9^ DNA libraries were prepared from leukocytes by standard procedures. Exomes were captured and sequenced using different platforms as specified below to reach 50- to 120-fold coverage on average. Variants were filtered based on inheritance patterns including autosomal recessive, X-linked, and *de novo*/autosomal dominant. Variants with MAF < 0.05% in control cohorts (dbSNP, the 1000 Genome Project, NHLBI GO Exome Sequencing Project, the ExAC, and our in-house databases) and predicted to be deleterious by SIFT,^10^ PolyPhen-2,^11^ and/or UMD predictor^12^ were prioritized. This exome analysis singled out compound heterozygote variants in *KIAA1109* as possibly causative in both affected siblings, prompting us to look for other individuals with overlapping phenotypes and variants in the same gene through GeneMatcher, the DDD portal, and clinical genetics meetings. These searches led to the identification of a total of 17 additional affected individuals.

In the Algerian (AL) family, exome sequencing was performed by the Centre National de Génotypage (CNG, Evry, France), Institut de Génomique, CEA. Exomes were captured with Human All Exon v5; 50 Mb (Agilent Technologies) and sequenced on a HiSeq2500 platform (Illumina) as paired-end 100 bp reads. For the Saudi Arabian (SA1–SA3) families, exome capture and sequencing was performed in conjunction with autozygome analysis as previously described.^13^ For the family from Singapore (SG), exome capture, sequencing, and variant calling and analysis were performed as described.^14^ For the two families from Tunisia (TU1 and TU2), exome sequencing was performed on a NextSeq500 (Illumina) after SeqCapEZ MedExome Library preparation and analyzed with BWA and GATK HaplotypeCaller. Variants with MAF < 0.1% in ExAC database and predicted to be deleterious by SIFT,^10^ PolyPhen-2,^11^ and Mutation Taster^15^ were prioritized. The UK family’s exome capture and sequencing was performed as previously described.^16^ For the US family, exome capture, sequencing, and variant calling and analysis were performed as described.^16^, ^17^ The breakpoints of the paternally inherited deletion were determined by whole-genome sequencing.

### Breakpoint Mapping by Whole-Genome Sequencing

100 ng of genomic DNA were sheared using Covaris with a target fragment size of 500 bp. The sequencing library was prepared using Tru-Seq DNA PCR-free Sample Prep Kit (Illumina) and 100-bp paired-end reads sequenced on a HiSeq 2500 platform (Illumina). The PCR-free kit was used to prepare the library in order to avoid PCR duplicates. Sequence-control, software real-time analysis, and bcl2fastq conversion software v.1.8.4 (Illumina) were used for image analysis, base calling, and demultiplexing. Purity-filtered reads were adapters- and quality-trimmed with FastqM*cf.* v.1.1.2 and aligned to the human_g1k_v37_decoy genome using BWA-MEM (v.0.7.10^18^). PCR duplicates were marked using Picard tools (v.2.2.1). We obtained a sequence yield of 11.4 Gb of aligned bases with a 3.6× mean coverage. Aligned reads within the *KIAA1109* locus were visualized and evaluated using Integrative Genomics Viewer (IGV) in search of chimeric inserts. We identified a single pair of paired-ends reads mapping unequivocally 8,971 bp apart within *KIAA1109* allowing us to map the paternally inherited deletion of the US proband breakpoints within exon 68 and intron 72. The breakpoints were then finely mapped with Sanger sequencing to coordinates chr4:123254885 and chr4:123263438 (hg19) (Figure S1).

### Zebrafish Manipulations, CRISPR/Cas9 Editing, and Design of Morpholinos

Zebrafish animal experimentation was approved by the Ethical Committee for Animal Experimentation of the Geneva University Medical School and the Canton of Geneva Animal Experimentation Veterinary authority. Wild-type TU (Tübingen) zebrafish were maintained in standard conditions (26°C–28°C, water conductivity at 500 μS [pH 7.5]). Embryos obtained by natural matings were staged according to morphology/age.

Zebrafish *kiaa1109* mutant lines were developed using CRISPR-Cas9-mediated genome editing. Using the ZiFiT online tool,^19^ we identified three suitable 20-nucleotide sites upstream of protospacer adjacent motifs (PAM) for *S. pyogenes* Cas9 and targeting *kiaa1109* exons 1, 4, and 7 (numbering according to GenBank: NM_001145584.1). Annealed oligonucleotides carrying the 20-nucleotide target sequence were ligated into pDR274 (Addgene plasmid # 42250), and clones verified by Sanger sequencing, linearized, and used for *in vitro* transcription of single-guide RNAs (sgRNAs) using the MEGAshortscript T7 Transcription Kit (ThermoFisher). sgRNAs were mixed with recombinant Cas9 nuclease (PNA Bio), Danieau buffer, and phenol red as a tracer, and approximately 1 nL injected into early zebrafish embryos. Each injection contained 0.25 ng of sgRNA and 0.5 ng of Cas9 per nL. Evidence for genome editing was assessed qualitatively by PCR amplification around the target sites in each exon in injected embryo lysates. Heterogeneous PCR products, consistent with mosaic editing, was seen as smeared bands by gel electrophoresis, compared to uninjected embryos (not shown). Injected fish embryos were raised to adulthood and screened for their ability to transmit mutant *kiaa1109* alleles by out-crossing and PCR genotyping. PCR products were cloned with pCRII TOPO (ThermoFisher) to separate alleles, and colony PCRs were sequenced to detect germline transmission of potential *kiaa1109* frameshift alleles. Out-crossed F1 embryos were raised to adulthood for mutations detected in exon 1, 4, and 7, as separate lines. F1 adult fish were tail-clipped, targeted exons were amplified by PCR, and PCR products were cloned to pCRII TOPO (ThermoFisher) to identify specific *kiaa1109* mutant alleles in heterozygosity by colony PCR and DNA sequencing. Heterozygous F1 fishes carrying the same *kiaa1109* mutation were then in-crossed to assess embryonic survival and phenotype in homozygosity. *kiaa1109* genotyping for embryos from these crosses was made using PCR, amplifying the target exon regions. Products from wild-type, heterozygous, or homozygous mutant amplicons were distinguished by gel electrophoresis. Details of the sgRNA target sites, representative mutant allele sequencing chromatograms, and predicted frameshifts for the three *kiaa1109* mutant lines described are given in Figures S2 and S3.

To knock down *kiaa1109* (GenBank: NM_001145584.1) in zebrafish, we designed two non-overlapping splice-blocking MOs (morpholinos) targeting pre-mRNA: (1) sbE4MO- 5′-TGTTCTGTTTTTGCACTGACCATGT-3′ and (2) sbE2MO- 5′-CAACATTGAGACAGACTCACCGATG-3′ (Gene Tools) that target the exon 4/intron 4 and exon 2/intron 2 boundaries, respectively. The standard Ctrl-MO (5′-CCTCTTACCTCAGTTACAATTTATA-3′) (Gene Tools) without any targets in the zebrafish genome was used for mock injections. MOs were dissolved in nuclease-free water and their concentrations determined by NanoDrop. The fish were injected at 1- to 2-cell stages (1–2 nL) using phenol red as a tracer in Danieau buffer. The following amounts of MO: 3.35 and 6.7 ng of sbE4MO; 5.6, 11.3, 16.9, and 22.3 ng of sbE2MO and the equivalent of Ctrl-MO for the higher doses were injected into wild-type zebrafish embryos, respectively. Uninjected, standard control MO, and *kiaa1109* MO-injected embryos were collected at 2 dpf and total RNAs were isolated using standard Trizol protocol (Invitrogen). 1 μg of total RNA from each sample was used to synthesize cDNA with the Superscript III kit with Oligo d(T) primers (Invitrogen). Dilutions 1/20 of cDNA were used for standard PCR reactions (JumpStart RED Taq ReadyMix, Sigma-Aldrich). Basic quantifications of agarose gels were performed with ImageQuant TL software (GE Healthcare). We assessed embryos for morphological changes at 2 days post-fertilization. We grouped the embryos into four classes by morphology: normal embryos, embryos with clear midbrain and/or hindbrain ventricle swelling, curved embryos, and embryos with both phenotypes. The degree of hydrocephaly was not measured; hydrocephaly was assessed by clear deviation from the normal embryo morphology (see Results). Curved embryos showed caudal axis curvature. They were clearly distinguishable from the straight anterior-posterior axis of normal 2-day-old embryos (see Results). The most severely affected embryos had a combination of hydrocephaly and caudal axis curvature (see Results).

## Results

**Figure 1:**
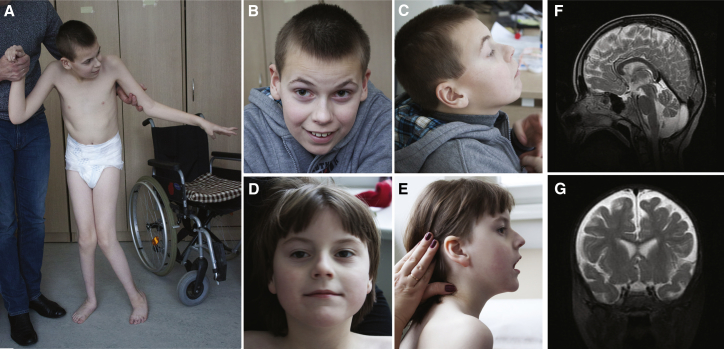
Pictures and Brain MRI from Surviving Individuals Front and side views of the LT affected brother LT.II.1 (A–C) and sister LT.II.2 (D, E) at the ages of 13 years and 7 years, respectively. Brain MRI images of affected individual LT.II.1 at age of 8 years showed small posterior fossa arachnoid cyst, discrete vermian atrophy, and slight increase of the fluid-filled retro and infra-cerebellar space (F). Brain MRI images of affected individual LT.II.2 at age of 1 year showed discrete parenchymal rarefaction involving mainly the frontal lobes (G).

We first identified compound heterozygous missense variants in *KIAA1109* in a Lithuanian family with two affected siblings (LT.II.1 and LT.II.2) presenting with a constellation of severe global developmental delay, cerebral parenchymal rarefaction and ventriculomegaly (observed at 20 months of age), plagiocephaly, paretic position of hands and feet at birth, early-onset epilepsy, muscle hypotonia, stereotypical movements, hypermetropia, and lack of walking function (Table 1, Figure 1). As a homozygous stop-gain allele in this gene was suspected to cause a syndromic neurological disorder in a fetus (described in more details in this manuscript as fetus SA1.II.1) with cerebellar malformations, hydrocephalus, micrognathia, club feet, arthrogryposis with flexed deformity, pleural effusion, and death 1 hr after birth,^13^ we hypothesized that *KIAA1109* variants cause an autosomal-recessive (AR) brain development disorder with arthrogryposis.

**Table 1:** Overlapping Clinical Features of Individuals with KIAA1109 Variants

**Family #**	**Individual #**	**Gender, Age**	**Ethnicity**	**Gene Mutations**	**ID**	**Mutation Coordinates (GRCh37/hg19)**	**Cerebral Anomalies (Pre/Post-natal Images) and Pathological Findings**	**Head and Face**	**Eyes**	**Mouth**	**Joints**	**Limbs**	**Gastro-intestinal**	**Urogenital**	**Heart**	**Muscles**	**Behavior**	**Other Symptoms**
LT	LT.II.1 (brother)	male, 13 yo	Lithuanian	compound heterozygote	severe, global developmental delay, no language, cannot stand or walk without support	Chr4:123160823; c.3986A>C, Chr4:123170727; c.5599G>A	post-natal brain MRI: small posterior fossa arachnoid cyst, discrete vermian atrophy, slight increase of the fluid-filled retro and infra-cerebellar space and mild enlargement of subarachnoid spaces of frontal regions.	plagiocephaly	hypermetropia, strabismus, astigmatism	delayed eruption of permanent teeth	mild contractures of large joints	syndactyly of 2nd and 3rd toes, hands and feet paresis at birth, talipes valgus	normal	scrotum hypoplasia	none	muscle hypotonia, atrophy	stereotypic movements, spontaneous paroxysms of laughter	early-onset epilepsy
LT	LT.II.2 (sister)	female, 7 yo	Lithuanian	compound heterozygote	severe, global developmental delay, no language, cannot sit or stand without support	Chr4:123160823; c.3986A>C, Chr4:123170727; c.5599G>A	post-natal brain MRI: discrete parenchymal rarefaction involving the frontal lobes	plagiocephaly	hypermetropia, strabismus, astigmatism	normal	mild contractures of large joints	paretic position of hands and feet in infancy, talipes valgus	chronic constipation	none	none	muscle hypotonia, atrophy	stereotypic movements	early onset epilepsy, dermatitis, psoriasis
UK	UK.II.1, DDD# 263241	female, 11 yo	British	compound heterozygote with one *de novo* missense mutation	global developmental delay, mild to moderate learning disability	Chr4:123164200; c.4719G>A and Chr4:123171679; c.5873G>A	prenatal imaging (US and MRI): major microcephaly (HC -5 SD) with reduced white matter volume and mild ventriculomegaly	hypertelorism, slightly upslanting palpebral fissures	ocular motor apraxia, hypermetropia, strabismus	dental crowding, high palate	mild bilateral talipes managed by physiotherapy only; asymmetry of the thorax	syndactyly of 2nd and 3rd toes, 5th toe clinodacytly, hallux valgus	gastro-esophageal reflux	none	complex congenital heart disease (tetralogy of Fallot with pulmonary atresia)	none	poor concentration, immature behavior with minor self-harm (head-banging) when angry/frustrated	none
AL	AL.II.1	male, termination of pregnancy at 21 weeks of amenorrhea	Algerian	homozygous missense mutation	not applicable	Chr4:123207807; c.9149C>A	prenatal US findings: triventricular ventriculomegaly and corpus callosum agenesis; neuropathological findings: absence of cortical lamination and diffuse migration anomalies within a thin parenchymal mantle, ventriculomegaly, and voluminous germinal matrix. Corpus callosum was not identified. Infra-tentorial space: hypoplasia of the pons with absence of the longitudinal and transversal fibers and dysplasia of the cerebellum characterized by lack of foliation and poorly identified vermis; narrowing of the aqueduct	hypertelorism, posteriorly rotated ears	bilateral cataract with crystalline fibers of variable size and orientation	retrognathism, big horizontalized mouth	arthrogryposis (flexed deformity of shoulders, elbow and hips, and bilateral adductus thumbs)	bilateral equinovarus foot	choanal atresia	scrotum hypoplasia	pericardial effusion	not available	not applicable	slight pleural effusion, peritoneal effusion, dilatation of lymph vessels in lung with lympho-hematopoietic elements
TU1	TU1.II.1	female, died at 3 days of age	Tunisian	homozygous missense mutation	not applicable	Chr4:123230520; c.10153G>C	prenatal imaging (US and MRI): cerebellar hypoplasia and brainstem dysgenesis (flat and elongated pons and slightly kinked brainstem with increased fluid filled retro-cerebellar spaces); severe parenchymal thinning with major lack of gyration (lissencephalic aspect) associated with voluminous germinal matrix protruding within moderate ventriculomegaly and absence of corpus callosum. Cephalic biometry was normal.	hypotelorism	none	deep palate	left club foot	long fingers	none	none	left heart hypoplasia	not available	not applicable	none
TU1	TU1.II.4	male, termination of pregnancy at 23 weeks	Tunisian	homozygous missense mutation	not applicable	Chr4:123230520; c.10153G>C	prenatal US findings: severe parenchymal thinning with lack of gyration associated with ventriculomegaly and corpus callosum agenesis. Neuropathological findings: complete corpus callosum agenesis, ventricular dilatation, severe cortical malformations with a reduced cortical plate, neuronal depletion, heterotopia within white matter, dysplasia of brainstem and cerebellum.	none	none	none	arthrogryposis (hip and shoulder contractures)	clenched hands, camptodactyly, club feet	none	none	none	not applicable	not applicable	none
TU2	TU2.II.2	female, died at 12 days of age	Tunisian	homozygous missense mutation	not applicable	Chr4:123230520; c.10153G>C	prenatal imaging (US and MRI): cerebellar hypoplasia and dysgenesis associated to severe brainstem dysgenesis characterized by flat and elongated pons and slightly kinked brainstem with increased fluid filled retro-cerebellar spaces. Corpus callosum was not identified. Supratentorial anomalies include severe parenchymal thinning associated with lissencephalic aspect as well as voluminous germinal matrix protruding within severe ventriculomegaly.	none	microphthalmia, blepharophimosis	none	club feet	club feet and hands	none	none	none	hypotonia	not applicable	narrow chest
SA1	SA1.II.1, 13DG1900	female, death at 1 hr after delivery	Saudi	homozygous nonsense mutation	not applicable	Chr4: 123128323; c.1557T>A	prenatal US findings: severe ventriculomegaly with supratentorial cerebral mantle thinning associated with cerebellar hypoplasia	small eyes, low-set ears	small eyes	micrognathia	severe arthrogryposis (fixed elbows, fixed bilateral talipes, bilateral overlapping fingers, bilateral clinodactyly)	bilateral club foot	not available	not available	not available	not available	not applicable	pleural effusion
SA2	SA2.II.1, 15DG0595	female, stillborn	Saudi	homozygous splice mutation	not applicable	Chr4:123252480; c.11250−1G>A	prenatal US findings: hydrocephalus, absent corpus callosum, hypoplastic cerebellum	not available	not available	not available	arthrogryposis multiplex	bilateral overlapping fingers, bilateral cleft feet, bilateral cleft toes, and bilateral sandal gaps	normal	bilaterally abnormal kidneys	not available	not available	not applicable	skeletal shortening, nuchal thickening
SA3	SA3.II.1, 15DG1933	female, stillborn	Saudi	homozygous nonsense mutation	not applicable	Chr4:123258092; c.12067G>T	prenatal US findings: hydrocephalus, hypoplastic cerebellum	not available	not available	not available	arthrogryposis	bilateral talipes	normal	normal	absent fetal heart	not available	not applicable	skin edema
US	US.II.3	male, termination of pregnancy at 19 weeks	Caucasian	compound heterozygote	not applicable	Chr4:123113479; c.997dupA and Chr4: 123254885_123263438del; c.11567_12352delinsG	prenatal imaging (US and MRI): severe ventriculomegaly, thin cerebral parenchyma and cortical mantle associated with lissencephalic pattern, prominent germinal matrix, brain stem and vermian dysgenesis (kinked brain stem) and elongated pons; corpus callosum agenesis	low-set ears, webbed neck	normal	unremarkable	severe arthrogryposis with flexion contractures and pterygia, hyperflexed wrists, bilateral clinodactyly	bilateral talipes	normal	echogenic malrotated bowel without ascites, short penis with bulbous shaft	coarctation of the aorta	muscle atrophy	not applicable	low conus non-immune hydrops with scalp edema, cystic hygroma, anal atresia, bilateral pleural effusion
SG	SG.II.1	male, died at 3 months of age	Chinese	compound heterozygote	not applicable	Chr4:123147970; c.2902C>T and Chr4:123159280; c.3611delA	post-natal MRI: supratentorial findings include both severe parenchymal (or cerebral mantle) thinning and smooth cortical surface, germinolytic cysts involving voluminous germinal matrix protruding within severe ventriculomegaly without any identification of corpus callosum. Infratentorial findings include severe cerebellar hypoplasia with severe brain-stem dysgenesis characterized by a kinking aspect.	macrocephaly; hypertelorism; posteriorly rotated ears; flattened nasal bridge	congenital cataract; microphthalmia	no structural anomalies	arthrogryposis (involving bilateral shoulders, elbows, wrists, hands, knees)	bilateral structural congenital talipes equinovarus (CTEV)	ano-rectal malformation with recto-perianal fistula	no structural anomalies	small atrial septal defect/patent foramen ovale	hypotonia	not applicable	excess skin folds of neck
SG	SG.II.4	male, died at 1 month of age	Chinese	compound heterozygote	not applicable	Chr4:123147970; c.2902C>T and Chr4:123159280; c.3611delA	post-natal MRI: supratentorial findings include both severe parenchymal (or cerebral mantle) thinning and smooth cortical surface, germinolytic cysts involving voluminous germinal matrix protruding within severe ventriculomegaly without any identification of corpus callosum. Infratentorial findings include severe cerebellar hypoplasia with severe brain-stem dysgenesis characterized by a kinking aspect	macrocephaly; hypertelorism; bilateral low-set ears, short nose; anteverted nares	congenital cataracts; microphthalmia	no structural anomalies	arthrogryposis (involving bilateral elbows, wrists, hands, knees, hips)	bilateral structural congenital talipes equinovarus (CTEV)	normal	no structural anomalies	small to moderate fenestrated atrial septal defect	hypotonia	not applicable	webbed neck; inverted nipples

**Figure 2:**
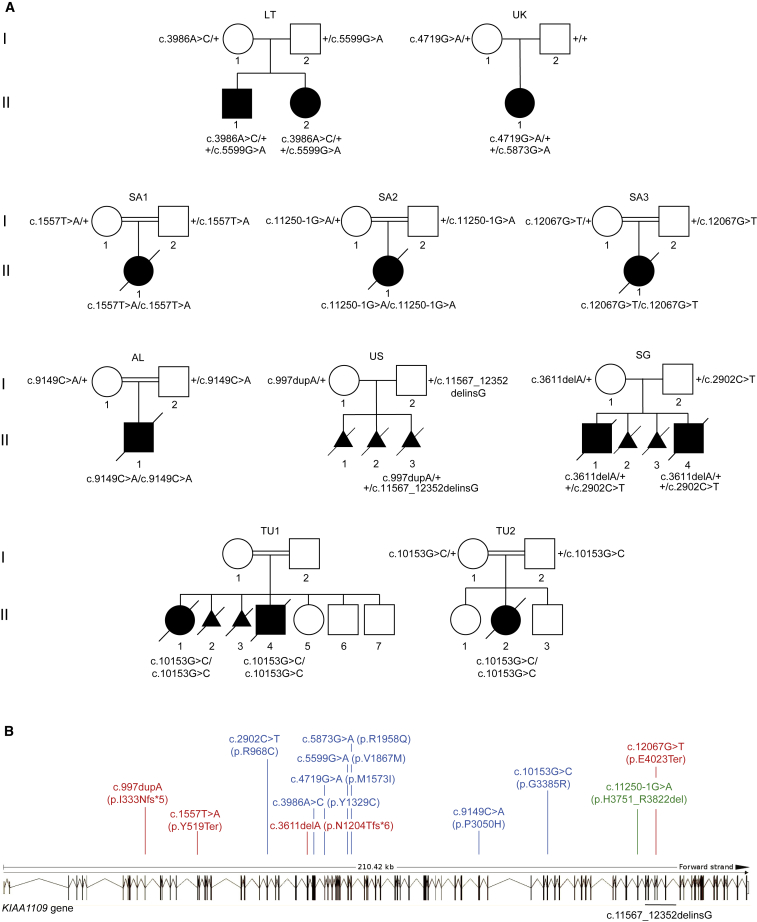
*KIAA1109* Pedigrees and Variants (A) Pedigrees of the ten families carrying *KIAA1109* variants. The affected individuals of the Lithuanian (LT), Singaporean (SG), British (UK), and American (US) families are compound heterozygotes for rare variants, whereas the probands of the Algerian (AL), Saudi Arabian (SA1–SA3), and Tunisian (TU1, TU2) consanguineous families are homozygous for *KIAA1109* variants. (B) Distribution of variants along the schematically represented 86 exons of *KIAA1109*. Missense variants are depicted in blue, nonsense in red, and the splice site variant in green. The extent of the deletion identified in the proband of the US family is indicated in black below.

Our searches for more case subjects led to the identification of a total of 19 affected individuals from 10 families (including 6 undiagnosed miscarriages) recruited in Algeria (AL), Lithuania (LT), Saudi Arabia (SA1–SA3), Singapore (SG), Tunisia (TU1, TU2), the United Kingdom (UK), and the United States of America (US) (Figure 2A). Genetic variants associated with the complex phenotype of interest were uncovered through exome sequencing of the affected individuals and their healthy parents with the exception of SG.II.4. We found only one gene, *KIAA1109*, compliant with AR Mendelian expectations and bearing two putatively deleterious variants in all affected individuals. GENCODE^20^ catalogs in Ensembl 16 isoforms of *KIAA1109*; two encode the full-length 5,005-amino acids protein, six have no coding potentials, and the remaining eight isoforms encode protein of lengths varying from 164 to 1,674 amino acids. All the mutations reported in this manuscript affect the full-length GenBank: NP_056127 protein. Consistent with consanguineous unions, the affected members of families AL, TU1/TU2, SA1, SA2, and SA3 were homozygous for variants c.9149C>A (p.Pro3050His), for c.10153G>C (p.Gly3385Arg), for c.1557T>A (p.Tyr519Ter), for c.11250−1G>A (r.11250_11465del, p.His3751_Arg3822del), and for c.12067G>T (p.Glu4023Ter), respectively, whereas the affected individuals from LT, SG, UK, and US families were heterozygote for c.3986A>G (p.Tyr1329Cys) and c.5599G>A (p.Val1867Met), for c.2902C>T (p.Arg968Cys) and c.3611delA (p.Asn1204Thrfs^∗^6), for c.4719G>A (p.Met1573Ile) and the *de novo* c.5873G>A (p.Arg1958Gln), and for c.997dupA (p.Ile333Asnfs^∗^5) and the deletion g.123254885_123263438delinsG (c.11567_12352delinsG, p.Lys3856Argfs^∗^44), respectively (nomenclature according to GenBank: NM_015312.3, NP_056127.2; Figures 2A and 2B, Table S1). The fact that families TU1 and TU2 are not known to be related suggests a Tunisian founder effect of variant c.10153G>C (p.Gly3385Arg). Sanger sequencing in each family confirmed the anticipated segregation of the *KIAA1109* variants, with the exception of family TU1 whose parents declined to be assessed. It also confirmed the genetic status of the SG.II.4 affected sibling (Figure 2A). All variants are either absent or encountered (as heterozygous variants) with a frequency lower than 1/10,000 in ExAC (v.0.3.1)^21^ (Table S1). The missense variants are predicted to be functionally damaging at least by two of the three PolyPhen-2,^11^ Provean,^22^ and SIFT^10^ predictors with the exception of the UK.II.1 variants predicted to be benign, neutral, and tolerated, respectively (Table S1). They might be under “compensated pathogenic deviation in human, a phenomenon that contributes to an unknown, but potentially large, number of false negatives to the evaluation of functional sites” as demonstrated in Jordan et al.^23^ Missense variants and CNVs are underrepresented compared to expectation in ExAC (missense Z score = 4.97; CNV Z score = 0.77) indicating that *KIAA1109* is under constraint. The identification of 50 LoF variants compared to the 176.1 expected, while not significant with a pLI = 0.0, does not contradict this hypothesis. In agreement with a possible contributing role of bi-allelic *KIAA1109* LoF variants to the phenotype of affected individuals SA1.II.1, SA2.II.1, SA3.II.1, and US.II.3, ExAC does not report homozygous LoF variants in *KIAA1109*. The splice variant c.11250−1G>A identified in fetus SA2.II.1 is predicted to abolish the consensus acceptor site of intron 66.^24^ A prediction validated by our RT-PCR experiments that showed a partial skipping of 216-nucleotides-long exon 67 in lymphoblastoid cell line of the affected SA2.II.1 fetus (Figure S4). The corresponding transcript would encode a protein lacking 72 amino acids. All missense variants identified in the AL, LT, SG, TU, and UK families affect highly conserved residues within evolutionary conserved region of the encoded protein (Figure S5).

**Figure 3:**
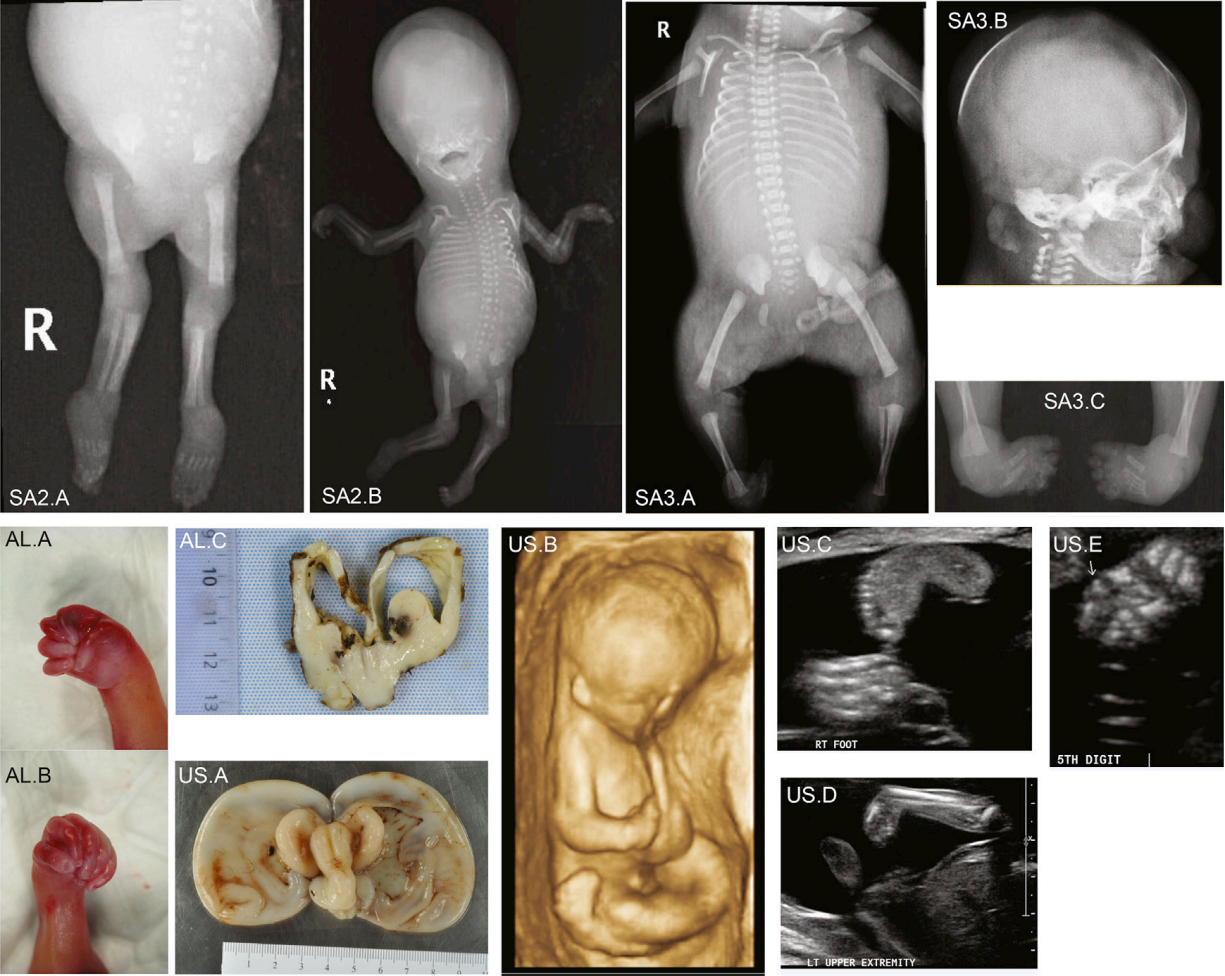
Ultrasound, X-Rays, and Autopsy Images of the SA2.II.1, SA3.II.1, AL.II.1, and US.II.3 Fetuses X-ray images showing arthrogryposis of SA2.II.1 fetus (SA2.A and SA2.B). X-ray images showing SA3.II.1 skeleton (SA3.A), head (SA3.B), and club feet (SA3.C). Autopsy pictures from the AL.II.1 fetus showing right (AL.A) and left (AL.B) adductus thumbs of the fetus, and dilatation of cerebral ventricles with agenesis of corpus callosum (AL.C). Autopsy image of the brain from US.II.3 fetus showing hydrocephalic brain with diaphanous pallium (US.A). The colliculi appear as single elongated ridges separated by a midline futter and the midline appears angulated on the brainstem, which is small as is the cerebellum. Antenatal ultrasound scan showed general arthrogryposis (US.B), one hyperflexed wrist (US.C), club feet (US.D), and bilateral clinodactyly of one hand (US.E).

**Figure 4:**
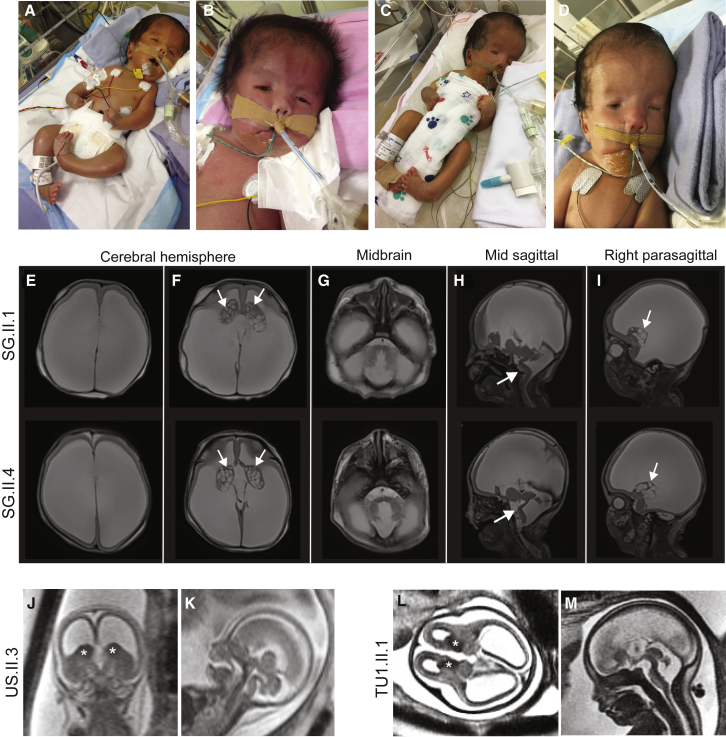
Pictures and Brain MRI Images of the SG.II.1 and SG.II.4 Babies and US.II.3 and TU1.II.1 Fetuses (A–D) Photographs of SG.II.1 (A and B) and SG.II.4 (C and D) babies showing their whole bodies (A and C) and a close up of their faces (B and D). (E–I) Brain MRI images of the elder brother SG.II.1 (top) and the younger brother SG.II.4 (bottom). Axial T2 weighted images showed severe ventriculomegaly, associated with severe thinning of the brain parenchyma (E, F). The brain parenchyma showed absence of normal gyral/sulcal pattern with smooth appearance in keeping with lissencephaly (E, F). Corpus callosum appeared to be absent (E, H). Note the prominent germinal matrix with germinolysis cysts (solid arrows) (F, I). The pons and cerebellum appeared hypoplastic with dilatation of the 4^th^ ventricle (G, H) and Z shaped appearance of the brainstem (solid arrows) (H). (J–M) Coronal (J), axial (L), and midsagittal (K, M) T2-weighed fetal prenatal MRI images of US.II.3 at 18.5 weeks of pregnancy (J and K) and TU1.II.1 at 28 weeks of pregnancy (L and M) demonstrating a similar imaging pattern including thin parenchyma (lissencephalic aspect), prominent germinal matrix marked by an asterisk, ventriculomegaly, and brain stem and vermian dysgenesis (kinked brain stem and elongated pons). In summary, we observe a similar brain malformation pattern both prenatally—US.II.3 in (J) and (K), TU1.II.1 in (L) and (M), AL.II.1 (see text), TU1.II.4 (see text), and TU2.II.2 (see text)—and postnatally (SG.II.1 [E–I top] and SG.II.4 [E–I bottom]).

As exemplified by the LT.II.1 and LT.II.2 siblings and the SA1.II.1 proband, the phenotype of the 19 affected individuals ranges from global developmental delay with/without inability to stand to stillbirth. Many of the more severely affected case subjects harbor homozygous or compound heterozygote truncating alleles (families SA1–SA3 and US) (Table 1, Supplemental Note). While the phenotype of proband SA1.II.1 is summarized above, SA2.II.1 and SA3.II.1 stillborn fetuses shared hydrocephalus, cerebellar hypoplasia, arthrogryposis, and skeletal anomalies (Figures 3 and S6). Proband SA2.II.1 had bilateral overlapping fingers, apparent contractures of the hands and feet, and bilateral sandal gaps, along with shortened long bones and nuchal thickening. He also presented with absence of corpus callosum and abnormal kidneys. Proband SA3.II.1 showed skin edema, bilateral talipes, and arthrogryposis (Figure 3; Table 1; Supplemental Note). The US.II.3 stillborn fetus resulted from a 3^rd^ pregnancy attempt of the couple (Figure 2A). The fetus demonstrated major central nervous anomalies including thin cerebral parenchyma with lissencephalic pattern, prominent germinal matrix, ventriculomegaly, brain stem vermian dysgenesis (kinked brain stem and elongated pons), and absence of corpus callosum, as well as closed spinal defect at L4-L5, associated with extra-central nervous anomalies including coarctation of the aorta, small omphalocele, echogenic bowel, hydrops, cystic hygroma, pleural effusion, possible anal atresia, low-set ears, short penis, clinodactyly, talipes, and abnormal posturing of the limbs (Figure 3). The SG family had four pregnancy attempts; two resulted in miscarriages (SG.II.2 and SG.II.3) and two in fetuses who did not pass the first semester (Figures 2A and 4). The SG.II.1 elder brother had minimal respiratory effort at birth and required immediate intubation and mechanical ventilation. He presented with macrocephaly, hypertelorism, posteriorly rotated ears, flattened nasal bridge, congenital cataract, and microphthalmia. He had generalized arthrogryposis and bilateral congenital talipes equinovarus. He also had hypotonia and an ano-rectal malformation with recto-perianal fistula (Figure 4). Brain MRI showed major cerebral parenchymal thinning with lissencephalic aspect, severe ventriculomegaly, absence of corpus callosum, and severe cerebellar and pontine hypoplasia (Figure 4). He passed away at 3 months of age from pneumonia and septic shock. The SG.II.4 younger sibling was remarkably similar to his elder with hypertelorism, bilateral low-set ears, short nose, anteverted nares, bilateral congenital cataract, microphthalmia, webbed neck, bilateral structural congenital talipes equinovarus, generalized arthrogryposis, and hypotonia. Brain MRI showed severe hydrocephalus with marked thinning of the cerebral parenchyma. The corpus callosum was absent, the cerebellum and brainstem were hypoplastic, and there was a pontomesencephalic kink. He remained ventilator dependent from birth and passed away at 1 month of age (Figure 4). The AL.II.1 fetus presented with an equally severe phenotype so the parents elected to terminate the pregnancy. He showed multiple brain malformations including hydrocephalus, vermis fusion, lamination defect of cerebellar cortex, and absence of the corpus callosum, combined with arthrogryposis with flexed deformity and bilateral adductus thumbs, diffuse effusion, and other clinical features (Figures 3 and S6; Table 1; Supplemental Note). Affected individuals and fetuses TU1.II.1, TU1.II.4, and TU2.II.2 had arthrogryposis and the same cerebral malformative pattern, associating cerebellar and brainstem dysgenesis, parenchymal thinning with major lack of gyration, corpus callosum agenesis, and hyperplastic germinal matrix protruding within ventriculomegaly. The severity of features of the AL.II.1 fetus, the SG.II.1 and SG.II.4 siblings, and the TU1.II.1, TU1.II.4, and TU2.II.2 Tunisian affected individuals and their resemblance with those observed in carriers of truncating variants suggest that the missense variants p.Pro3050His, p.Gly3385Arg, and p.Arg968Cys act as LoF or strong hypomorphs. Consistent with this hypothesis, the C>T transition in exon 24 of the latter variant is predicted to alter an exonic splicing enhancer site and thus proper splicing. More experiments are warranted to further demonstrate these assumptions.

All case subjects compatible with life carry missense variants (Figure 2; Table S1; Supplemental Note). Whereas the two LT.II.1 and LT.II.2 Lithuanian siblings are briefly described above, the UK.II.1 British proband showed global developmental delay, microcephaly, absence of the pulmonary valve, tetralogy of Fallot and ventricular septal defect, ocular motor apraxia, hypermetropia, dental crowding, 5^th^ toe clinodactyly, syndactyly of the 2^nd^ and 3^rd^ toe like the LT.II.1 elder sibling, hallux valgus, and pes planus (Supplemental Note).

In line with the clinical presentation of LoF affected individuals, ablation in fruit flies and mice of the *KIAA1109* orthologs, *tweek* and *Kiaa1109*, respectively, resulted in lethality. Whereas *Kiaa1109*^*−*/−^ mice engineered and phenotyped by the International Mouse Phenotyping Consortium^25^, ^26^ exhibited complete penetrance of pre-weaning lethality, some rare homozygous *tweek* mutants survive to adulthood.^27^ These survivors presented with severe neurological defects such as seizures, inability to stand upright for long periods or walk, suggesting that *tweek* was involved in synaptic function.^27^ These results further support a causative role of LoF of *KIAA1109* in the phenotypes observed in families AL, SA1–SA3, and US. Consistent with this hypothesis, *KIAA1109* has higher expression in the pituitary, the cerebellum, and the cerebellar hemispheres according to GTex.^28^

**Figure 5:**
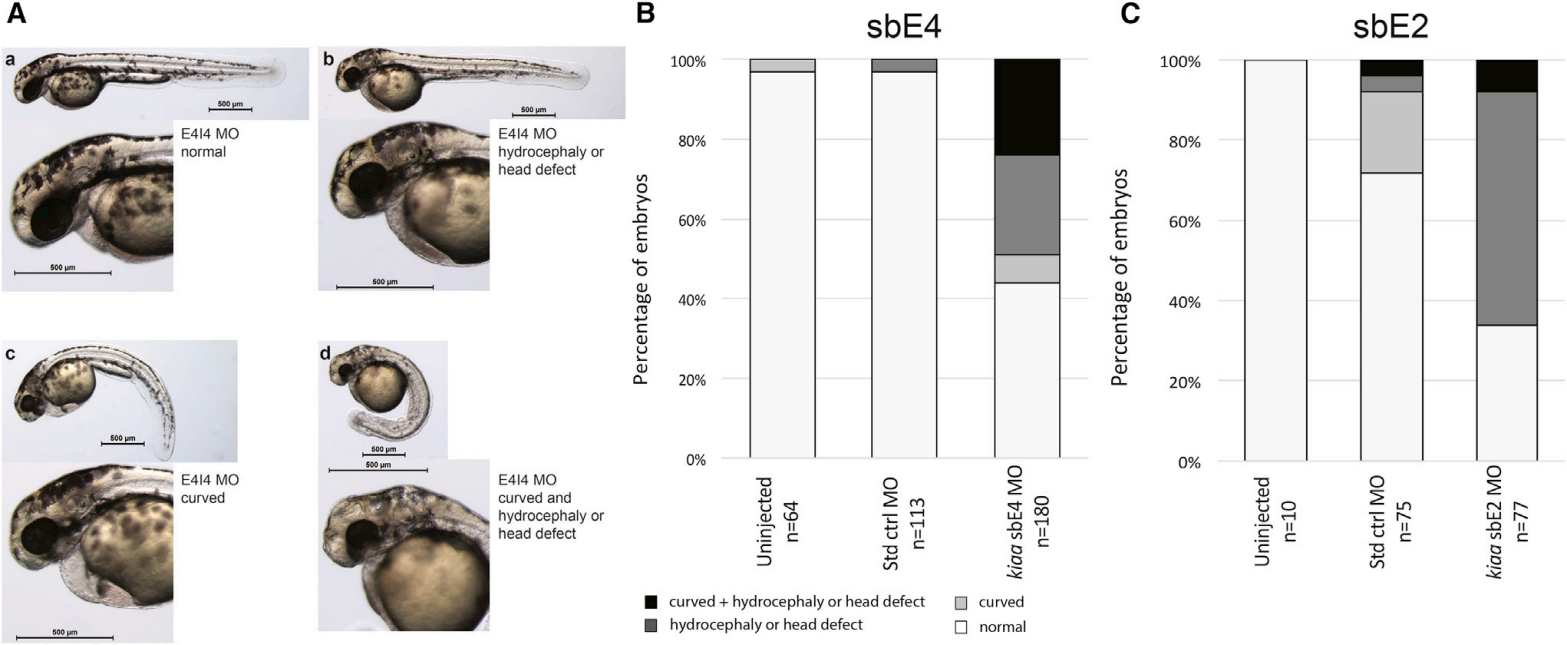
*kiaa1109* Knockdown in Zebrafish Results in Phenotypes Reminiscent of Probands’ Clinical Features (A) Lateral views representing the four classes of observed phenotypes in 2 dpf TU zebrafish embryos injected with sbE4-MO (morpholino) targeting *kiaa1109*: from top left to bottom right, normal, hydrocephalic or other head defects, curved and curved with head defect. (B) Results for uninjected embryos (left) and those injected with equivalent amounts of standard control MO (center) or *kiaa1109* sbE4-MO (6.7 ng, right). Phenotyping and scoring were performed at 2 dpf in two independent experiments. (C) Results for uninjected embryos (left) and those injected with equivalent amounts of standard control MO (center) or *kiaa1109* sbE2-MO (16.9 ng, right). Phenotyping and scoring were performed at 2 dpf in two independent experiments.

To further assess the consequences of decreased *KIAA1109* activity, we used both CRISPR/Cas9 genome editing and morpholinos (MO) technology in zebrafish. We generated three different stable lines with frameshift variants in exons 1, 4, and 7 of *kiaa1109*. Crosses of each heterozygote line with themselves suggest that these mutations are not lethal. To explain the discrepancy between these results and what was observed in mice and fruit flies, we profiled the transcriptome of homozygotes larvae. While we observed subtle differences between homozygous fish and their wild-type clutchmates, by and large we see no changes in expression of the different *kiaa1109* exons (Table S2). Our results suggest that the expression of *kiaa1109* isoforms containing only downstream exons encode proteins providing all the non-redundant functions of kiaa1109. More work is warranted to assess whether the engineered variants are inducing nonsense-mediated decay and whether there is any maternal contribution. In parallel, we knocked down *kiaa1109* using two different non-overlapping morpholinos (MOs). While we are aware that unspecific effects have been reported when using MOs,^29^ we still favored this approach to mimic to a certain degree the situation observed in the LT.II.1 and LT.II.2 siblings and the UK.II.1 affected individual. Injection of early zebrafish embryos with 6.7 ng of sbE4MO resulted in a 50% reduction of the *kiaa1109* transcripts through skipping of 65 nucleotides long exon 4 (Figure S7). 49% of morphants were hydrocephalic or presented with other head defects, whereas only 3% of the mock-injected fish showed such phenotypes (Figures 5A and 5B). The second MO, sbE2MO, acts through retention of 88-nucleotide-long intron 2 and decreases *kiaa1109* levels by about 50% after injection of a large dose of 16.9 ng (Figure S7). Under such condition, about two-thirds (66%) of MO-sbE2 morphants were hydrocephalic or presented with other head defects compared to 8% of mock-injected fish affected at such dose (Figures 5A and 5C). Of note, rescue experiments of MO-injected zebrafish could not be performed due to the large size of the *kiaa1109* transcript. In summary, knockdowns of the zebrafish *KIAA1109* ortholog using two different MOs resulted in hydrocephalic animals reminiscent of probands’ features.

## Discussion

Data aggregation of exome sequencing from multiple laboratories allowed associating homozygous and compound heterozygote variants in *KIAA1109* with a syndrome that we suggest naming Alkuraya-Kučinskas syndrome (AKS), as these clinicians first described affected individuals at the severe and mild ends of the phenotype, respectively. AKS combines severe brain malformations (13 affected individuals out of 13), in particular hydrocephaly/ventriculomegaly (11/13) and corpus callosum agenesis (8/13) with arthrogryposis/contractures (10/13) and/or talipes valgus/talipes equinovarus/club foot (12/13) and heart defects (6/13).

AKS presents multiple overlaps with Aase-Smith syndrome 1 (ASS1 [MIM: 147800]) characterized by arthrogryposis, hydrocephalus, Dandy-Walker malformation, talipes equinovarus, cardiac defects, and risks of stillbirth or premature death. However, the two described families with one father and two children affected^30^ and one mother and her affected daughter^31^ are suggestive of a dominant rather than a recessive mode of transmission. Consistent with the view that AKS and ASS1 have different etiologies, both ASS1-affected families presented individuals affected with cleft palate, a birth defect not present in the 13 *KIAA1109* individuals described here, including those at the severe end of the phenotypic spectrum. Interestingly, we have identified by exome sequencing an individual with partially overlapping features carrying a single *de novo* variant in *KIAA1109*. Although we cannot exclude that a second variant is present outside of the open reading frame, we might, alternatively, be dealing (1) with a spurious association or (2) with another syndrome related to AKS and associated with single variants in *KIAA1109*, similar to the *de novo* and biallelic variation recently associated with mitochondrial dynamics pathologies.^32^ The high missense ExAC Z-score of *KIAA1109* is compatible with such hypothesis. The identification of other similarly affected individuals will allow disentangling this conundrum.

The observed combination of intellectual disability, corpus callosum hypoplasia, hydrocephalus, and talipes equinovarus is also reminiscent of the constellation of features seen in the *L1CAM*-associated (neural cell adhesion molecule L1 [MIM: 308840]) HSAS (hydrocephalus due to congenital stenosis of aqueduct of sylvius [MIM: 307000]) and CRASH (corpus callosum hypoplasia, retardation, adducted thumbs, spastic paraplegia, and hydrocephalus [MIM: 303350]) syndromes. Whereas HSAS syndrome leads to neonatal or infant death, *L1CAM* variants survivors are described as affected by CRASH syndrome. Similarly, ten of the herein described AKS-affected case subjects did not survive past infancy (16 if accounting for undiagnosed miscarriages), whereas the living UK.II.1, LT.II.1, and LT.II.2 individuals presented other prominent features reported in CRASH syndrome, such as adducted thumbs, short stature, microcephaly, language impairment, and abnormalities of tone. L1CAM is a cell adhesion molecule that plays critical roles in neuronal migration and differentiation.^33^ Congenital joint contractures, limb deformities, hydrocephalus, corpus callosum agenesis, hypoplastic brainstem, cortical thinning, and high proportions of stillborn or neonatal death also are reminiscent of the PVHH (proliferative vasculopathy and hydranencephaly-hydrocephaly [MIM: 225790]) syndrome, a recessive disorder caused by variant in the transmembrane calcium transporter, *FLVCR2* (feline leukemia virus subgroup C receptor 2 [MIM: 610865]).

The *Drosophila* ortholog of *KIAA1109* named *tweek* is widely expressed but enriched in the brain lobes and in the ventral nerve cord.^27^ Neuronal phosphatidylinositol-4,5-bisphosphate [PI(4,5)P(2)] levels are critical in restricting synaptic growth via localization and activation of presynaptic Wiscott-Aldrich syndrome protein/WASP, a phenomenon dependent on *tweek* but not on bone morphogenetic protein signaling.^34^ The 5,005-amino-acid-long KIAA1109 protein is conserved from nematodes to vertebrates (Figure S8) in spite of a lack of recognizable domains, with the exception of a 22-residue amino-terminal transmembrane segment and a small central coiled-coil of 22 residues. It is described by specialists as an unconstrained peptide thought to adopt a definite conformation upon binding to its interactors.^35^ Consistent with this hypothesis, multiple high-throughput protein-protein interactions screens coupling near-endogenous expression levels with quantitative proteomics and mass spectrometry have identified human or mouse KIAA1109 interactors. For example, CTNNB1 (catenin beta-1), a protein associated with a dominant form of intellectual disability (MIM: 615075), interacts with two separate regions of KIAA1109.^36^ Another set of experiments showed high-confidence interactions with BUB3, DNAJB1, and PTPA, three proteins implicated in cell division.^37^ BUB3 participates to the spindle-assembly checkpoint signaling and the establishment of kinetochore-microtubule attachments. It inhibits the ubiquitin ligase activity of the anaphase-promoting complex (APC/C) by phosphorylating its activator CDC2. PTPA, one of four major Ser/Thr phosphatases, negatively controls cell growth and division. DNAJB1 (a.k.a. HSP40) interacts with HSP70 and stimulates its ATPase activity and its association with HIP. Interestingly, lower-confidence KIAA1109 protein interactors include BAG2 that competes with HIP for binding to the HSC70/HSP70 ATPase domain, as well as DRC1 and SMAD2. *DRC1* (MIM: 615288) encodes a central component of the nexin-dynein complex that regulates the assembly of ciliary dynein and is associated with primary ciliary dyskinesia (MIM: 615294). *SMAD2* (MIM: 601366) regulates cell proliferation, apoptosis, and differentiation through mediation of TGF-β signaling.

### Conclusion

We propose that bi-allelic LoF and missense variants in *KIAA1109* cause an autosomal-recessive brain malformation disorder with cerebral parenchymal underdevelopment ranging from major cerebral parenchymal thinning with lissencephalic aspect to moderate parenchymal rarefaction, severe to mild ventriculomegaly, and cerebellar hypoplasia with brainstem dysgenesis, associated with club foot and arthrogryposis. Severe cases are incompatible with life. Although further studies have to be engaged, our findings suggest that *KIAA1109* is potentially involved in cell cycle control, particularly of the central nervous system.

## Accession Numbers

Alkuraya-Kucinskas syndrome as described in this paper has been assigned MIM: 617822.

## Web Resources

1000 Genomes, http://browser.1000genomes.org/index.html
Burrows-Wheeler Aligner, http://bio-bwa.sourceforge.net/
dbSNP, https://www.ncbi.nlm.nih.gov/projects/SNP/
Ensembl genome assembly GRCh37, http://grch37.ensembl.org/Homo_sapiens/Info/Index
ExAC Browser, http://exac.broadinstitute.org/
ExomeDepth, https://cran.r-project.org/web/packages/ExomeDepth/index.html
GATK, https://software.broadinstitute.org/gatk/
GenBank, https://www.ncbi.nlm.nih.gov/genbank/
GTEx Portal, https://www.gtexportal.org/home/
NHLBI Exome Sequencing Project (ESP) Exome Variant Server, http://evs.gs.washington.edu/EVS/
OMIM, http://www.omim.org/
Picard, http://broadinstitute.github.io/picard/
PolyPhen-2, http://genetics.bwh.harvard.edu/pph2/
SIFT, http://sift.bii.a-star.edu.sg/
SnpEff, http://snpeff.sourceforge.net/
Splicing Finder, http://www.umd.be/HSF3/HSF.html
Swiss PDB Viewer, https://spdbv.vital-it.ch/

## Supplemental Data

Document S1. Supplemental Note, Figures S1–S8, and Table S1

Table S2. RPKM of kiaa1109 (ENSDARG00000062330) Exons in CRISPR-Cas9-Engineered Mutants and Their Wild-Type (WT) Clutchmate Larvae

Document S2. Article plus Supplemental Data
