# Supplementary material for: KIAA1109 Variants Are Associated with a Severe Disorder of Brain Development and Arthrogryposis
Source: Am J Hum Genet. 2017 Dec 28;102(1):116–32. doi: 10.1016/j.ajhg.2017.12.002 (PMC5777449; doi:10.1016/j.ajhg.2017.12.002)
Supplement: Document S1. Supplemental Note, Figures S1–S8, and Table S1 [file mmc1.pdf]

The American Journal of Human Genetics, Volume *102*

Supplemental Data

KIAA1109 Variants Are Associated with a Severe Disorder of Brain Development and Arthrogryposis

Lucie Gueneau, Richard J. Fish, Hanan E. Shamseldin, Norine Voisin, Frédéric Tran Mau-Them, Egle Preiksaitiene, Glen R. Monroe, Angeline Lai, Audrey Putoux, Fabienne Allias, Qamariya Ambusaidi, Laima Ambrozaityte, Loreta Cimbalistienė, Julien Delafontaine, Nicolas Guex, Mais Hashem, Wesam Kurdi, Saumya Shekhar Jamuar, Lim J. Ying, Carine Bonnard, Tommaso Pippucci, Sylvain Pradervand, Bernd Roechert, Peter M. van Hasselt, Michaël Wiederkehr, Caroline F. Wright, DDD Study, Ioannis Xenarios, Gijs van Haaften, Charles Shaw-Smith, Erica M. Schindewolf, Marguerite Neerman-Arbez, Damien Sanlaville, Gaëtan Lesca, Laurent Guibaud, Bruno Reversade, Jamel Chelly, Vaidutis Kučinskas, Fowzan S. Alkuraya, and Alexandre Reymond

Supplemental note: case reports

Detailed description of affected individuals

LT Family:

The elder brother of the Lithuanian family LT.II.1 was born at 40 weeks of gestation. His birth weight was 4100 g (75 centile), his height was 55 cm (90 centile), and the Apgar score was 9 at 1 minute and 10 at 5 minute. Movements of the eyes in up direction were noted from 3 months of age. EEG was normal. At age of 13 months paroxysmal loss of consciousness with drooling, eyes rolling up, repetitive blinking, and muscle spasms had started. They lasted 3-10 minutes and occurred once a week or two. The proband was treated with valproic acid, nitrazepamum, diazepamum. Later lamotrigin and topiromate were added. The last paroxysm was at age of 3.5 years. Brain MRI at age of 8 years revealed small posterior fossa arachnoid cyst, discrete vermian atrophy and slight increase of the fluid-filled retro and infra-cerebellar space as well as mild enlargement of subarachnoid spaces of frontal regions. The boy has hypermetropia (+5.5), strabismus and astigmatism. Global psychomotor retardation and hypotonia were noted from two months of age. The proband could sit independently and walk for a few steps with support until age of 10 years. Later in life, his motor abilities declined and he could not stand without support. Spontaneous paroxysms of laughter manifested from 2 years of age. He shows stereotypic movements as arm flapping or waving, and rhythmic body rocking. Additional clinical features are delayed eruption of permanent teeth, enuresis and encopresis. At 13 years and 8 months his head circumference was 53 cm (10 centile), his weight 35 kg (3-10 centile) and height 154 cm (10-25 centile). He presented with muscular hypotonia, plagiocephaly, strabismus, fat deposit in pubis, mild contractures of large joints, hypermobile small joints, talipes valgus, partial cutaneous syndactyly of 2nd and 3rd toes and a hypoplastic scrotum. He has no speech and severe ID (IQ<35). Array-CGH with Agilent 44K revealed no potentially pathogenic genomic structural abnormalities.

The younger sister LT.II.2 was born from an uncomplicated pregnancy at 38 weeks of gestation. Her birth weight was 4200 g (90 centile), Apgar 9, 9. She had clubfoot and her hands and feet were in paretic position. Her affected brother showed a similar posture after birth. Myoclonic movements of arms have been noted few days after birth. Hypotonia, movements of the eyes in up direction, nystagmus and strabismus manifested during the first month. At age of 2 months brain CT and EEG

showed no abnormalities. Paroxysmal loss of consciousness with drooling, eyes rolling up, repetitive blinking, and muscle spasms manifested at age of 10 months. Brain MRI at age of 1 year 8 months showed enlargement of subarachnoid spaces facing discrete parenchymal rarefaction involving the frontal lobes. Hypermetropia (+6), strabismus and astigmatism were diagnosed by an ophthalmologist. Psychomotor retardation was noted from birth. She could roll over at age of 5 years, could never sit without support or stand. She has problems with developing the ability to chew food and has chronic constipation. She showed stereotypic movements as arm flapping or waving. She has excessive drooling and frequently grinds her teeth. At 7 years and 5 months her head circumference was 49 cm (3-10 centile), her weight 25 cm (50 centile) and her height 134 cm (90 centile). She presented with muscular hypotonia, plagiocephaly, strabismus, mild contractures of large joints, talipes valgus and psoriasis. She has no speech and severe ID (IQ<35). Array-CGH with Agilent 105K revealed no potentially pathogenic genomic structural abnormalities.

AL Family:

The AL family pregnancy was the first one of healthy consanguineous parents. It was marked by the discovery at 21 WA (weeks of amenorrhea) of multiple cerebral malformation including triventricular hydrocephalus and thalamic fusion and bilateral equinovarus foot by ultrasound, which resulted in decision to terminate the pregnancy. Measurements were in the normal range according to a 22 WA fetus, i.e. occipital frontal circumference (OFC) = 21cm (+2SD), weight= 478g, talus-vertex length = 27cm, except for the brain weight (44g <10th percentile). A thorough examination of the AL.II.1 fetus revealed an arthrogryposis of the upper and lower limbs with crisped hands, adductus thumbs and bilateral equinovarus feet. The facial dysmorphism encompassed hypertelorism, a big horizontal mouth with retrognathism and miss-oriented ears. A scrotal hypoplasia and choane atresia was found. A slightly diffuse sero-hemorragic effusion was noticed. Neuropathological examination demonstrated, on the supra-tentorial space, both absence of cortical lamination and diffuse migration anomalies within a thin parenchymal mantle, as well as ventriculomegaly and voluminous germinal matrix. Corpus callosum was not identified. On infra-tentorial space, pathological findings include hypoplasia of the pons with absence of the longitudinal and transversal fibers and dysplasia of the

cerebellum characterized by lack of foliation and poorly identified vermis as well as narrowing of the aqueduct. Note that gyration analysis could not be performed due to poor conservation. Other tissues analysis revealed a dilatation of lymph vessels in the fibrous septa of the lung suggestive of a pulmonary lymphangiectasia and bilateral cataract with crystalline fibers of variable size and orientation. Skeletal X-rays examinations were normal. Standard blood chromosomes and a 60K quator PréCytoNEMv2 array-CGH (Agilent Technologies, CA, USA) were normal.

TU1 family:

The proband TU1.II.1 was the first child of first cousins parents from Tunisia. While her mother is healthy, her father is unable to read and write. He is epileptic with cognitive impairment secondary to encephalitis occurring at the age of five years. His MRI performed at 27 years was normal. During pregnancy, hypoplastic left heart, arthrogryposis with club feet and cerebral abnormalities were found. Prenatal imaging (ultrasound and MRI) showed on the infra-tentorial space cerebellar hypoplasia and brainstem dysgenesis characterized by flat and elongated pons and slightly kinked brainstem with increased fluid-filled retro-cerebellar spaces. Supra-tentorial anomalies include severe parenchymal (or cerebral mantle) thinning with major lack of gyration (lissencephalic aspect) associated with voluminous germinal matrix protruding within moderate ventriculomegaly and absence of corpus callosum. Cephalic biometry was normal. She was born at 34 GW. She presented facial dysmorphism with hypotelorism, deep palate, long fingers and left club foot. Cardiac ultrasound confirmed left hypoplastic heart with mitral and aortic atresia. Cerebral ultrasound confirmed antenatal ascertainments. Karyotype and FISH 22q11.2 were normal. She died at 3 days of life. Pathological examination was not performed according to parental decision.

This first pregnancy was followed by two spontaneous miscarriages (TU1.II.2 and TU1.II.3). During the fourth pregnancy of this couple, the mother was referred due to recurrence of a polymalformative fetus (TU1.II.4) characterized on ultrasound examination at 22 weeks by severe parenchymal thinning with lack of gyration associated with ventriculomegaly and corpus callosum agenesis. Extra-cerebral findings included arthrogryposis but a normal cardiac anatomy. The pregnancy was terminated at 23 GW. Pathological examination showed clenched hands with bilateral

camptodactyly, bilateral clubfeet, shoulder and hip joints contractures. No visceral malformation was observed. Brain examination showed a complete agenesis of the corpus callosum, ventricular dilatation, severe cortical malformations with a reduced cortical plate and vermian agenesis. Hyperplastic germinal matrix was protruding within ventricles. Neuropathological examination showed dysplasia of brainstem and cerebellum with neuroglial ectopia. At the supra-tentorial level, no callosal fibers were identified. The cortical plate showed neuronal depletion, numerous foci of heterotopia were observed within white matter. The three next pregnancies of the couple resulted in three healthy children.

TU2 family:

TU2.II.2 was the second child of consanguineous parents from Tunisia with no known genealogical links with the TU1 family. Prenatal imaging (ultrasound and MRI) showed on the infra-tentorial space both cerebellar hypoplasia and dysgenesis associated to severe brainstem dysgenesis characterized by flat and elongated pons and slightly kinked brainstem with increased fluid-filled retro-cerebellar spaces. Corpus callosum could not be identified. Supra-tentorial anomalies include severe parenchymal thinning with major lack of gyration demonstrating a pseudo-lissencephalic aspect as well as voluminous germinal matrix protruding within severe ventriculomegaly. Moreover, microphthalmia and club feet were present. She was born at 37 GW with a birth weight of 3100g, birth length of 45 cm and head circumference of 33 cm. She presented with microphthalmia, blepharophimosis, narrow chest, club feet and hands. She was hypotonic, with a reduced mobility and feeding and sucking difficulties. Post-natal MR confirmed prenatal data showing both thin cortical ribbon and global parenchymal thinning with lissencephalic aspect as well as bands of gray matter situated between the lateral ventricle and cerebral cortex, suggestive of neuronal migration disorder. This examination demonstrated also multiple germinolytic cysts within bilateral voluminous residual germinal matrix protruding in a severe dilated ventricular system. On infra-tentorial space, cerebellar and brainstem dysgenesis were confirmed associated with increased fluid-filled retro-cerebellar spaces and showed also a large arachnoid cyst responsible for mass effect on the distal part of the cerebellar tentorium. She died at 12 days of life, secondary to hypoventilation in a context of pulmonary hypoplasia. Autopsy was not performed according to parental decision. High-throughput sequencing of a panel of

29 genes involved in cortical malformation failed to pinpoint possibly causative variants.

UK family:

The British proband UK.II.1 (DDD #263241) is a 11 year old girl. She presented global developmental delay, behavioral problems (poor concentration, immaturity and minor self-harm when angry or frustrated) with mild to moderate learning disability allowing her to be in mainstream school but 2 years behind her peers. Prenatal imaging, including MRI, showed major microcephaly (HC -5 SD) with reduced white matter volume and mild ventriculomegaly but no abnormality of the cerebral hemispheres and no midline abnormalities. There was hypertelorism, slightly upslanting palpable fissures and oculo-motor apraxia, hypermetropia and strabismus. Dental crowding and high palate were also noticed. Skeletal abnormalities were also observed in this proband such as asymmetry of the thorax, mild bilateral talipes managed by physiotherapy, syndactyly of the 2nd and 3rd toes, 5th toe clinodactyly and hallux valgus. Finally, she had a complex congenital heart disorder with tetralogy of Fallot and pulmonary atresia, along with gastroesophageal reflux.

SA1 family:

The detailed phenotype of this individual was described in reference 13. Briefly, the SA1.II.1 fetus had severe ventriculomegaly with supra-tentorial cerebral mantle thinning associated with cerebellar hypoplasia, pleural effusion, severe arthrogryposis (fixed elbows, fixed bilateral talipes, bilateral overlapping fingers, bilateral clinodactyly, and bilateral club foot), low set ears, small eyes and micrognathia. The baby died after around one hour of delivery in the NICU. Parents are first cousins and they have had a previously affected child who is now deceased.

SA2 family:

First cousin Saudi parents presented to King Faisal Specialist Hospital & Research Center (KFSHRC) Maternal-Fetal Medicine for further evaluation when a screening ultrasound at a local hospital revealed multiple fetal anomalies of SA2.II.1. Ultrasonographic assessment at 18 weeks revealed hydrocephalus with hypoplastic cerebellum, absent corpus callosum, and shortened upper and lower limb bones. Multiple flexion deformities were noted. Heart could not be visualized but the chest,

abdomen and face appeared normal. No fetal movements were detected. Delivery was induced and resulted in a stillbirth with severe hydrocephalus and arthrogryposis multiplex. Babygram revealed poorly ossified calvarium and mildly shortened long bones. Parents did not authorize clinical photographs or autopsy.

SA3 family:

First cousin Saudi parents presented to KFSHRC Maternal-Fetal Medicine because of two previous intrauterine fetal deaths diagnosed with severe hydrocephalus and multiple skeletal anomalies (no records available). Ultrasonographic assessment of SA3.II.1 at 30 weeks revealed intrauterine fetal death with absent heart activity. Hydrocephalus, hypoplastic cerebellum, multiple flexion deformities and skin edema were noticed. Labor was induced and a dead fetus was delivered with severe hydrocephalus and arthrogryposis. The family declined clinical photographs or autopsy.

US Family:

The US family pregnancy was the third of healthy non-consanguineous parents. The couple's first pregnancy (US.II.1) resulted in a spontaneous miscarriage of unknown etiology at 11 weeks, cytogenetic testing revealed a normal 46, XY karyotype. The couple's second pregnancy (US.II.2) resulted in a spontaneous miscarriage at 20 weeks, secondary to findings of cystic hygroma, hydrocephaly, clinodactyly and talipes. Cytogenetic testing revealed 46, XY karyotype. No other testing was performed. The US.II.3 affected fetus was evaluated at 18 weeks 5 days gestation for concern for multiple congenital anomalies including cystic hygroma, ventriculomegaly, echogenic bowel, low conus and closed spinal defect with splayed vertebral arches at L4-L5, small liver only omphalocele, non-immune hydrops with scalp edema, bilateral pleural effusions, possible anal atresia, talipes, hyperflexed wrists, bilateral clinodactyly, low-set ears, short penis with bulbous shaft identified by ultrasound. High resolution fetal MRI detected ventriculomegaly, narrowing of the frontal lateral ventricles, thin cortical mantle, abnormal hypo-intense basal ganglia, absent corpus callosum and 3rd ventricle, kinked brainstem, hypoplasia or the cerebral hemispheres, and arthrogryposis. Fetal echocardiogram revealed a coarctation of the aorta. Additionally, during the evaluation the fetus was exhibiting

abnormal umbilical artery dopplers showing decreased end diastolic flow. The family elected for pregnancy termination and autopsy. A thorough neurological and genetic autopsy was completed. Physical examination detected severe arthrogryposis with flexion contractures and pterygia formation in all joints of the extremities as well as muscle atrophy, webbed neck, partially malrotated bowel and small placenta. Evaluation of the brain was significant for hydrocephaly, diaphanous pallium, small cerebellum and brainstem, heterotopic grey tissue, thickened basal meninges, all concerning for a migration defect and brainstem hypoplasia. Prenatal genetic testing included a normal 46, XY male karyotype via chorionic villus sampling.

SG Family:

The Singaporean siblings were two sons born to healthy, unrelated parents. The elder SG.II.1 brother was born at 34 weeks of gestation, the product of the couple's first pregnancy. Prenatally, severe ventriculomegaly was detected on ultrasound scan and fetal magnetic resonance imaging (MRI). Prenatal genetic testing included a normal male karyotype (46,XY) via amniocentesis. At birth, his weight was 2425 g (50-90th centile), his length was 43 cm (10-50th centile) and his head circumference was 38 cm (4 cm > 97th centile). He had minimal respiratory effort at birth and required immediate intubation and mechanical ventilation. On examination, macrocephaly, hypertelorism, posteriorly rotated ears, flattened nasal bridge, and excess skin fold of the neck were observed. He had generalized arthrogryposis, involving bilateral shoulders, elbows, wrists, hands and knees. He also had bilateral structural congenital talipes equinovarus (CTEV). Eye examination showed bilateral congenital cataracts and microphthalmia. He also had hypotonia, an ano-rectal malformation with recto-perianal fistula, and a small atrial septal defect/patent foramen ovale. Cerebral MR demonstrated severe ventriculomegaly with decreased pericerebral spaces, severe parenchymal thinning and smooth cortical surface, germinolysis cysts involving voluminous germinal matrix protruding within lateral ventricles as well as non identification of corpus callosum. Infra-tentorial findings include severe cerebellar hypoplasia with severe brain-stem dysgenesis characterized by a kinking aspect. Array-CGH with Agilent 44K revealed no potentially pathogenic genomic structural abnormalities. A ventriculo-peritoneal shunt was inserted at 2 months of age. He did not have adequate spontaneous respiration and was ventilator-dependent from birth, apart from two days when a trial of

extubation was done. He passed away at 3 months of age, from pneumonia and septic shock.

The next two pregnancies of the parents resulted in miscarriages (SG.II.2 and SG.II.3). Their fourth pregnancy resulted in the birth of the SG.II.4 younger brother, also at 34 weeks of gestation. Antenatally, bilateral ventriculomegaly, and club feet and hands were observed on ultrasound scan. At birth, his weight was 2130 g (10-50th centile), his length was 41 cm (10th centile) and his head circumference was 38.5 cm (4.5cm > 97th centile). His clinical findings were remarkably similar to his elder brother, with similar facial features (hypertelorism, bilateral low-set ears, short nose, anteverted nares), webbed neck, generalized arthrogryposis (involving bilateral elbows, wrists, hips and knees), bilateral structural congenital talipes equinovarus (CTEV), bilateral congenital cataracts and microphthalmia, and hypotonia. However, the younger brother did not have an ano-rectal malformation, and his cardiac defect was a fenestrated atrial septal defect. Magnetic resonance imaging (MRI) of the brain showed imaging findings similar to those that were described on the index case. He also remained ventilator-dependent from birth. The parents decided on withdrawal of invasive ventilation, and the baby passed away at 1 month of age.

Figure S1

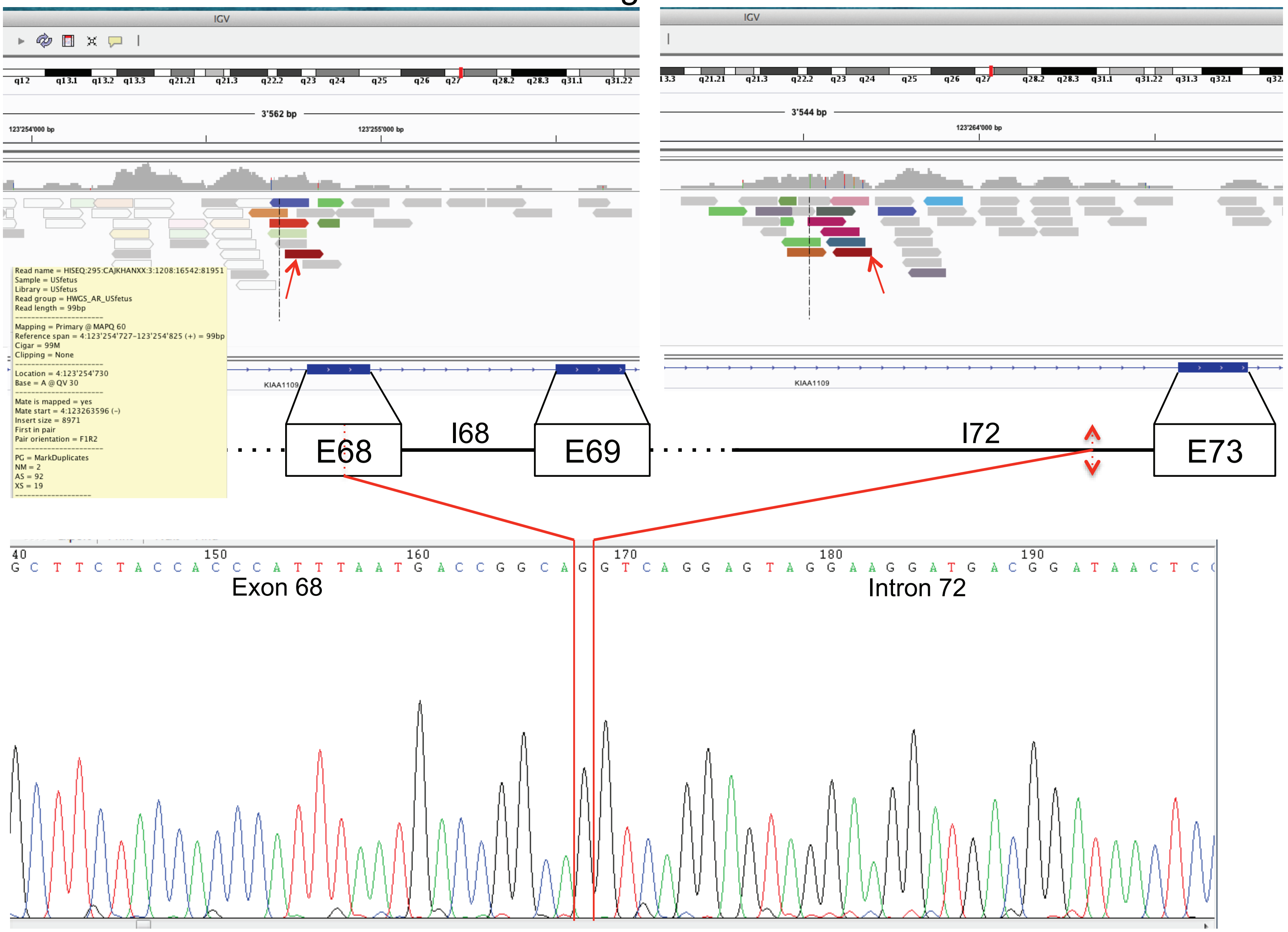

IGV
3'562 bp
123'254'000 bp
123'255'000 bp
Read name = HISEQ:295:CAJKHANXX:3:1208:16542:81951
Sample = USfetus
Library = USfetus
Read group = HWGS_AR_USfetus
Read length = 99bp
Mapping = Primary @ MAPQ 60
Reference span = 4:123'254'727-123'254'825 (+) = 99bp
Cigar = 99M
Clipping = None
Location = 4:123'254'730
Base = A @ QV 30
Mate is mapped = yes
Mate start = 4:123263596 (-)
Insert size = 8971
First in pair
Pair orientation = F1R2
PG = MarkDuplicates
NM = 2
AS = 92
XS = 19
KIAA1109
3'544 bp
123'264'000 bp
KIAA1109
E68
I68
E69
I72
E73
Exon 68
Intron 72

Figure S1: Breakpoint mapping of the US family large deletion

From top to bottom: whole genome paired-end sequences of the US.II.3 fetus are visualized on the Integrative Genomics Viewer (IGV). Reads aligning to *KIAA1109* intron 67, exon 68 (E68), intron 68 (I68), exon 69 (E69) (left panel), intron 72 (I72) and exon 73 (E73) (right panel) are schematically shown. A single pair of reads (chestnut brown pointed-head rectangles pinpointed by red arrows) mapping unequivocally 8971 bp apart was identified (see yellow inset) allowing to narrow down the mapping of the breakpoints of the paternally-inherited deletion of the US.II.3 proband. They were then finely mapped using PCR and Sanger sequencing to coordinates chr4:123,254,885 (hg19) within E68 and chr4:123,263,438 within I72 respectively with the insertion of a guanine nucleotide. The breakpoints are depicted by a dotted line in E68, a double pointed arrow in I72 and two vertical red lines on each side of the inserted G nucleotide in the Sanger chromatogram, respectively.

Figure S2

Exon1

chr13:12,668,517-12,668,866

agtgtgcatttgcagtttacttgataagtgagaggctgatgcagaccctgtgtaatttgatagtgatgtccgctctgt
ttatctgcagtggggtgtaaccATGGATAAAGGCAACAACAGTCTCCCCACTTACGATGAAATAGATGAATACCTCAG
CAGGCGCAACTCCACCTTCGTGTGGCTCCTTGTGGgtaagtgatttatgatttgtgcttcgtctacactttgcaatca
gcacagttacagtctacactggaggtgcctagtagattgcattgagttttaaaggtacagttcacccaaaaatgaacc
ttttactgaacttacataaacctgtatgtgtcctcctt

Exon 4

chr13:12,671,867-12,672,216

acacacacacacacaaacacacgcaaataaacttttgagactctgcttattgctgttgtctgtgagacctttttcatt
tgatatgggttatatttcatctttatttactctgttgtttatatttgtcattttcagAATTCAGGATGGGTTACTCAT
ATTTCGTTGGTGGAAGATGTACAACCCAAAGCAGAAGCAACATGgtcagtgcaaaaacagaacaaacagcaaagaatt
gttcaatttgagcacatcatgtcagtctcaatagtctcagtctcagtacaccaatcccacaatatctgtaaatgcttg
tcattttagttgctctatggttgggtttatctgtgctt

Exon 7

chr13:12,683,417-12,683,816

aattgaatgtcttatttctatgagtgtcctatcctcaagtattgtgaccaaagaaaaacctaataaatattgactgac
attactctttctggttttgccttcagGGTCGCGTAGCATTTGGTAATCACCATCTTCCTCAGACCCTCTGCATGAACT
TTGACGATGCCTTCTTGACATATGCCACCAAACCACCCAGCAGCCATCTGGATCAGTTCATGCACATTGTGAAGGGTT
CATTGGAGAACGTTCGTGTCATGCTGGTGCCCAGTCCACGATACCTAGGCCTTCAGAATGACGAgtgagtcaaataat
ctccagtatatcagattagattagattctactttattgtcattgggcaggaatagagccatttaaaggatattcacc
aaaaaattac

Figure S2: Zebrafish *kiaa1109* editing target sites

CRISPR Cas9 target sites in exons 1, 4 and 7 of zebrafish *kiaa1109* are shown. Exon numbering and chromosome 13 coordinates according to NM_001145584.1 and zebrafish genome assembly GRCz10/danRer10, respectively. Exon sequences are in blue and upper case, nucleotides at exon edges are in light blue. sgRNA target sites are underlined with PAM sequences boxed. The expected position for Cas9 DNA cleavage and mutations introduced by non-homologous end joining is between the two red nucleotides. The position for annealing of genotyping oligonucleotides is highlighted yellow; with the reverse oligonucleotide having the reverse complement of the second highlighted sequence (one DNA strand is represented).

Figure S3

A. Exon 1

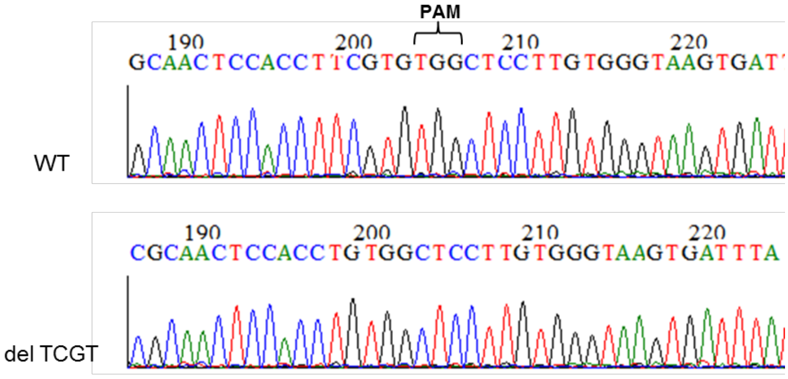

Reference sequence from exons 1 and 2.
Exon border nucleotides in blue, PAM in red

```
atggataaaggcaacaacagtctccccacttacgatgaaatagatgaatacctcagcagg
 M  D  K  G  N  N  S  L  P  T  Y  D  E  I  D  E  Y  L  S  R
cgcaactccaccttcgtgtggctccttgtggccactattatgtcatgcggatggatcata
 R  N  S  T  F  V  W  L  L  V  A  T  I  M  S  C  G  W  I  I
tatctgacatactacaattctcgcaatattggcctcgtcctcaccctcatcattaaccga
 Y  L  T  Y  Y  N  S  R  N  I  G  L  V  L  T  L  I  I  N  R
ctctacaagaatggatacatacacatcg
 L  Y  K  N  G  Y  I  H  I
```

Mutation in exon 1 (deletion of TCGT), NM_001145584.1:c.74_77del

```
atggataaaggcaacaacagtctccccacttacgatgaaatagatgaatacctcagcagg
 M  D  K  G  N  N  S  L  P  T  Y  D  E  I  D  E  Y  L  S  R
cgcaactccacctgtggctccttgtggccactattatgtcatgcggatggatcatatatc
 R  N  S  T  C  G  S  L  W  P  L  L  C  H  A  D  G  S  Y  I
tgacatactacaattctcgcaatattggcctcgtcctcaccctcatcattaaccgactct
 -  H  T  T  I  L  A  I  L  A  S  S  S  P  S  S  L  T  D  S
acaagaatggatacatacacatcg
 T  R  M  D  T  Y  T  S
```

B. Exon 4

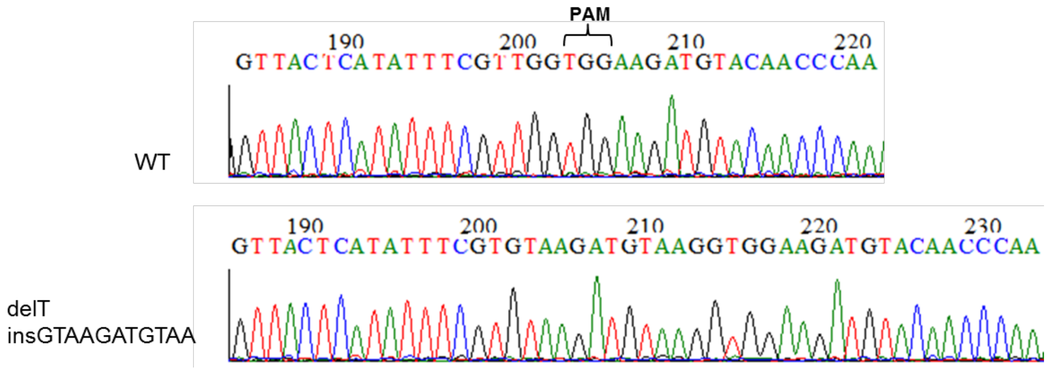

Reference sequence from exons 4 and 5.
Exon border nucleotides in blue, PAM in red

```
aattcaggatgggttactcatatttcgttggtggaagatgtacaacccaaagcagaagcaa
  I  Q  D  G  L  L  I  F  R  W  W  K  M  Y  N  P  K  Q  K  Q
catgacccgaaggctgagacgcgtctctatgttactgttaacggctttgagttccatgtt
 H  D  P  K  A  E  T  R  L  Y  V  T  V  N  G  F  E  F  H  V
tataatcggacggatctgtacactcggcttcaggaaatatttggcctcgagcccacccctt
 Y  N  R  T  D  L  Y  T  R  L  Q  E  I  F  G  L  E  P  T  L
atccaatccaaccgggatgaggagaaaggccgagaacagagggataaatccttggagag
 I  Q  S  N  R  D  E  E  K  G  R  E  Q  R  D  K  S  L  E
```

Mutation in exon 4 (indel, delT insGTAAGATGTAA),
NM_001145584.1:c.316delTinsGTAAGATGTAA

```
aattcaggatgggttactcatatttcgtgtaagatgtaaggtggaagatgtacaacccaaa
  I  Q  D  G  L  L  I  F  R  V  R  C  K  V  E  D  V  Q  P  K
gcagaagcaacatgacccgaaggctgagacgcgtctctatgttactgttaacggctttga
 A  E  A  T  -  P  E  G  -  D  A  S  L  C  Y  C  -  R  L  -
gttccatgtttataatcggacggatctgtacactcggcttcaggaaatatttggcctcga
 V  P  C  L  -  S  D  G  S  V  H  S  A  S  G  N  I  W  P  R
gcccaccccttatccaatccaaccgggatgaggagaaaggccgagaacagagggataaatc
 A  H  P  Y  P  I  Q  P  G  -  G  E  R  P  R  T  E  G  -  I
cttggagag
 L  G  E
```

C. Exon 7

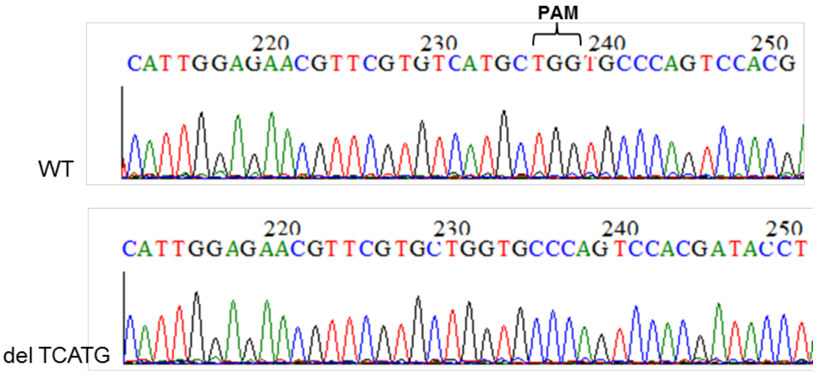

Reference sequence from exons 7 and 8.
Exon border nucleotides in blue, PAM in red

```
ggtcgcgtagcatttggtaatcaccatcttcctcagaccctctgcatgaactttgacgat
 G  R  V  A  F  G  N  H  H  L  P  Q  T  L  C  M  N  F  D  D
gccttcttgacatatgccaccaaaccacccagcagccatctggatcagttcatgcacatt
 A  F  L  T  Y  A  T  K  P  P  S  S  H  L  D  Q  F  M  H  I
gtgaagggttcattggagaacgttcgtgtcatgctggtgcccagtccacgatacctaggc
 V  K  G  S  L  E  N  V  R  V  M  L  V  P  S  P  R  Y  L  G
cttcagaatgacgaacctccgaggctcatgggtgagggatttgtggtcatgcagtcgaat
 L  Q  N  D  E  P  P  R  L  M  G  E  G  F  V  V  M  Q  S  N
gatgtggacatttactactatcaagatgaaccag
 D  V  D  I  Y  Y  Y  Q  D  E  P
```

Mutation in exon 7 (del TCATG), NM_001145584.1:c.758_762del

```
ggtcgcgtagcatttggtaatcaccatcttcctcagaccctctgcatgaactttgacgat
 G  R  V  A  F  G  N  H  H  L  P  Q  T  L  C  M  N  F  D  D
gccttcttgacatatgccaccaaaccacccagcagccatctggatcagttcatgcacatt
 A  F  L  T  Y  A  T  K  P  P  S  S  H  L  D  Q  F  M  H  I
gtgaagggttcattggagaacgttcgtgctggtgcccagtccacgatacctaggccttca
 V  K  G  S  L  E  N  V  R  A  G  A  Q  S  T  I  P  R  P  S
gaatgacgaacctccgaggctcatgggtgagggatttgtggtcatgcagtcgaatgatgt
 E  -  R  T  S  E  A  H  G  -  G  I  C  G  H  A  V  E  -  C
ggacatttactactatcaagatgaaccag
 G  H  L  L  S  R  -  T
```

Figure S3: Zebrafish *kiaa1109* variants

We generated three different stable zebrafish lines with frameshift variants, i.e. c.74_77del, c.316delTinsGTAAGATGTAA and c.758_762del in exons 1 **(A)**, 4 **(B)** and 7 **(C)** of *kiaa1109* (a.k.a. si:ch211-233a24.2, exon numbering and variant nomenclature according to NM_001145584.1). Representative sequencing chromatograms are shown for unedited (WT allele, top) and edited (mutant allele, bottom) sequences. The traces are from clones from genotyping PCRs, using tail clips of heterozygous adult fish. The predicted frameshift resulting from these mutations is given as a block of cDNA, with its single letter translated amino acid sequence under the DNA, in the regions of the editing target sites. Predicted stop codons are shown as hyphens.

Figure S4

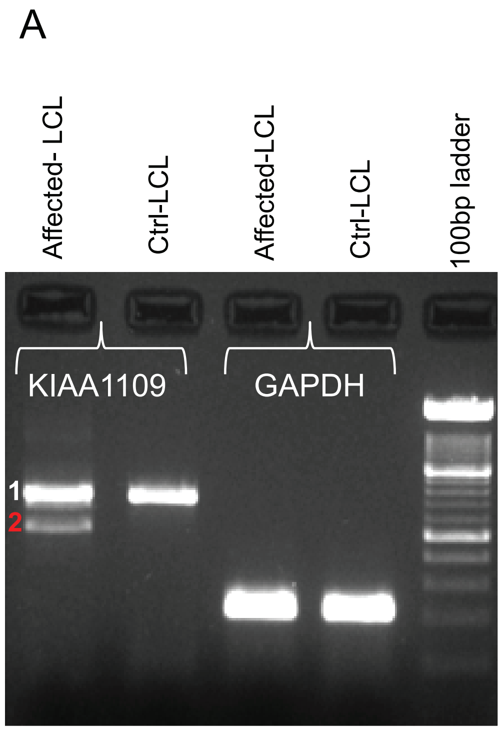
A
Affected- LCL
Ctrl-LCL
Affected-LCL
Ctrl-LCL
100bp ladder
KIAA1109
GAPDH
1
2

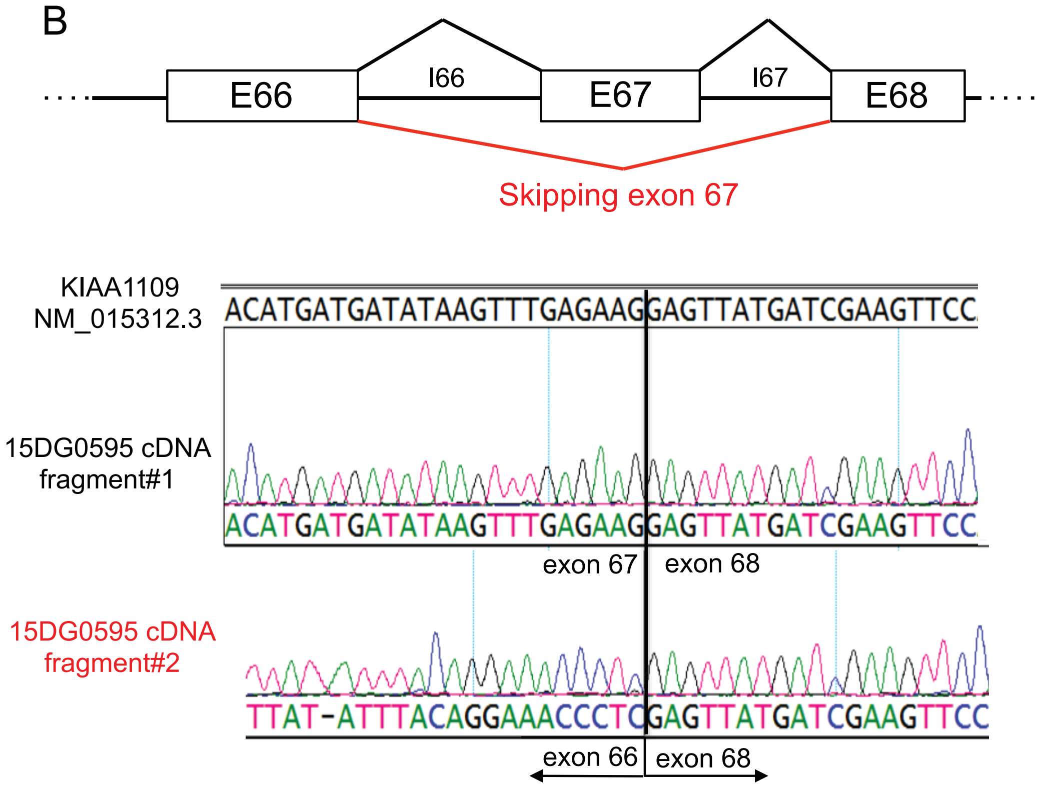
B
E66
I66
E67
I67
E68
Skipping exon 67
KIAA1109
NM_015312.3
ACATGATGATATAAGTTTGAGAAGGAGTTATGATCGAAGTTCC
15DG0595 cDNA
fragment#1
ACATGATGATATAAGTTTGAGAAGGAGTTATGATCGAAGTTCC
exon 67
exon 68
15DG0595 cDNA
fragment#2
TTAT-ATTTACAGGAAACCCTCGAGTTATGATCGAAGTTCC
exon 66
exon 68

Figure S4: The *KIAA1109* c.11250-1G>A variant induces skipping of exon 67 in the SA2.II.1 fetus.

(A) Agarose gel separation of RT-PCR amplicons of *KIAA1109* exons 66 to 68 from lymphoblastoid cell lines (LCL) from the affected SA2.II.1 fetus (affected-LCL) and a control individual (Crtl-LCL) (left). We observe two bands corresponding to an amplicon with all exons (fragment #1) and an amplicon missing exon 67 (2) specifically in the LCLs of the affected fetus. *GAPDH* was used as positive control (right). **(B)** Sanger sequencing of fragment 1 (top) and 2 (bottom) amplicons from the affected-LCL compared with that of the Ctrl-LCL amplicon. Sanger sequencing of fragment 2 showed the skipping of exon 67.

Figure S5

```
                                              R968C
Human           --------FHVVCREYELERPKSVIICQHGIDRRFCESKLSCIPGPCPTSDDLKYTMIRL 995
Pig             --------FHVVCREYELERPKSVIVCQHGIDRRFCESKLSCIPGPCPTSDDLKYTMTRL 994
Mouse           --------FHVVCREYELERPKSVIVCQHGIDRRFCESKLSCIPGPCPTSDDLKYTMTRL 995
Opossum         --------FHVVCREFELERPKSVILCQHGIDRRFCESKLSCVPGPCSTSDELKYTMTRL 1000
Chicken         --------FHVVCREFELERPKSVIICQHGIDRRFCDSKMNCVPGPCPTSDDLKYTMTRL 994
Coelacanth      STVLFCHLLFSSCREFIEKSI-----------------KQICCLLPCEP--EVRYTFCYL 866
Zebrafish       --------FHVMSREFQLEQPKPSVTCQHGVDRRICDAKHAGLPGHCRTSEDLKYTMTRL 977
                        :.  .**:  :                   *       *    :::**:  *
                                                  Y1329C
Human           TQDKSVGQSPLRSPLKRQASVCSTRLGSTKSLTAAFYGDKQPVTVGVQFSSDVSRSDENV 1352
Pig             TQDKSVGQSPLRSPLKRQASVCSTRLGSTKSLTAAFYGDKQPVTVGVQFSSDVSRSDENV 1351
Mouse           TQDKSVGQSPLRSPLKRQASVCSTRLGSTKSLTAAFYGDKQPVTVGVQFSSDVSRSDENV 1352
Opossum         TQDKSGVQSPLRSPLKRQASVCSTRLGSTKSLTAAFYGDKQPVTVGVQFSSDVSRSDENV 1357
Chicken         SQERPVGQSPMRSPLKRQASVCSTRLGSTKSLTAAFYGDKQPVPVGVQFSSDVSRSDENV 1350
Coelacanth      SQDKPSVHSPLKSPLKRQASVCSTRLGSTKSLTAAFFGEKQPAPAGVQFSSEVSRSDENV 1218
Zebrafish       IPEGPPLRSPLRSPLKRQSSVQSARLGSTKSLSAAVFVEKALPPAGVQFSSEVSRSDENV 1307
                  :    :**::******:** *:********:**.: :*    .******:********
                                                          M1573I
Human           SLHRPLDLDTPTSEESSSSFEQLSVPTFKVIKQGLTANSLLDRGMQLSGSTSNTPYTPLE 1588
Pig             SLHRPLDLDTPTSEESSSSFEQLSVPTFKVIKQGLTANSLLDRGMQLSGSTSNTPYTPLE 1587
Mouse           SLHRPLDLDTPTSEESSSSFEQLCVPTFKVIKQGLTANSLLDRGMQLSGSTSNTPYTPLD 1588
Opossum         SLHRPLDLDTPTSEESSSSFEHLSVPTFKVIKQGLTANSLLDRGMQLTGTTSNTPYTPLE 1593
Chicken         SLHRPLDLDTPTSEESSSSFEQLSVPTFKIVKQGLTANSLLDRGMQLTGSTSNTPYTPLD 1588
Coelacanth      SLHRPLDLDTPTSEESSSSFDQLSVPTFKVVKQGLTANALLDRGVQLMGSTSTAPYTPLE 1456
Zebrafish       SLHRPLDLDTPTSEESSTCFDQLSIPTFKMVKAGLSASSLLDRGVQLMGDINSTPYTPLD 1534
                *****************:.*::*.:****::* **:*.:*****:** *  ..:*****:
                                                       V1867M
Human           VHGQLRGLDT-TDIGTCAITAIPFEKSKVLFTLEELDEFTFVDETDQQ--------AVPD 1877
Pig             VHGQLRGLDT-TDIGTCAITAIPFEKSKVLFTLEELDEFAFVDETDQQ--------AVPD 1875
Mouse           VHGQLRGLDT-TDIGTCAITAIPFEKSKVLFTLEELDEFTFVDETDQQ--------AIPD 1877
Opossum         VHGQLRGLDT-TDNGTCAITAIPFEKSKVLFTLEELDEFTFVDETDHQ--------AIPD 1881
Chicken         VHGQLRGLDT-TDIGTCAITAIPFGKSKVLFTLEELDEFTFVDETDQQ--------AVPD 1878
Coelacanth      IHGQLRGLDAAEDIGTCAITAIPFEKSKVLFTLEELDEFNFVDETEQQ--------NPSD 1745
Zebrafish       IHSQLRGLDS-SDIGACAITAIPFEKSKVLFSLEEIDDFVLVDETEPSISTEHMPEHNPD 1824
                :*.******:  * *:******** ******:***:*:* :****: .           *
                                  R1958Q
Human           DSPTGSGYNTDVSDDNLPCDRTSPSSDLNGNSVSDEQDEGVESDDLKKDLPLMPPPPDSC 1997
Pig             DSPTGSGYNTDVSDDNLPCDRTSPSSDLNGNSVSDEQDEGVESDDLKKDLPLMPPPPDSC 1995
Mouse           DSPTGSGYNTDVSDDNLPCDRTSPSSDINGNSVSDEQDEGVESDDLKKDLPLMPPPPDSC 1997
Opossum         DSPTGSGYNTDVSDDNLPCDRISPSSDINGNSISDEQDEGVESDDLKKDLPLMPPPPDSC 2001
Chicken         DSPTGSGYNTDVSEDNLPCDRISPSSDINGNSVSDEQDEGVESDDLKKDIPLMPPPPDSC 1998
Coelacanth      GSPTGSGYNTDVSDDNLPHDGVSPSSDNNGNSESDEQDEGVESDDLKKELPLMPPPPDSS 1865
Zebrafish       GSQTGSGYSTDVSDDNLPNDAQSPASEPNNNSDSDEQDEGVESDDLKKDLPLLPPPPDSS 1944
                .* *****.****:**** *  **:*: *.** ***************::**:******.
                                                                  P3050H
Human           KAEYKMGRMRSHGMTGAQTRFTFELPNHRLRFTSKVSATDMSTIPPSASLNLPPVTMSGK 3057
Pig             KAEYKMGRMRSHGMTGAQTRFTFELPNHRLRFTSKVSATDMSTIPPSASLNLPPVTMSGK 3055
Mouse           KAEYKMGRMRSHGMTGAQTRFTFELPNHRLRFTSKVSATDMSTIPPSASLNLPPVTMSGK 3057
Opossum         KAEYKMGRMRSHGMTGAQTRFTFELPNHRLRFTSKVSATDMSTIPPSASLNLPPVTMSGK 3062
Chicken         KAEYKMGRMRSHGMTGAQTRFNFELPSHRLRFTSKVSATDMSTIPPSASLNLPPVTMSGK 3056
Coelacanth      KAEYKMGRMKSHGMTGAQTRFTFELPNHRLRFTSKVSPVDMSTIPPSASLNLPPVTMSGE 2928
Zebrafish       KAEYKMGRMKSHGMTGAQTRFTFELPNHKLCFQSKVSPVDVSAMPPTASLTLPPVTMSGQ 2991
                *********:***********.****.*:* * **** .*:*::**:***.********:
                                           G3385R
Human           GDLDTGSALVLTIESTLITACSSESLVSKGHFKNFCIRFADGFETSWDDWKPEIHGDLVM 3415
Pig             GDLDTGSALVLTIESTLITACSSESLVSKGHFKNFCIRFADGFETSWDDWKPEIRGDLVM 3413
Mouse           GDLDTGSALVLTIESTLITACSSESLVSKGHFKNFCIRFADGFETSWDDWKPEIRGDLVM 3415
Opossum         GDLDTGSALVLTIESTLITACSSESLVSKGHFKNFCIRFADGFETTWDDWKPEIRGDLVM 3420
Chicken         ADLDTGSALVLTIESTLITACSSESLVSKGHFKNFCIRFADGFETSWDDWKPEIRGDLVM 3414
Coelacanth      VDFDAGSALVLTIESTLITACSSESLVSKGHFKNFCIRFADGFETTWDDWKPEVRTDLVM 3288
Zebrafish       IDFDTGSALVLTIESTLITACSSESLVSKGHFKNFCIRFAEGFETTWDDWKPEIRGDLVM 3344
                 *:*:***********************************:****:*******:: ****
```

Figure S5: The missense variants identified in the probands affect conserved *KIAA1109* residues.

Multialignments of the regions of KIAA1109 harboring the missense variants identified in the Singaporean (R968C), the Lithuanian (Y1329C and V1867M), the British (M1573I and R1958Q), the Algerian (P3050H) and the Tunisian (G3385R) affected individuals. The position of the putatively changed residue is boxed. The identified codon modifications are indicated above.

Figure S6

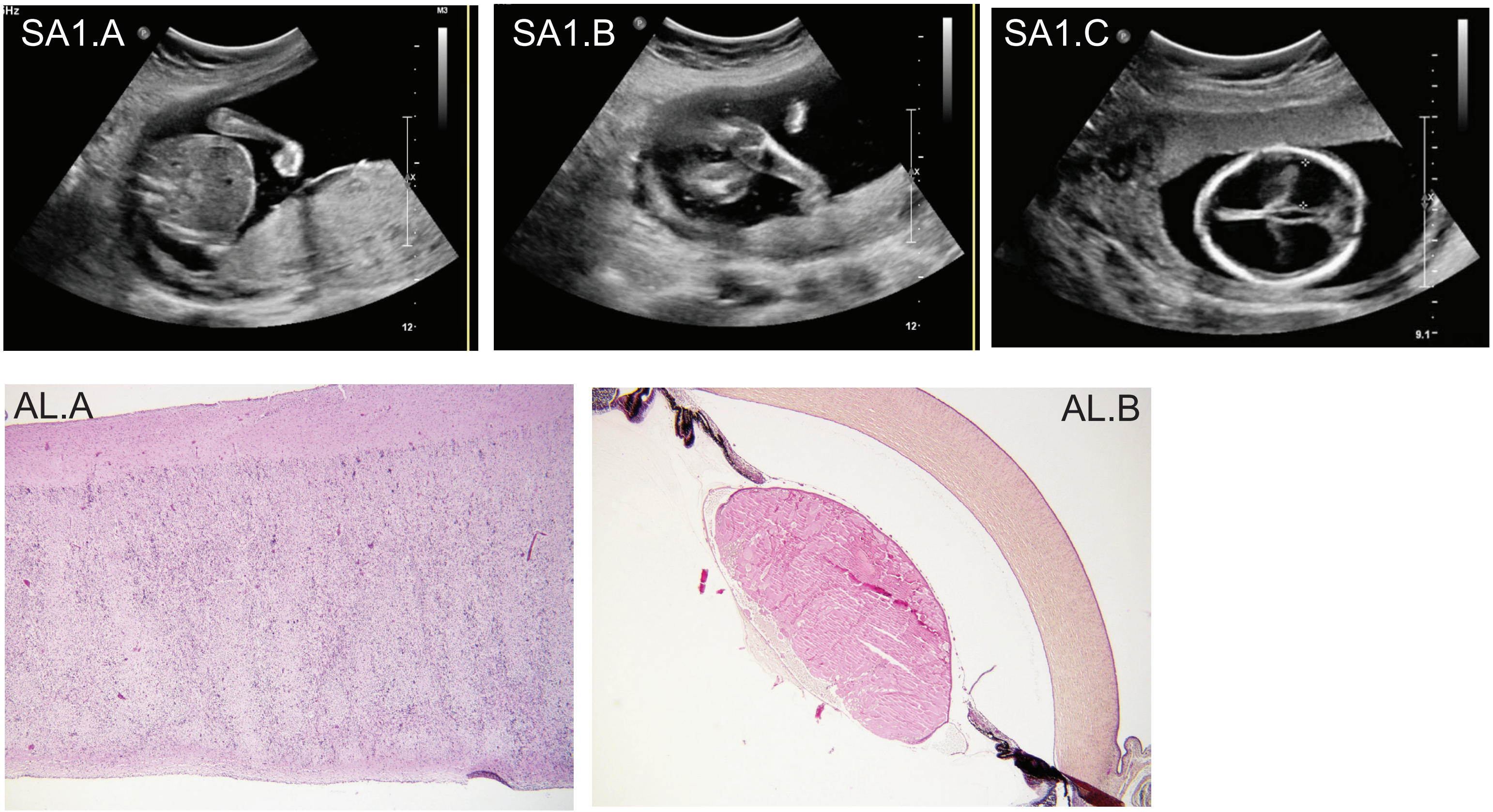

Figure S6. Ultrasound images of the SA1.II.1 and US.II.3 stillborn fetuses and histology pictures of AL.II.1.

SA1(A-C) Antenatal ultrasound scans from the SA1.II.1 fetus showing a fixed flexed hand **(A)**, a club feet **(B)** and cerebellar hypoplasia **(C)**.

AL(A-B) Histology pictures from the AL.II.1 fetus showing lamination defect **(A)** and cataract **(B).**

Figure S7

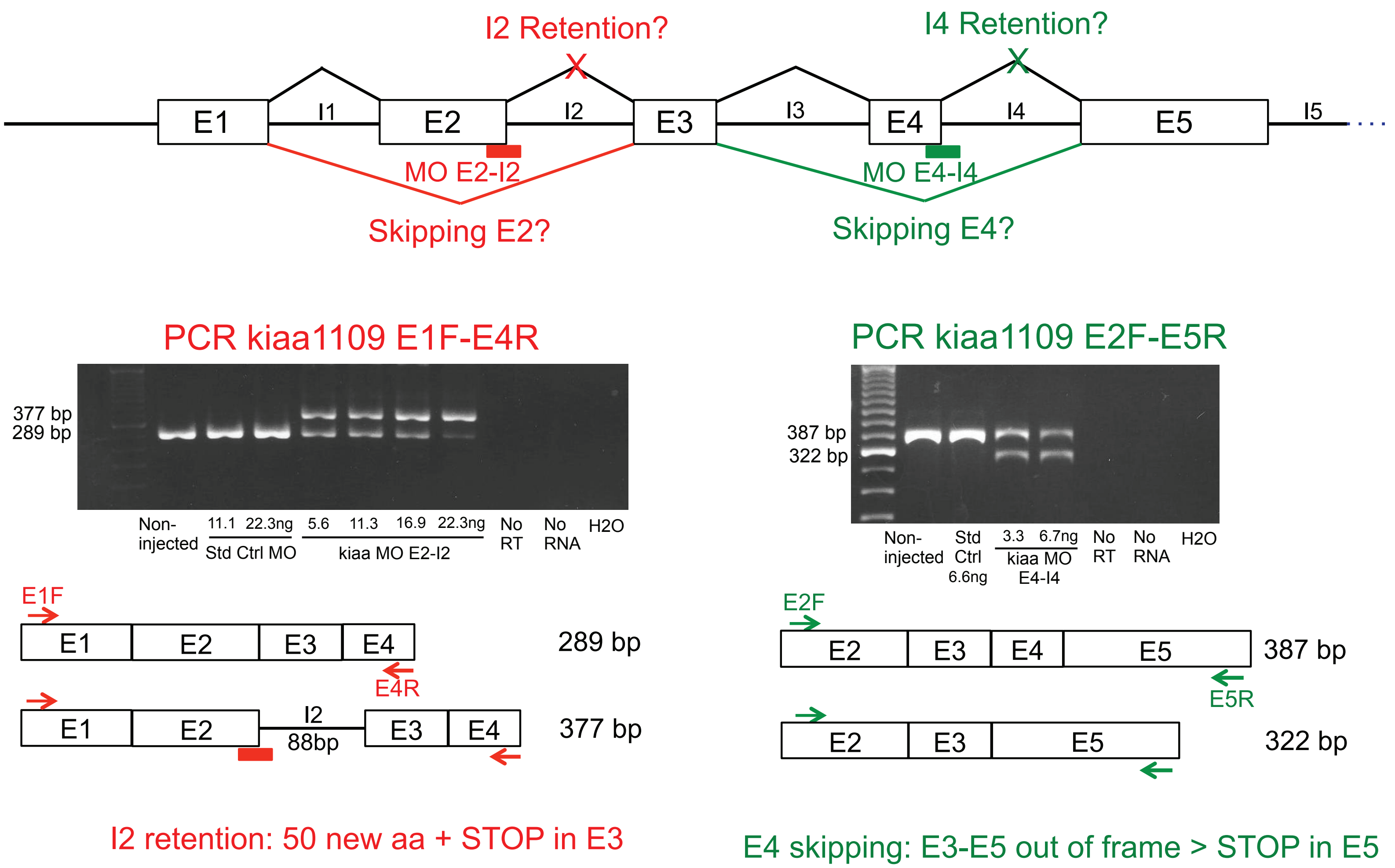
I2 Retention?
I4 Retention?
E1
I1
E2
I2
E3
I3
E4
I4
E5
I5
MO E2-I2
MO E4-I4
Skipping E2?
Skipping E4?
PCR kiaa1109 E1F-E4R
377 bp
289 bp
Non-injected
11.1 22.3ng
Std Ctrl MO
5.6 11.3 16.9 22.3ng
kiaa MO E2-I2
No RT
No RNA
H2O
E1F
E4R
289 bp
I2 88bp
377 bp
I2 retention: 50 new aa + STOP in E3
PCR kiaa1109 E2F-E5R
387 bp
322 bp
Non-injected
Std Ctrl 6.6ng
3.3 6.7ng
kiaa MO E4-I4
No RT
No RNA
H2O
E2F
E5R
387 bp
322 bp
E4 skipping: E3-E5 out of frame > STOP in E5

Figure S7: *kiaa1109* morpholino (MO) knockdown strategy in zebrafish.

The top panel shows the schematic representation of the exon-intron structure of the 5' portion of the zebrafish *kiaa1109*. The sites targeted by the two MOs (morpholinos) are indicated in red (sbMO E2-I2) and green (sbMO-E4-I4) and can result in either exon skipping or intron retention.

The consequences of injection of sbMO E2-I2 (left) and sbMO-E4-I4 (right) on the *kiaa1109* transcript are assessed by RT-PCR. The outcomes are schematically depicted at the bottom, i.e. retention of intron 2 (left) and skipping of exon 4 (right). The positions of the used primers (red and green arrows) and the sizes of the amplicons are indicated. The sequences of the used primers are as follow: E1F-5'-GAATACCTCAGCAGGCGCAA-3'; E4R-5'-GGTTGTACATCTTCCACCAACG-3'. E2F-5'- ATCTGACATACTACAATTCTCGCAA-3' and E5R-5'-TATCCCTCTGTTCTCGGCCT-3'. Different doses of both MO were tested. They resulted in the same abnormal transcripts with increased efficiency with increased doses. The agarose gel pictures presented in the middle panels show from left to right RT-PCR performed in uninjected, standard control-MO injected and kiaa1109-MO injected embryos, respectively. The sbMO E2-I2 resulted in intron 2 retention whereas the sbMO E4-I4 resulted in exon 4 skipping. The 377bp band corresponds to the amplimer from the transcript keeping intron 2, whereas the 322bp band corresponds to the amplimer from the transcript skipping exon 4. Both MOs result in early stop codon and are predicted to encode truncated kiaa1109 proteins.

Supplementary Figure S8

A

```
H.sapiens           -----MDQRKNESIVPSITQLEDF---------LTEHNSNVVWLLVATI-LSCGWIIYLT 45
D.melanogaster      MEALEVEDTSDDSLPMGFPEVGTWNSTHNVSLNDMNLDARMIWLLASLL-TTITWVTYIT 59
C.elegans           --------------------MSDFDIQIKIDDAQLDLKGVSFWVTASVVTLFLAWSTFVV 40
                                        :  :           : ..  .*: .: :     *  ::.

H.sapiens           YYNSRNVGLILTLVLNRLY---K-HGYIHIGSFSFSV-LSGKVMVREIYYITEDMSIRIQ 100
D.melanogaster      FYNSRVIGMLITKIANRWF---IKGAYFKIGSVALNP-LAGKIMFRDFVYITYDYTVRAQ 115
C.elegans           LFFSRVSALFFTFVIDKYLRLSKNGIHFKIGGISISGLHAGKIMFRNVIYDNGDMTIKVN 100
                     : **  .:::* : ::         :::**..::.   :**:*.*:. * . * ::: :

H.sapiens           DGFIIFRWWKMYNPKQKQHD-PKAETRLYITVNDFEFHVYNRSDLYGRLQELFGLEPTII 159
D.melanogaster      DGYFIFRWWRSYVPKDVSEDLSHSDTRLSVQLNGYELHIYNRSDLYDTLEKTFGLEPSLL 175
C.elegans           DGHLLFKYWKSVEHRHLNLS-TKRASRLHLVLNGLHVNIYNNLTKYTEIARIRRFDWFFE 159
                    **.::*::*:    :. . .  :  :** : :*. ..::**.   *  : .   ::  :

H.sapiens           PPKKDDDKTRE---------IGRTRTQSKIERVKVKTESQDPTSSWRS---LIPVIKVNV 207
D.melanogaster      IPTDAASNEERNKLKEHHMNLENARQSQRIQ--NVKNSEAMQATTWRD---LIPVIKIDV 230
C.elegans           N-----------------TNMNDARRPQ-----TKPPDTSPPSSVWENMWNLLGIVHIEV 197
                                        :  :*  .     .   .    :: *..   *: :::::*

H.sapiens           STGRLAFGNHYQPQTLCINFDDAFLTYTTKPPSSHLDQFMHIVKGKLENVRVMLVPSPRY 267
D.melanogaster      CSGRFVFGNRLTPTTLSISVEEAHCTYSTKPAVCRLDHFMHFVKAKVENAKVLFCPSPKY 290
C.elegans           SAGCILVGNKFLPYALWTRFENLNSKTSV--TESANDRALLTFEGETENVAVSLIKNEQF 255
                    .:* : .**:  * :*   .::   . :.    .  *: :  .:.: **. * :  . ::

H.sapiens           VG-LQNDEPPRLMGE-GFVVMQSNDVDIYYYMDEPGLVPEETEENIEGEMSSEDCKLQDL 325
D.melanogaster      TG-LI-DEPPRYMGE-GFVVMMSNQMDLYFYMDEPGVVPEHPVQIVL----PNGDVVEPS 343
C.elegans           DFTAKDKDPPRTMGNDGCPLLQSASLEFVYKQDLLGYVTDDEPQS-----------ITLK 304
                          .:*** **: *  :: * .::: :  *  * * :.  :            :

H.sapiens           PPCWGLDIVCGKGTDFNYGPWADRQRDCLWKFFFPPDYQVLKVSEIAQPGRPRQILAFEL 385
D.melanogaster      PPVWGINARCLRGTDFSYGPWADRQRDHLYRYFYPSDWKEAEVTPTPQPGELRSYQSFDV 403
C.elegans           LPLWSSEWRFGNNTVLSYGPWAEQQRFLIYSFFYPPDFQNSTATAMPTRGKKRIHVKHDV 364
                     * *. :    ..* :.*****::**  :: :*:* *::   .:    *. *      .::

H.sapiens           RMNIIADATIDLLFTKNRETNAVHVNVGAGSYLEINIPMTVEENGYTPAIKGQLLHVDAT 445
D.melanogaster      TLCVLNEATIDILFSKEKETNAMHITVGPASYVEMTIPWVTQPDGYTSKIQGQLFHVEAT 463
C.elegans           KIILTKETCMDIWFMRGEQLESIRTRCGPLSSLDMSILWITTEKGFYWNMKAEFLNFEAT 424
                     : :  :: :*: * : .: ::::    *  * :::.*   .  .*:   ::.::::.:**

H.sapiens           TSMQYRTLLEAEMLAFHINASYPRIWNMPQTWQCELEVYKATYHFIFAQKNFFTDLIQDW 505
D.melanogaster      TSLQYRSLAEFESLEYKVRIHYPTKWNAPQDWSISLSGCKTSAFIVYKHKCFFQDLIEDW 523
C.elegans           TSLIFTKLFSCKKFNVDGSFVYPLTWNGEQTWTIDYAFTKANAWFVWDHKRLFTDLINDW 484
                    **: : .* . : :  .    **  **  * *  .    *:.  ::: :* :* ***:**

H.sapiens           SSDSPPDIFSFVPYTWNFKI-MFHQFEMIWAANQHNWIDCSTKQQENVYLAACGETLNID 564
D.melanogaster      ANKARPDILSFVPYTCNFSI-RLHEFEILMLCNEYNWIDCSSANQENNHLAFCGDVFEMS 582
C.elegans           IGDDPSDISKFVPFRVHNRMKVVDGFEVIMLLNESNWVDTADMNAENVEVAIVGEKLSFE 544
                     ..   ** .***:  :  :  .. **::   *: **:* :  : **  :*  *: :.:.

H.sapiens           FSLPFTDFVPATCNTKFSLRGEDVD-LHLFLPDCHPSKYSLFMLVKNCHPNKMIHDTGIP 623
D.melanogaster      FALPFDDFLPKTVTLKFWIHGEGLD-LSLYVPEVSSVRPIVLAIDENAR---LLTREG-- 636
C.elegans           CELPFVDFLPQTQMVKYEMRGEKSVAMRAKFPPDSATAPIRAALSRLAR----------- 593
                      *** **:* *   *: ::**    :   .*           : . .:

H.sapiens           AECQSGQKTVKPKWRNVTQEKSGWVECWTVPSVMLTIDYTWHPIYPQKADEQLKQSLSEM 683
D.melanogaster      -KLIRRPELYSKKWRKICQRSAGWIDCWAVPILALSIQYVYHPVPPLGPDPQADITTPEK 695
C.elegans           --CNSY--APPSKHGTHSLDTDVWFELWRTELVKMDFDHHYRPLIVKSNIPS-D------ 642
```

```
                                *  .    .  *.: * .  : : ::: ::*:       . .

H.sapiens           EETMLSVLRPSQKTSDRVVSS-PSTSSRPPIDPSELPPDKLHVEMELSPDSQITLYGPLL 742
D.melanogaster      EEILLSPMRIPKVRKSPVSSWQQPPEQYSKFDPGTLAADHVTVELEIGS-SVLMAYGNVL 754
C.elegans           ------------IPFSILSDYLPPP----ANHPWDLEPDYLGVDILIEG-SDVKFTGLLV 685
                                   . : .           .*  *  * : *:: :   * :   * ::

H.sapiens           NAFLCIKENYFGEDDMYMDFEEVISSP---VLSLSTSSSSGWTA------VGMENDKKEN 793
D.melanogaster      RNFISLKENIFGEDQNFTDMEQSNVNMKEPGVAQVNPK-DQLLAKEKELANKSISETQPP 813
C.elegans           KLLFELKNNYFGWYDSMTSVDDEKIDD--PI-KLK-----------------ASFD-KTN 724
                    . :: :*:* **  :   ..::   .                             : :

H.sapiens           EGSAKSIHPLALRPWDITVLVNLYKVHGRLPV-----HGTTDGPECPTAFLERLCFEMKK 848
D.melanogaster      EEKRKPFDPRLYRPLEVMVSVIVHDIQAHVMK-----NCNEDDPPCPVVLIERFGFEMNK 868
C.elegans           ANGMKPV--EYFRTMNVDVTVRVCNVRAEMLLYSPAIDEGAEPEKVPVVFVEEVAVEVKK 782
                        * .     *  :: * * : .::..:       .   :    *..::*.. .*::*

H.sapiens           GFRETMLQLILSPLNVFVSDNYQQRPPVDEVLREGHINLSGLQLRAHAMFSAEGLPLGSD 908
D.melanogaster      KYHETTLQVLVSPSYLLTSDCL-QRSQREQHINQGHLMLSAVQVRGHAMFSNEGCALDED 927
C.elegans           TKTQALIQVGVSPACAYLDKSS-------QGSGPGCITLSGFQFRGHAMYSAKEVAWNMG 835
                       :: :*: :**     ..         :    * : **..*.*.***:* :    . .

H.sapiens           SLEYAWLIDVQAGSLTAKVTAPQLACLLEW-GQTFVFHVVCREYELERPKSVIICQHGID 967
D.melanogaster      TLEYSWLVEVQLGKLTGKLTLPQLVNVVTG-LETLILLAIDPENCLKSPKTVRNCHHGVP 986
C.elegans           LVEYGWIMEILVGDIAGTLDFPAHAHVLHQIMESLLMFVISPDDATKVPDRMQFCQHGQL 895
                     :**.*::::  *.::..:  *  . ::    ::::: .:  :   : *. :  *:**

H.sapiens           RRFCE---SKLSCIPGPCPTSDDLKYTMIRLAVDGADIYIVEHGCATNIKMGAIRVANCN 1024
D.melanogaster      SNLCP---QTKEEKKYKCPSSEDIKYKMTRVSVDAVDVYLIESGTALHAWISPIRLANCN 1043
C.elegans           IKACSIAGKKTNEILGPCKTEEQMKYRQIRISVDSVNLTFVEEKTILQISADPVRVTICN 955
                     . *    .. .     * :.:::**   *::**..:: ::*     :   . :*:: **

H.sapiens           LHNQSVGEGISAAIQDFQVRQYIEQLNNCRI----------------------------- 1055
D.melanogaster      LHGQRVKSGISGLLPSILLRLFMLHTTNSTFNTNTTGSNRSGKLRRADQDSLKSQDAGGS 1103
C.elegans           AHESRFTEHVCIRVPGISIRQAVRIK---------------------------------- 981
                     * . . . :.   : .: :*  :

H.sapiens           --------------------------------------GLQPAVLRRAYWLEAGSANLGL 1077
D.melanogaster      HYASHGKTGKRSSNSFSRRDSREEATRKLRGSFSETHTKRTPETEITENWVEVGCTSLGP 1163
C.elegans           -------------------------------------------EKPENIWIEGANAAIEG 998
                                                                 *:* . : :

H.sapiens           ITVDI--ALAADHH---SKHEAQRHFLETHDARTKRLWFLWPDDI--LKNKRCRNKCGCL 1130
D.melanogaster      ILLEGASALPIPDH---ELHLVQHNFLREHDAKFKRLWFLWSNNGSALSSGSEISRCGCI 1220
C.elegans           VSLDI--ELPTPKSASPTIGKERLEFVRMHDADTKRLHFLWADHS--------VWGCACF 1048
                    : ::    *   .         : .*:. ***  *** *** :.            *.*:

H.sapiens           GGCRFFGGTVTGL-DFFKLEELTPSSSS---AFSSTSAESDMYYGQSLLQPGEWIITKEI 1186
D.melanogaster      GGCAFFGSNRNGQ-KFFKPTAQDAHDNYNIARYFIINNNKDFGFGESILHQGQLVFHTPP 1279
C.elegans           GNTCFFGDVDEIGSTFMETLT--------KKKFFVPGIERNPEKQPQVM-QSVILKNKPI 1099
                    *.  ***.       *::              :   . : :     .::  .  :  .

H.sapiens           PKII------DGNVNGMKRKEWENKSVGIEVERKTQHLSLQV--PLRSHSSSSS----SE 1234
D.melanogaster      YSLHCVSLYDTADFNGKGRLYRPAGDLRNGSLKKTDLCSLPDGTKFKIGASGTTGVEKPE 1339
C.elegans           LS---------------------------------------------------------- 1101
                     .

H.sapiens           EN----SSSSAAQP--LLAGEKESPSS------VADDHLV-----QKEFLHGTKR-D--- 1273
D.melanogaster      PNCRNKSRESIGSPNTLERRTKRYPCTRQTSVDVPYARLLDSPSKKLQLQHEASAGDAGS 1399
C.elegans           -----------NQPHMFYKKPKNA--------DVV----I--------TIRKESTGDT-- 1128
                                .*  :    *.          *     :          :  .  *

H.sapiens           ------------------------------DGQASIPTEISGNSPVSPNTQDKSVGQSPL 1303
```

```
D.melanogaster      SHRRGSDSNRLRVSPPKTSISDSRLTGDVLDDETEIPDEMSHSAPH-------------- 1445
C.elegans           ------------------------------------------------------------ 1128


H.sapiens           RSPLKRQASVCSTRLGSTKSLTAAFYGDKQPVTVGVQFSSDVSRSDENVLDSPKQRRSFG 1363
D.melanogaster      SHPLEFEIA-------DIALHVQGLGGDQLPREVQRTI---------------------- 1476
C.elegans           ------------------------------------------------------------ 1128


H.sapiens           SFPYTPSADSNSFHQYRSMDSSMSMADSEAYFSAAEEFEPISSDEGPG-----------T 1412
D.melanogaster      --------------------SLTSENPSEMFFSAEEDISNVLSQRGSMKQRNSVNSGSVV 1516
C.elegans           ----------------------------ESFHSARS-------QQSPGL----------- 1142
                                                * :.** .       :..

H.sapiens           YPGRKKKKK-----------QTQQIDYSRGSIYHSVEGPLTG-----HGESIQDSRTLP- 1455
D.melanogaster      LSGKKRFSSDLSIGAQNDNGSHTLPTYRSDLEIHAPDGNKTLPKRPQSTTELADSRGSSS 1576
C.elegans           ----RI---------------------------------------------------LQS 1147
                        :

H.sapiens           FKTHPSQASFVSALGGEDDVIEHLYIVEGEKTVESEQITPQQPVMNCYQTYLTQFQVINW 1515
D.melanogaster      GTPSLSSNSFISAMSSQEDVALV----NLHQQV-NRPIIDSPLLMASYLNHLSQVKCFNW 1631
C.elegans           MEMSSSYATFVDNVRV-----------ELPSAITV----P---------QFGEPGAILEW 1183
                         *  :*:. :             :  . :                 .      ::*

H.sapiens           SVKHPTNKRTSKSSLHRPLDLDTPTSEESSSSFEQLSVPTFKVIKQG--------LTANS 1567
D.melanogaster      NGCSFPLGP---DVFSTPLFSENE--DGGLTYIGSKMLPHFDLYSCWREIKVVPRYENAT 1686
C.elegans           CQAHQATRI----------INDV-----------------------------------NTS 1199
                                         :                                     :

H.sapiens           LLDRGMQLSGSTSNTPYTPLEKKLA---DNTDDETLTEE-WTLDQPVSQTRTTAIVEVKG 1623
D.melanogaster      GSNSSATFMGGPKSHPWDPSVLLKEEESDKTTNGFDDGEFMSLQAEGGAVCTSVVARLKG 1746
C.elegans           GVNE-VRFLSKPKKS-------------------QDIEYNTSRDTLGKRRLAINGVAAT 1238
                      :    : .  ..                        *  :     .    :

H.sapiens           TVDIVLTPLVAEALDRYIEAMVHCASTRHPAAIVDDLHAKVLREAVQNSKTTFSENLSSK 1683
D.melanogaster      QLNVFLTPLLLEGLQRMVEAAVPTIQSMHLLSVVNFIHTSCIAKVNNDNILKRDQSLSYW 1806
C.elegans           SLDLFVTPIGIEAFERLVTAASHSVPAINPCILVHMCYRDCVLKKHRQPLT---ESL--- 1292
                     :::.:**:  *.::* : *      : :   :*.  : . : :  .:      :.*

H.sapiens           QDIRGTKTEQSTIGTTNQGQAQTNLTMKQDNVTIKGLQTNVSIPKVNLCLLQASVEESPT 1743
D.melanogaster      SQVHS-NSKRSTTERHLQGPGDSFLSDVYEESISTKTQGLIVLPKVSITMLQSSIVEEII 1865
C.elegans           ----------------------FADEDNDSEPISEVDITVDLPRVSIGLFQCGVKKNIV 1329
                                             :.   .  :  : :*:*.: ::*..: :.

H.sapiens           TA----PSRSVTHVSLVALCFDRIATQVRMNRGVVEET------------SNNAEPGRTS 1787
D.melanogaster      SVAALDNVQDLSCVSLLTFYMEGISTKFHMGKTTRASMHNVYIQQTVQSGSSNKKGGIMK 1925
C.elegans           KSNHTDHITANMGLL----LIDRA-----------------FIQSKLIPAE-----SVSQ 1363
                    .            :      ::                            .     .  .

H.sapiens           NFDRYV----HATKMQPQSSGSLRSNAGAEKGKEIAAKLNIHRVHGQLRGLDTTDI---- 1839
D.melanogaster      GTRALLAHLSSQTRPDNVQGEPILIETSEKQLEEVVITLDIGRAHAQLRRLKTEGQSCTQ 1985
C.elegans           DFSADTSN-----------------------LSSTLYQLNGSAITVQLLQLTNRDA---- 1396
                    .                               ..    *:      **  * . .

H.sapiens           -GTCAITAIPFEKSKVLFTLEELDEFTFVDETDQQAVPDVTRIGPSQ--EKWGW------ 1890
D.melanogaster      DSPIIVTAIPEHKSKVLFECLKMPE------------------STGI--ESIGY------ 2019
C.elegans           -------------------------------------PDFGSSGTATTPNNWEHCAISRR 1419
                                                               . .   :.

H.sapiens           -------IMFECGLENLTIKGGRQSGAVLY--------NSFGIMGKASDTERGGVLTSNN 1935
D.melanogaster      -------IMFECGLEGVGVKIVKRSHFEKS-ENSKEELAEMAGAGAGGGASAGGFNLNDL 2071
C.elegans           MNNLEPRVMMDFNVSDTLIIILERRPIILLLPDKSTTAITPIH--SPA------------ 1464
                           :*:: .:..  :   ::                :   . .
```

```
H.sapiens           --SSDSPTGSGYNTDVS--------------DDNLPCDRTSPSSDLN----------GNS 1969
D.melanogaster      VGQGEGAAGSGDGGATSKAEAAWRLITKKPPTPKTPKEKFQPASDSNISAETSGAEKGKS 2131
C.elegans           ----NAPTPT----A----------------MNRTPTLT--------------------- 1479
                        :. : :                       . *

H.sapiens           VSDEQDEG-----------------------VESDDLKKDLPLMPPPPDSCSMKLTIKE- 2005
D.melanogaster      TPKPPDEDVEKGTSPANAQTGAQKPSAGAGTNTKDNYDKVLSNVKETDKTSSCVIELKA- 2190
C.elegans           ----------------------LTPSAGAGGGERAEP-----MRKKKK--MICEHYLKAD 1510
                                                       :                    :*

H.sapiens           -----IWFSFAAPTNVRSHT--HAFSRQLNLLSTATPAVGAWLVPIDQLKSSLNKLETEG 2058
D.melanogaster      -----VWFNFAAPPCVPITRKIDLTRLDWNLLSTASPAITAWMNPSNRLAMKIVSLMKAL 2245
C.elegans           IGSVTTALVMARPQEL-------TAGDEFPIYEALAPVMVSWLSVVENFLRTVDKFIHTV 1563
                           : :* *  :           :  : .: :*.: :*:   :.:  .: .:

H.sapiens           TLRICAVMGCIMTEALENKSVHFPLRSK----YNRLTKVARFLQENPSCLLCNILHHYLH 2114
D.melanogaster      HTRQTAVAACLMAEAMDNEKIQRNPKIKKSRYANNYTLLSKTLQEDPSCQLCYIMQKLVL 2305
C.elegans           ECWKSVAMAKVLKLALDSTDEKVVVKVGKNRM-GRTRVLSA---HQASCPSCILLKTLFR 1619
                         .. . ::  *::. . :   :       ..   ::    .: **  * :::  .

H.sapiens           QANYSIIDDAT------MSDGLPALVTLKKGLVALARQWMKFIVVTPA---FKGVSLHRP 2165
D.melanogaster      DEGVQRIETIF------KQHDVPHLNTLRQGIIVLSRQWKNTLYNPIL---FEHQYKNKL 2356
C.elegans           WFAYAGNAPGAINHRLDIRPEFEIEETRKTALMALLSHWQSDVGKELKLVSYEDAHRFKV 1679
                                         .    * : .::.*  :* . :        ::     :

H.sapiens           AQPLKPQIAMDHEHEDGLGLDNGGGLQSDTSADGAEFEFDAATVSEHTMLLEGTANRPP- 2224
D.melanogaster      SRPINVTFSFPQNEEDAENDECEGDVEMGA--------------------FAGVGENPEE 2396
C.elegans           TRPDEAAIVALTK---------SKRLKR------KMLEKKESSKKETRVVMEVKPEQPK- 1723
                    ::* :  :    :            ::                       :    :.*

H.sapiens           ---PGSSGPVTGAEIMRKLSKTHTHSDSALKIKGIHPYHSLSYTSGDTATDSPVHVGRAG 2281
D.melanogaster      SATYGHEGANHGTASQRS-----------------------------TSTSPNIHHPRRG 2427
C.elegans           ----AKRGRMSPAPLLKKLRKK----------------------AGDDDFD--------- 1748
                        .  *    :   :.                                .

H.sapiens           MPVKDSPRKESLLSYLTG-SFPSLHNLLEGTPQRSSAAVKSSSLTRTGNTVATDMLS-EH 2339
D.melanogaster      ---------IQMLPIISG-QVPEFEY----------GALQEGSL------SSTNSVN-KS 2460
C.elegans           ---------DDSMKFLSDVEMQEFNTLP-----------------LYEDYEDDEMLENLD 1782
                              . :  ::. .. .:.                            : :.

H.sapiens           PLLSEPSSVSFYNWMSNAVGNRGSVLQESPVTKSGHNSLPTGVAPNLPTIPSASDFNTVL 2399
D.melanogaster      WLGTENHKEDLYFWMAKQQDNKKKHFTEKEHAR-----------PAPPKM---------- 2499
C.elegans           SEPKIDDKVDLYTWMRNAQRESTL--RRRKLAGGAEGSVKDDL----------------- 1823
                       .   . .:* ** :   :      .   :

H.sapiens           SSDQNTLDGTHSQHSTSQDDVAGVEEANQGFPAVQLADAQVVFKPLLSHTGIQSQDT--- 2456
D.melanogaster      ------------------TEHAGQTRSGIMQDSIKLLDAHLIFEPLLTCLGVMPQQM--- 2538
C.elegans           -----------------------------------------NLKGYINPMDIQQKAYYYN 1842
                                                              ::  :.  .:  :

H.sapiens           MP------FCYR---MYFGEHLSFSGTLDCLRADIVDSDTAKERKGKRARRQGHVNLP-- 2505
D.melanogaster      INKFSNADISSL---ENFGTNLSLIGTFDSIRVDIVVSEAGDKKNSAQK--PAKLNKKSN 2593
C.elegans           IYRWAQLQWTSLDGIEKDHWHLDYSVTLREVDVRMMAKSI---KNSSD-----HLRQYIT 1894
                    :                   :*.   *:  : . :: ..    ::.       ::.

H.sapiens           ----PLEFK-PALMLGTFSISAVV--MEKSVCTPQNSTSALSFHDLSKRYYNTFHCNFTI 2558
D.melanogaster      GGRASIMMDTPLFLCERVGVELEVLKMSDGMVD-QARQNVIYMSRRQLKKHTSTVINFSL 2652
C.elegans           PAQQKVMQVRNAAVN-----GGMVWKMER-------------DERRKIPLHGQWNISYSG 1936
                         :       :         *  *.                  .   :     .::

H.sapiens           SCQSISQHVDMALVRLIHQFSTMIDDIKATQTDIKLSRYTAGS----------ASPT--P 2606
D.melanogaster      NVRFISQQVNMPLLRLLHQICNMYQNVKDAQNEFHDQPELSKKSQTKDECSLASEPTDIV 2712
```

```
C.elegans           NVEGIRFLIGMATVSLGKELSLVLRVAMEAKNELRMHSTAESFQ---------TPRNEVK 1987
                    . . *   :.*  : * :::. :      ::.:::                  :  .

H.sapiens           TFKTR-KHR-----DFR---S---SDF--------SRSSRGSLNGGNRVN---NAKNKRT 2643
D.melanogaster      PFNSM-SERYNHAENYSDERY---DKFNETMPTMLARPRPGGLAPIIQLTPSPNAKNRPQ 2768
C.elegans           VFKPVVPNQYDLAVEWDEKVLDMTRDYEKHM----------------------------- 2018
                     *:    .:     ::         .:

H.sapiens           NNENNKKESR---NKNSLGRSERRT---SKVSRKGSKDVVDHMTIHMDD----------- 2686
D.melanogaster      SF-AQKLRSTGKSVKGKLGYTNLNESSSSPLRDSPTMSLHEHNILKMSTESKASLNGACA 2827
C.elegans           ----QRMRT------------------------NKEDVVEKVKV--------MVNGSAM 2041
                        :: .:                           . .: ::  :

H.sapiens           ------------SDSITVSEQSEPSAECWQNMYKLLN--------FYSLISDPTGILEKS 2726
D.melanogaster      TSVSGDYQNTLTKAGMPTMAPMLETPNCWKTIYHLLE--------LYGTMPETKTVVQRS 2879
C.elegans           V-----------------S--SIVLESVLNDLYVSVTISQIVLAHSKNPMPDIPVVVHAV 2082
                                          .  : :*  :           . : :  ::.

H.sapiens           SETFGPAGVRSPTEPTCKVVFENEQDN--S---SLTKTQRKRSLVTSEPQHVTLIVFGIG 2781
D.melanogaster      SLNEHKSK---------SAGFAHDDDDLASAPTPLPQHREMLLVDASSQERTRLIVFGVA 2930
C.elegans           TLSTTPTA---------AA---AEDKKTA----TLTKK----SV---------SSTFKID 2113
                    : .   :           .    ::..       * :      :           .* :

H.sapiens           MVNRTHLEADIGGLTMESELKRIHGSFTLKEKMKDVLHQKMTETCATAHIGGVNIVLLEG 2841
D.melanogaster      KIHKTRLLATLSGLKLESEITTLNSTATWRKKARPVS----LECSLTGQVGRAMIVLLEG 2986
C.elegans           D-----LTVSLTKMKL--T---------LSEADSSNKKSDILRCTLNSSSFNVHT----- 2152
                          * . :  :.:              :           .   ..    .

H.sapiens           ITPNIQLEDFPTSPTSTAKQEFLTVVKCSIAKSQALYSAQR-GLKTNNAAVFKVGAISIN 2900
D.melanogaster      VAPSQQ-----------------TVVKVTVGKSQTLYSSLSKRGKDKNSGLLSIGAVNID 3029
C.elegans           -----NLKT-------------LTSAKESNRPKNNL-----INSNIATTATLRLGALEGT 2189
                         :                 * .* :   .: *        :  .:. : :**:.

H.sapiens           IPQHPATLHSMMVRSSHQLSKQISDLIRQPSTAPQPVKED----IATPLPS--------- 2947
D.melanogaster      IPQHPVALHGMMTRSSKQLSSTLQELRVKRNSGRSTMRSHTAEEPESPFHARNSVGASSG 3089
C.elegans           MPMAAYSLHDVVMRHGKELEQQLNRLAAQPASTPLSS--------STPFPSAEQSLLAKV 2241
                    :*    :**.:: * .::*.. :. *  :  :              :*: :

H.sapiens           -----EKTPTS------VNQTPVE----TNEFPQL------PEGLEKKPIVLKFSAMLDG 2986
D.melanogaster      TGVASEMRERTVSGGAGAQQQPQQAARRAAHMAQSKTGTAQHQNGLLQPLVMQFNVLLQS 3149
C.elegans           ADMKTAPEPVVIT---QAEFKPLTTLPATAAHVQDAKG---QIVRRVPVAVVSFSIELTS 2295
                                     .:  *      :    *                *:.*.  * .

H.sapiens           IAIGAALLPSLKAEYKMGRMRSHGMTGAQTRFTFELPNHRLRFTSKVSATDMSTIPPSAS 3046
D.melanogaster      LSINAALLPSLQAQYRMNHVSSMGVTGQRAKFVIDLPTHTLSFNTKIQN--EMNLPSEAC 3207
C.elegans           IEMNIQLLPSLQAKYRINRATSNGITGVQANWSILLDEHFFEFCVTGQGGKT----ETFR 2351
                    : :.  *****:*:*::.:  * *:** ::.: : *  * : *  . .

H.sapiens           LNLPPVTMSGKYIMEEHDSYSDQVWSIDELPSKQGYYLQGNYLRCVAEVGSFEHNLTTDL 3106
D.melanogaster      IGLPPVHVLAEYIPDHRQDHTENVEG--------IVLRQGGYVNASAEIGEFERCLTTDL 3259
C.elegans           LQLPSVTSDGLYQAEQGV------SSQKPSTDKKLIYREGGSLQMTVVLGRVNHIFTTEL 2405
                    : ** *   . *  :.         .            :*. :.  . :* .:: :**:*

H.sapiens           LNHLVFVQKVFMKEVNEVIQKVSGGEQPIPLWNEHDGTADGD------------------ 3148
D.melanogaster      LNHLVFVQKVFMREINEVLQKVYGGEKPVPLWTEESGDSSGA------------------ 3301
C.elegans           LNQLMFAEHSFRTELTALINRIRSSSFASTNSSRSAQSTNDRVNSTANLKLLLPVQTTST 2465
                    **:*:*.:: *  *:. ::::: ...      ..    :..

H.sapiens           ----KPKILLYSLNLQF-----------------------------------------KG 3163
D.melanogaster      VQESSLKRILFSINISM-----------------------------------------KR 3320
C.elegans           PVHIEKPPLLFSIKIQTKEIPRKDEKTDKDAKTPKASGASGNLDQSQHPSHSTHVKSTPW 2525
                        .   :*:*:::.
```

```
H.sapiens          IQVTATTPSMRAVRFETGLIELELSNRLQTKASPGSSSYLKLFGKCQVDLNLALGQIVKH 3223
D.melanogaster     IQLTATTPCSSAVRFETGILELQLSNRVKNL---GDMSNRKLFFKAHIDFNLSLGQIIRN 3377
C.elegans          LQLTAATPTQTAVRLTVDSLEGELTNKWVVK---EEGSKERIYGNAVIHFNAKLGQLIKP 2582
                   :*:**:**   ***: .. :* :*:*:         . *  ::: :. :.:*  ***:::

H.sapiens          QVYEE-----AGSDFHQVAYFKTRIGLRNALREEISGSSDREAVLITLNRPIVYAQPVAF 3278
D.melanogaster     VIFDE-----AEPEFQQYAFFHTTINLRNAFQDELLN-EDKELILLTLKRPLVYVQPIAV 3431
C.elegans          VPTGDSVAATDVTDLQEFATFMTQVRVENKERNMFNS---SYSYHISLNRPIFLVKAAAI 2639
                       :        :::: * * * : :.*  :: : .        ::*:**:. .:  *.

H.sapiens          DRAVLFWLNYKAAYDNWNEQRMALHKDIHM----------------ATKEVVDMLPGIQQ 3322
D.melanogaster     DKAILVWLNYKNAYEYWAEKRANLCHEHAQHSLLSQYSGQHQNQNMQNPQVFDR-VAFGQ 3490
C.elegans          DKAILLWLNYKNTYDYWRNEREKVVQEKTTTKLSN--------AGMFSP---------TQ 2682
                   *:*:*.***** :*: * ::*  : ::                     .           *

H.sapiens          TSAQAFGTLFLQ-LTVNDLGICLPITNTAQ----SNHTGDLDTGSALVLTIESTLITACS 3377
D.melanogaster     IAGSNLSTLFLQ-LTVEDMGICLPLKQVNTTTFGSRSYQDFDAKGAVVITLENTIISACN 3549
C.elegans          IAEDA--DMNLSLAINNGMYMCMPLYSHDV----------TEGMPALVLSLQKSNLSVLV 2730
                    : .    : *.    :.: :*:*: .               :   *:*::::.: ::.

H.sapiens          SESLVSKGHFKNFCIRFADGFETSWDDWK-PEIHGDLVMNACVVPDGTYEVCSRTTGQAA 3436
D.melanogaster     SGALVSKGKFQGLCLRFADDFETNLDDWK-PNSA-EPIMNVCVVSEGTFEVCSRTTAAK- 3606
C.elegans          KKELTCKASFNGFKCSFVDDFDEQALTQSFLDATHSDQSNCIFFPEGTYQLCSKAEATK- 2789
                   .  *..*. *:.:   *.*.*: .     .  :   .   *  .. :**:::**:: .

H.sapiens          AESSSAGTWTLNVLWKMCGIDVHMDPNIGKRLNALGNTLTTLTGEEDIDDIADLNSVNIA 3496
D.melanogaster     --KGENAKWLLNVKWQMEGVDIHLDVNIGKQLSSLGHTLTMLTGFEEDETQMESPDSDEG 3664
C.elegans          ----GPAKWVLSVSAEMQGVEIDLDTRIGKLAKLLVNTFSMIRTDDDDDMSFWG---DEG 2842
                         ..* *.*  :* *:::.:* .***  . * :*:: :   :: :        : .

H.sapiens          DLSDE-DEVDTMSPTIHTEATDYRRQAASASQPGELRGRKIMKRIVDIRELNEQAKVIDD 3555
D.melanogaster     DQSCR--------DTFVRRRGDFDNLPAFVFDPTI---DSKKRSFMMEKEMAEQLKIIND 3713
C.elegans          ELDSDEEKVE--------GASELKKL------------KAEEKVPWMENKMHEHSRAVFE 2882
                   : .                    :  .                :      .:: *: : : :

H.sapiens          LKKLGASEGTINQEIQRYQQLESVAVNDIRRDVRKKLRRSSMRAASLKDKWGLSYKPSYS 3615
D.melanogaster     LRTLGASHNTVAHEERRLQELQAICYKYFRRDMIQKWKRPSLRRSLKT----------YG 3763
C.elegans          LAARGVSNKLIEAEKHKLRQYELIRFKAFRRNVVEKLKKGTTASRQHTE----------- 2931
                   *   *.*.  :  *  :: :: : :  : :**:: :* :: :       .

H.sapiens          RSKSISASGRPPLKRMERASSRVGETEELPEIRVDAASPGPRVTFNIQDTFPEETELDLL 3675
D.melanogaster     RSHSYIGSGSS--------VSGVGVPQTLDNV----SYTGRRL-----DTIASNDEISSL 3806
C.elegans          ---------TP-------------------------PPQPRP-----DT---------- 2941
                                                         *      **

H.sapiens          SVTIEGPSHYSSNSEGSCSVFS---SPKTPGGFSP-GI----PFQTEEGRRDDSLSSTSE 3727
D.melanogaster     Q---STP--------ASCHSRSASLKHTTGGGVIGGGVNALGRVTFTEAMRQTSLPNADT 3855
C.elegans          ---------------------------------------------TSRRNSRTTS--- 2951
                                                                 . *:    .

H.sapiens          DSEKDEKDEDHERERFYIYRKPSHTSRKKATGFAAVHQLFTERWPTTPVNRSLSGTATER 3787
D.melanogaster     DTETADNELD--------WRGDITPSEIDVDGTSV----------EMRRKHGHGQKQPEP 3897
C.elegans          --TSQKNS-----------------------------------------EDLTTPGDIE 2967
                      . .:.                                          ..

H.sapiens          NIDFELDIRVEIDSGKCVLHPTTLLQEHDDISLRRSYDRSSRSLDQDSPS-------KKK 3840
D.melanogaster     NIDFELDIKVLVNSGKCVLHTKDTGEER-------GYAGGSGAATSGVPASSVKSHKREK 3950
C.elegans          TVNFNLDVKVNITSGTCTLRTQKKEGAN-QL---------------ALPGIL-----KRL 3006
                   .::*:**::* : **.*.*:        .                 *.       :.

H.sapiens          KFQTNYASTTHLMT-GKKVPSSLQTKPSDLETTVFYIPGVDVKLHYNSKTLKTESPN--- 3896
D.melanogaster     SIGNDWGSPTPSRRQRDKSKLRYNANALLADLTIFHIPGLDVKLHYQSKTLAEQLTG--- 4007
C.elegans          NLG--------------TKDIKAMFEPQIITTTTFSIPSVEIKAYHVSDPSNRSTDEFCK 3052
```

```
                    .:               .       :      * * **.:::* :: *.    .

H.sapiens           ----------ASRGSSLPRTLSKESKLYGMKD-SATSPPSPPLPSTVQSKTNTLLPPQPP 3945
D.melanogaster      --------------DQL------------------------------------------- 4010
C.elegans           DKREKISKGLAKDADKLHRDLHNKSRFGNTYINGGGGP-----------KTST-V----- 3095
                                  ..*

H.sapiens           PIPAAKGKGSGGVKTAKLYAWVALQSLPEEMVISPCLLDFLEKALETIPITPVERNYTAV 4005
D.melanogaster      --PT---GRRMGSKRATLCAWMTLQSIPEETIISPHILEFLEQTLEPIPARQSSSVPP-- 4063
C.elegans           ---------PPPPKRGCFYIFVGLASMPSETVVTPHLATYFEQVLEPLPPSAVFQSQNN- 3145
                                 * . :  :: * *:*.* :::* :  ::*:.** :*

H.sapiens           SSQDEDMGHFEIPDPMEE-STTSLVSSSTSAYSSFPVDVVVYVRVQPSQIKFSCLP---- 4060
D.melanogaster      -----------TPSHNTG-VNLDILPANYVTYASFPVDVIVYFHMQPSTFRFSCLP---- 4107
C.elegans           ------TREASVPDDGKGDANNEVHNIMAMDTAAFPIDFVFYLDVQSSTIRFDGKQPTSR 3199
                                *.      . .:        ::**:*.:.*. :* * ::*.

H.sapiens           -VSRVECMLKLPSLDLVFSSNRGELETLGTTYPAETLSPGGNATQSGTKTSASKTGIPGS 4119
D.melanogaster      -VSRVECMLQLPSLDIVFSSKRSSEEENS-------AQPGGHTQ---------------- 4143
C.elegans           SQTQADCLLTLPRLTLELTSKRTRDNID-------------------------------- 3227
                      ::.:*:* ** * : ::*:*   :

H.sapiens           SGLGSPLGRSRHSSSQSDLTSSSSSSSSGLSFTACMSDFSLYVFHPYGAGKQKTAVSGLTP 4179
D.melanogaster      -------------------PDQQLPTGGLSVTGCLADFNVFIFHPYGGKKTSKE------ 4178
C.elegans           ----------------------NYVGGIHISGQFKGFMLKIYNPLELEPDS-------- 3256
                                          .*: .:. : .* : :::*      .

H.sapiens           GSGGLGNVDEEPTSVTGRKDSLSINLEFVKVSLSRIRRSGGASFFESQSVSKSASKMDTT 4239
D.melanogaster      -------TQFSPLSDSERKDSLSINVEFVKFHITRCRKV----YIEPLPSSKRSLDQSRA 4227
C.elegans           ------------------SRALQLSLDLLSFVISRNKNS---------------STEPDN 3283
                                      . :*.:.::::.. ::* :.

H.sapiens           LINISAVCDIGSASFKYDMRRLSEILAFPRAWYRRSIARRLFLGDQTINLPTSG---PG- 4295
D.melanogaster      VIRFSTIVDIGSASFKYDMRRLTEILAFPKAWYRRRIVRRLFLGDLSVQQQQQQQQGNGA 4287
C.elegans           RVRFVFSSQISKASFEYNFRRLGELIQFPKPWYRAAIARRVFFGDQAAPRQKDDASDITG 3343
                     :.:    :*..***:*::*** *:: **: ***  *.**:*:** :     .

H.sapiens           -TPDSIEGVSQHLSPESSRKAYCKTWEQPSQSASFTHMPQSPNVFNEHMTNSTMSPGTVG 4354
D.melanogaster      ETPTGCPPATPTPNEDASRAK-------DNMRLDFDGQPSQ----------QQQQLGHFG 4330
C.elegans           TTRS-------------------------------RLPTDPK------------------ 3354
                     *                                    * .

H.sapiens           QSLKSPASIRSRSVSDSSVPRRDSLSKTST-PFNKSNKAASQQGTPWETLVVFAINLKQL 4413
D.melanogaster      A-------VRH----------LKNLGKSSSAESSGTPPSEKNQITAWETLVIFAVNFTKL 4373
C.elegans           -SLQPP---------------------AASTASTGSGSFVPHQRKPWTALVLAAIQWNEF 3392
                                               :::   . :     :* . * :**: *:: .::

H.sapiens           NVQMNMSNVMGNTTWTTSGLKSQGRLSVGSNRDREISMSVGLGRSQLDSKGGVVGGTIDV 4473
D.melanogaster      NVQMNIGNVCGNVVWLTKDFQSDGRLSIGSTGYKNMYAGIYLGGSALDAKGGIVGGSFEV 4433
C.elegans           EVTAFMSNTMGKTTWKATKGLVWGDAKLNSLNERDVSISFVLGSSELCARDGAISGTIML 3452
                    :*   :.*. *:..* :.     *  .:.*   :::  .. ** * * ::.* :.*:: :

H.sapiens           NALEMVAHI--SEHPNQQPSHKIQITMGSTEARVDYMGSSILMGIFSNADLKLQDEWKVN 4531
D.melanogaster      NKINKRFHI--KEEAGMEPYHTMGLSFMALELRLDYMGTSVLMTRISSFSAAMKDEWRTA 4491
C.elegans           NNLKVSADHSLSADVKRVPVNKAKIRLEWITANIEWMSRRVLIAKWCGPSFKVNDYYKGL 3512
                    * ::   .   . .    * :.  : :     .:::*.  :*:   .. .  ::* ::

H.sapiens           LYNTLD---SSITDKSEIFVHGDLKWDIFQVMISRSTTPDLIKIGMKLQEFFTQQFDTSK 4588
D.melanogaster      SQAAATPAHGKDQPRALIFIHGDLTWDQLQIMISKSTTADLLKMYFKLEEFFTQQFKSSK 4551
C.elegans           KE-------GDHFALSELGMNVQASWKDLQVVITKSTVDDVAAIVNRLISFIDEQLKNSR 3565
                             ..    : : :: : .*. :*::*::**. *:  :  :* .*: :*:..*:

H.sapiens           RALSTWGPVPYLPPKTMTSN----------------LEKSSQEQLLDAAHHRHWPGVLKV 4632
```

```
D.melanogaster      RVFSNLEPRLQDRTASIKRRQQHKKKPANGELVAPPQIHGMIGENTDARHHRHWQKPLAQ 4611
C.elegans           ILLGNLSASTNLKKQAQ----------------------ALIE--SRKPTTHFWEKVLDY 3601
                      :..          :                       .           :.*   *

H.sapiens           VSGCHISL-FQIPLPEDGMQFGGSMSLHGNHMTLACFHGPNFRSKSWALFHLEEPNIAFW 4691
D.melanogaster      AVGLVVPS-LVTRLPRHGNVLGGTVELRGQNISLACFHGINFKSKSWALFSLRSPSINFA 4670
C.elegans           MSEMQMNEQLMGLMEREGAKVGGHIELKAGGISLVMMKG-DMNADTWAVFHLRDACILFD 3660
                         :   :   : ..*  .** :.*:.  ::*. ::* ::.:.:**:* *..  * *

H.sapiens           TEAQKIWEDGSSDHSTY--IVQTLDFHLGHNTMVTKPCGALESPMATITKITRRRHENPP 4749
D.melanogaster      TEARQSE------DEVL--VTQTLTSSLGQTTEVQQQQ---NHSMAIVSRITRN--IIFP 4717
C.elegans           PEARMDFLDNSSQQKIGILLKQTFCLQLGSRHGNQTENR--ANVCRVQTRFNNSRHLQK- 3717
                     **:         ..    : **:   **                   :::..

H.sapiens           HGVASVKEWFNYVTA----TRNEEL-------NLLRN-VDAN-NTEN-----STTVKNSS 4791
D.melanogaster      PQFKTLNEWFHYAFA----NSEIDAVDRFPMLECERE-IASN-SIER-----T----RAS 4762
C.elegans           --AEDILEFFIGDVMKIIGSADHSEKKKLKEVEVIQSPISENENTAKSPTSTFSRFRSPG 3775
                         : *:*         . : .        :  :. :  * .  .            .

H.sapiens           LLSGFRGGSSYNHETETIFALPRMQLDFKSIHVQEPQEPSLQDAS--LKPKVECSVVTEF 4849
D.melanogaster      GSSSAAKAQEHNHNREVIFALPSLQLHFKTEHKQGPTTPEP--SE--NKPEVLCSFITEF 4818
C.elegans           TSKTKESGPATNHNVMELFQFPGLEAKMSSQQLNGVDDGDKYESVFQMPMDVLTTFVCDF 3835
                      .    .   **:   :* :* :: .:.: : :     .   :      .*  :.: :*

H.sapiens           TDHICVTM-DAELIMFLHDLVSAYLKEKEKAIFPPRILSTRP------------------ 4890
D.melanogaster      DDHIFVTV-DADAFFFLHDLITSYVTEKEKVIGAQSARAASPNLSQKANLKPYLTDEILK 4877
C.elegans           FSEVAIETNFNAQVSFLPELLKSYLKESHSGTSSSHS----------------------- 3872
                     ..: :       . ** :*:.:*:.*...

H.sapiens           ---GQKSPIII------------------------------------------------- 4898
D.melanogaster      EKKGASNTNLTAQGKQTSGSKNSLDPLQGSHTSLANAAANMSGAATTNTTTTTATTLSP 4937
C.elegans           ------------------------------------------------------------ 3872


H.sapiens           ----------HDDNSSD-K-------DREDSITYTTVDWRDFMCNTWHLEPTLRLISWTG 4940
D.melanogaster      GAAAGGPSTSATNDSVDGKQQQQEGSPPTFDLESFVRDWRHFECQTWHLEPTVRLLSWAG 4997
C.elegans           ------------TNSSP-----AVSSSKESVVSETSKDPRIFTCQEWKVEPRVRFIDRI- 3914
                                 :*                :     * * * *: *::** :*::.

H.sapiens           RKIDPVGVDYILQKLGFHHARTTIPKWLQRGVMDPLDKVLSVLIKKLGTALQDEKEKK-- 4998
D.melanogaster      KSIEPYGVDYILNKLGFSHARTTIPKWLQRGFMDPLDKVQALMMLQLLLMVRENKVERDS 5057
C.elegans           -KWTPPVLDDILKKLQIFDHRNTIPKVIQRAVLDPLDATLAASVIATLQIVDNKKTIQKF 3973
                     .  *  :* **:** : . *.**** :**..:**** . :  :      : ::*  :

H.sapiens           ---GKDKEEH------------------------------------- 5005
D.melanogaster      GASGSGKQQQ----QNHRPPTN------------------------- 5075
C.elegans           KKSRTDSMAPTPKRRDSRRSSEEVSVSIDIPDIITDISDASFRPKHN 4020
                        ...
```

B

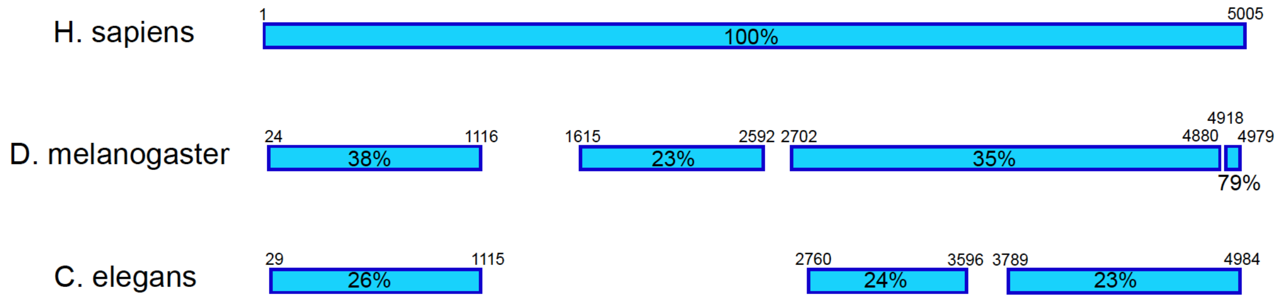
H. sapiens
1
5005
100%
D. melanogaster
24
1116
38%
1615
2592
23%
2702
4880
35%
4918
4979
79%
C. elegans
29
1115
26%
2760
3596
24%
3789
4984
23%

Figure S8: KIAA1109 protein conservation

(A) Multi-alignments of *Homo sapiens* KIAA1109, *Caenorhabditis elegans* lpd-3 and *Drosophila melanogaster* tweek proteins obtained using Clustal Omega (v1.2.3).

(B) Schematic representation of aligned segments of KIAA1109 proteins obtained using BLASTP tool on Ensembl. *Homo sapiens* protein sequence was used as query and *D. melanogaster* and *C. elegans* as subjects. Percentage of identity between the *H. sapiens* sequence and the subjects are indicated. The numbering pinpoints the coordinate of the human residues at the beginning and end of human regions with similarities. *H. sapiens* NM_015312, *D. melanogaster* NM_ 001201898 and *C. elegans* NM_001313537.

Table S1: Predicted pathogenicity and allele frequencies of the variants identified in *KIAA1109* gene

Family	Inheritance	Mutation coordinates (GRCh37/hg19)	Amino Acid change	dbSNP v147	Allele frequency (ExAC v0.3.1)	PolyPhen2 prediction (score)	PROVEAN prediction (score)	SIFT prediction (score)
LT	Compound heterozygous	Chr4:123160823; c.3986A>C	Tyr1329Cys	rs770791100	0.000041	Probably damaging (0.993)	Deleterious (-2.52)	Damaging (0.001)
		Chr4:123170727; c.5599G>A	Val1867Met	-	-	Probably damaging (0.969)	Neutral (-0.45)	Damaging (0.024)
UK	Compound heterozygous	Chr4:123164200; c.4719G>A	Met1573Ile	rs368227278	0.000008	Benign (0.000)	Neutral (-0.64)	Tolerated (0.835)
		Chr4:123171679; c.5873G>A	Arg1958Gln	-	-	Benign (0.001)	Neutral (-0.44)	Tolerated (0.594)
AL	Homozygous recessive	Chr4:123207807; c.9149C>A	Pro3050His	-	-	Probably damaging (1.000)	Deleterious (-7.36)	Damaging (0.000)
TU1	Homozygous recessive	Chr4:123230520; c.10153G>C	Gly3385Arg	-	-	Probably damaging (1.000)	Deleterious (-6.17)	Damaging (0.001)
TU2	Homozygous recessive	Chr4:123230520; c.10153G>C	Gly3385Arg	-	-	Probably damaging (1.000)	Deleterious (-6.17)	Damaging (0.001)
SA1	Homozygous recessive	Chr4:123128323; c.1557T>A	Tyr519Ter	rs730882245	-	-	-	-
SA2	Homozygous recessive	Chr4:123252480; c.11250-1G>A	His3751_Arg3822del	-	-	-	-	-
SA3	Homozygous recessive	Chr4:123258092; c.12067G>T	Glu4023Ter	-	-	-	-	-
US	Compound heterozygous	Chr4:123254885_123263438delinsG; c.11567_12352delinsG	Lys3856Argfs*44	-	-	-	-	-
		Chr4:123113479; c.997dupA	Ile333Asnfs*5	-	-	-	-	-
SG	Compound heterozygous	Chr4:123147970; c.2902C>T	Arg968Cys	-	-	Probably damaging (0.966)	Deleterious (-2.53)	Tolerated (0.058)
		Chr4:123159280; c.3611delA	Asn1204Thrfs*6	-	0.000008309	-	-	
