# Supplementary material for: KIAA1109 Variants Are Associated with a Severe Disorder of Brain Development and Arthrogryposis
Source: Am J Hum Genet. 2017 Dec 28;102(1):116–32. doi: 10.1016/j.ajhg.2017.12.002 (PMC5777449; doi:10.1016/j.ajhg.2017.12.002)
Supplement: Document S2. Article plus Supplemental Data [file mmc3.pdf]

ARTICLE

KIAA1109 Variants Are Associated with a Severe Disorder of Brain Development and Arthrogryposis

Lucie Gueneau,[1,27] Richard J. Fish,[2,27] Hanan E. Shamseldin,[3,27] Norine Voisin,[1,27] Frédéric Tran Mau-Them,[4,5,27] Egle Preiksaitiene,[6,27] Glen R. Monroe,[7,27] Angeline Lai,[8,9] Audrey Putoux,[10,11] Fabienne Allias,[12] Qamariya Ambusaidi,[13] Laima Ambrozaityte,[6] Loreta Cimbalistienė,[6] Julien Delafontaine,[14] Nicolas Guex,[14] Mais Hashem,[3] Wesam Kurdi,[13] Saumya Shekhar Jamuar,[8,15] Lim J. Ying,[8] Carine Bonnard,[16] Tommaso Pippucci,[17] Sylvain Pradervand,[1,14] Bernd Roechert,[14] Peter M. van Hasselt,[7] Michaël Wiederkehr,[1] Caroline F. Wright,[18] DDD Study, Ioannis Xenarios,[1,14] Gijs van Haaften,[7] Charles Shaw-Smith,[19] Erica M. Schindewolf,[20] Marguerite Neerman-Arbez,[2] Damien Sanlaville,[10,11] Gaëtan Lesca,[10,11] Laurent Guibaud,[11,21] Bruno Reversade,[16,22,23,24] Jamel Chelly,[4,5] Vaidutis Kučinskas,[6] Fowzan S. Alkuraya,[3,25,26,28,*] and Alexandre Reymond[1,28,*]

Whole-exome and targeted sequencing of 13 individuals from 10 unrelated families with overlapping clinical manifestations identified loss-of-function and missense variants in *KIAA1109* allowing delineation of an autosomal-recessive multi-system syndrome, which we suggest to name Alkuraya-Kučinskas syndrome (MIM 617822). Shared phenotypic features representing the cardinal characteristics of this syndrome combine brain atrophy with clubfoot and arthrogryposis. Affected individuals present with cerebral parenchymal underdevelopment, ranging from major cerebral parenchymal thinning with lissencephalic aspect to moderate parenchymal rarefaction, severe to mild ventriculomegaly, cerebellar hypoplasia with brainstem dysgenesis, and cardiac and ophthalmologic anomalies, such as microphthalmia and cataract. Severe loss-of-function cases were incompatible with life, whereas those individuals with milder missense variants presented with severe global developmental delay, syndactyly of 2nd and 3rd toes, and severe muscle hypotonia resulting in incapacity to stand without support. Consistent with a causative role for *KIAA1109* loss-of-function/hypomorphic variants in this syndrome, knockdowns of the zebrafish orthologous gene resulted in embryos with hydrocephaly and abnormally curved notochords and overall body shape, whereas published knockouts of the fruit fly and mouse orthologous genes resulted in lethality or severe neurological defects reminiscent of the probands' features.

Introduction

The advent of high-throughput sequencing led to the delineation of multiple syndromes. Neurological genetic diseases are the main class of these Mendelian disorders[1] with, for example, approximately 700 different genes confidently associated with intellectual disability (ID) and developmental delay notwithstanding that about 50% of yet unexplained ID-affected case subjects are predicted to have a genetic basis in genes remaining to be discovered.[2,3] Neurodevelopmental disorders characterized by brain malformations represent an important group among these unexplained conditions and are likely associated with mutations in genes implicated in cortical or

[1]Center for Integrative Genomics, University of Lausanne, 1015 Lausanne, Switzerland; [2]Department of Genetic Medicine and Development, University of Geneva Medical School, 1211 Geneva, Switzerland; [3]Department of Genetics, King Faisal Specialist Hospital and Research Center, Riyadh 11211, Saudi Arabia; [4]Institut de Génétique et de Biologie Moléculaire et Cellulaire (IGBMC), CNRS UMR 7104, INSERM Unité 964, 67404 Illkirch Cedex, France; [5]Laboratoire de Diagnostic Génétique, Hôpitaux Universitaires de Strasbourg, 67000 Strasbourg, France; [6]Department of Human and Medical Genetics, Faculty of Medicine, Vilnius University, 08661 Vilnius, Lithuania; [7]Department of Genetics and Center for Molecular Medicine, University Medical Center Utrecht, 3584 CX Utrecht, the Netherlands; [8]KK Women's and Children's Hospital, Singapore 229899, Singapore; [9]Lee Kong Chian School of Medicine, Nanyang Technological University-Imperial College London, Singapore 639798, Singapore; [10]Service de Génétique, Hospices Civils de Lyon, 69002 Lyon, France; [11]Centre de Recherche en Neurosciences de Lyon, INSERM U1028, UMR CNRS 5292, Université Claude Bernard Lyon 1, 69675 Bron Cedex, France; [12]Département de Pathologie, Hospices Civils de Lyon, 69002 Lyon, France; [13]Department of Obstetrics and Gynecology, King Faisal Specialist Hospital and Research Center, Riyadh 11211, Saudi Arabia; [14]Swiss Institute of Bioinformatics (SIB), 1015 Lausanne, Switzerland; [15]Duke-NUS Medical School, Singapore 169857, Singapore; [16]Institute of Medical Biology, A*STAR, Singapore 138648, Singapore; [17]Sant'Orsola-Malpighi Hospital, Medical Genetics Unit, Pavillon 11, 2nd floor, Via Massarenti 9, 40138 Bologna, Italy; [18]Deciphering Developmental Disorders (DDD) Study, Wellcome Trust Sanger Institute, Wellcome Trust Genome Campus, Hinxton, Cambridge CB10 1SA, UK; [19]Department of Clinical Genetics, Royal Devon and Exeter NHS Foundation Trust, Exeter EX1 2ED, UK; [20]Center for Fetal Diagnosis and Treatment, Children's Hospital of Philadelphia, Philadelphia, PA 19104, USA; [21]Département d'imagerie pédiatrique et fœtale, Centre Pluridisciplinaire de Diagnostic Prénatal, Hôpital Femme Mère Enfant, Université Claude Bernard Lyon 1, 69677 Bron Cedex, France; [22]Institute of Molecular and Cell Biology (IMCB), A*STAR (Agency for Science, Technology and Research), 61 Biopolis Drive, Singapore 138673, Singapore; [23]Department of Paediatrics, Yong Loo Lin School of Medicine, National University of Singapore, Singapore 119228, Singapore; [24]Amsterdam Reproduction & Development, Academic Medical Centre & VU University Medical Center, 1105 AZ Amsterdam, the Netherlands; [25]Department of Anatomy and Cell Biology, College of Medicine, Alfaisal University, Riyadh 11533, Saudi Arabia; [26]Saudi Human Genome Program, King Abdulaziz City for Science and Technology, Riyadh 12371, Saudi Arabia
[27]These authors contributed equally to this work
[28]These authors contributed equally to this work
*Correspondence: falkuraya@kfshrc.edu.sa (F.S.A.), alexandre.reymond@unil.ch (A.R.)
https://doi.org/10.1016/j.ajhg.2017.12.002.
© 2017 The Authors. This is an open access article under the CC BY license (http://creativecommons.org/licenses/by/4.0/).

cerebellar development. They can be classified into four main categories depending on the origin of the defect.[4] First are disorders due to abnormal proliferation of neuronal and glial cells including brain under-growth (microcephaly) or overgrowth (megalencephaly). Second are neuronal migration disorders that include: (1) lissencephaly, i.e., the absence or decrease of gyration responsible for a smooth brain; (2) cobblestone cortical malformations; and (3) neuronal heterotopia, i.e., the abnormal localization of a neuronal population. Third are pathologies characterized by malformations caused by postmigrational abnormal cortical organization, mainly polymicrogyria, i.e., the increase of small gyration. The last category regroups malformations of the mid-hindbrain with early anteroposterior and dorsoventral patterning defects. This phenotypic heterogeneity is paralleled by molecular heterogeneity as more than 100 genes have been implicated to date.[4–7] The causative genes can be arranged into specific biological pathways (for instance synapse structure, cellular growth regulation, apoptosis, cell-fate specification, actin cytoskeleton, and microtubule assembly) that do not necessarily correlate with the type of malformations described above, as emerging evidence suggests that brain disorders are far more heterogeneous than the classification suggests.[4,8]

We report, through description of 19 affected individuals, an autosomal-recessive brain malformation disorder with arthrogryposis caused by variants within *KIAA1109* (MIM: 611565).

Material and Methods

Enrollment

Families were recruited in Lithuania, the United Kingdom, France, Saudi Arabia, the USA, and Singapore. The institutional review boards of the Vilnius University Faculty of Medicine, NHS Foundation Trust, Hôpitaux Universitaires de Strasbourg, King Faisal Specialist Hospital and Research Center, the Children's Hospital of Philadelphia, "Hospices Civils de Lyon," and KK Women's and Children's Hospital approved this study. Participants were enrolled after written informed consent was obtained from parents or legal guardians. The clinical evaluation included medical history interviews, a physical examination, medical imaging as appropriate, and review of medical records.

Exome Sequencing and Analysis

To uncover genetic variants associated with the phenotypes of the two affected members of the Lithuanian (LT) family, we sequenced their exomes and that of their parents, as described.[9] DNA libraries were prepared from leukocytes by standard procedures. Exomes were captured and sequenced using different platforms as specified below to reach 50- to 120-fold coverage on average. Variants were filtered based on inheritance patterns including autosomal recessive, X-linked, and *de novo*/autosomal dominant. Variants with MAF $< 0.05\%$ in control cohorts (dbSNP, the 1000 Genome Project, NHLBI GO Exome Sequencing Project, the ExAC, and our in-house databases) and predicted to be deleterious by SIFT,[10] PolyPhen-2,[11] and/or UMD predictor[12] were prioritized. This exome analysis singled out compound heterozygote variants in *KIAA1109* as possibly causative in both affected siblings, prompting us to look for other individuals with overlapping phenotypes and variants in the same gene through GeneMatcher, the DDD portal, and clinical genetics meetings. These searches led to the identification of a total of 17 additional affected individuals.

In the Algerian (AL) family, exome sequencing was performed by the Centre National de Génotypage (CNG, Evry, France), Institut de Génomique, CEA. Exomes were captured with Human All Exon v5; 50 Mb (Agilent Technologies) and sequenced on a HiSeq2500 platform (Illumina) as paired-end 100 bp reads. For the Saudi Arabian (SA1–SA3) families, exome capture and sequencing was performed in conjunction with autozygome analysis as previously described.[13] For the family from Singapore (SG), exome capture, sequencing, and variant calling and analysis were performed as described.[14] For the two families from Tunisia (TU1 and TU2), exome sequencing was performed on a NextSeq500 (Illumina) after SeqCapEZ MedExome Library preparation and analyzed with BWA and GATK HaplotypeCaller. Variants with MAF $< 0.1\%$ in ExAC database and predicted to be deleterious by SIFT,[10] PolyPhen-2,[11] and Mutation Taster[15] were prioritized. The UK family's exome capture and sequencing was performed as previously described.[16] For the US family, exome capture, sequencing, and variant calling and analysis were performed as described.[16,17] The breakpoints of the paternally inherited deletion were determined by whole-genome sequencing.

Breakpoint Mapping by Whole-Genome Sequencing

100 ng of genomic DNA were sheared using Covaris with a target fragment size of 500 bp. The sequencing library was prepared using Tru-Seq DNA PCR-free Sample Prep Kit (Illumina) and 100-bp paired-end reads sequenced on a HiSeq 2500 platform (Illumina). The PCR-free kit was used to prepare the library in order to avoid PCR duplicates. Sequence-control, software real-time analysis, and bcl2fastq conversion software v.1.8.4 (Illumina) were used for image analysis, base calling, and demultiplexing. Purity-filtered reads were adapters- and quality-trimmed with FastqM*cf*. v.1.1.2 and aligned to the human_g1k_v37_decoy genome using BWA-MEM (v.0.7.10[18]). PCR duplicates were marked using Picard tools (v.2.2.1). We obtained a sequence yield of 11.4 Gb of aligned bases with a 3.6× mean coverage. Aligned reads within the *KIAA1109* locus were visualized and evaluated using Integrative Genomics Viewer (IGV) in search of chimeric inserts. We identified a single pair of paired-ends reads mapping unequivocally 8,971 bp apart within *KIAA1109* allowing us to map the paternally inherited deletion of the US proband breakpoints within exon 68 and intron 72. The breakpoints were then finely mapped with Sanger sequencing to coordinates chr4:123254885 and chr4:123263438 (hg19) (Figure S1).

Zebrafish Manipulations, CRISPR/Cas9 Editing, and Design of Morpholinos

Zebrafish animal experimentation was approved by the Ethical Committee for Animal Experimentation of the Geneva University Medical School and the Canton of Geneva Animal Experimentation Veterinary authority. Wild-type TU (Tübingen) zebrafish were maintained in standard conditions (26°C–28°C, water conductivity at 500 μS [pH 7.5]). Embryos obtained by natural matings were staged according to morphology/age.

Zebrafish *kiaa1109* mutant lines were developed using CRISPR-Cas9-mediated genome editing. Using the ZiFiT online tool,[19] we

identified three suitable 20-nucleotide sites upstream of protospacer adjacent motifs (PAM) for *S. pyogenes* Cas9 and targeting *kiaa1109* exons 1, 4, and 7 (numbering according to GenBank: NM_001145584.1). Annealed oligonucleotides carrying the 20-nucleotide target sequence were ligated into pDR274 (Addgene plasmid # 42250), and clones verified by Sanger sequencing, linearized, and used for *in vitro* transcription of single-guide RNAs (sgRNAs) using the MEGAshortscript T7 Transcription Kit (ThermoFisher). sgRNAs were mixed with recombinant Cas9 nuclease (PNA Bio), Danieau buffer, and phenol red as a tracer, and approximately 1 nL injected into early zebrafish embryos. Each injection contained 0.25 ng of sgRNA and 0.5 ng of Cas9 per nL. Evidence for genome editing was assessed qualitatively by PCR amplification around the target sites in each exon in injected embryo lysates. Heterogeneous PCR products, consistent with mosaic editing, was seen as smeared bands by gel electrophoresis, compared to uninjected embryos (not shown). Injected fish embryos were raised to adulthood and screened for their ability to transmit mutant *kiaa1109* alleles by out-crossing and PCR genotyping. PCR products were cloned with pCRII TOPO (ThermoFisher) to separate alleles, and colony PCRs were sequenced to detect germline transmission of potential *kiaa1109* frameshift alleles. Out-crossed F1 embryos were raised to adulthood for mutations detected in exon 1, 4, and 7, as separate lines. F1 adult fish were tail-clipped, targeted exons were amplified by PCR, and PCR products were cloned to pCRII TOPO (ThermoFisher) to identify specific *kiaa1109* mutant alleles in heterozygosity by colony PCR and DNA sequencing. Heterozygous F1 fishes carrying the same *kiaa1109* mutation were then in-crossed to assess embryonic survival and phenotype in homozygosity. *kiaa1109* genotyping for embryos from these crosses was made using PCR, amplifying the target exon regions. Products from wild-type, heterozygous, or homozygous mutant amplicons were distinguished by gel electrophoresis. Details of the sgRNA target sites, representative mutant allele sequencing chromatograms, and predicted frameshifts for the three *kiaa1109* mutant lines described are given in Figures S2 and S3.

To knock down *kiaa1109* (GenBank: NM_001145584.1) in zebrafish, we designed two non-overlapping splice-blocking MOs (morpholinos) targeting pre-mRNA: (1) sbE4MO- 5′-TGTTCTGT TTTTGCACTGACCATGT-3′ and (2) sbE2MO- 5′-CAACATTGAGA CAGACTCACCGATG-3′ (Gene Tools) that target the exon 4/intron 4 and exon 2/intron 2 boundaries, respectively. The standard Ctrl-MO (5′-CCTCTTACCTCAGTTACAATTTATA-3′) (Gene Tools) without any targets in the zebrafish genome was used for mock injections. MOs were dissolved in nuclease-free water and their concentrations determined by NanoDrop. The fish were injected at 1- to 2-cell stages (1–2 nL) using phenol red as a tracer in Danieau buffer. The following amounts of MO: 3.35 and 6.7 ng of sbE4MO; 5.6, 11.3, 16.9, and 22.3 ng of sbE2MO and the equivalent of Ctrl-MO for the higher doses were injected into wild-type zebrafish embryos, respectively. Uninjected, standard control MO, and *kiaa1109* MO-injected embryos were collected at 2 dpf and total RNAs were isolated using standard Trizol protocol (Invitrogen). 1 μg of total RNA from each sample was used to synthesize cDNA with the Superscript III kit with Oligo d(T) primers (Invitrogen). Dilutions 1/20 of cDNA were used for standard PCR reactions (JumpStart RED Taq ReadyMix, Sigma-Aldrich). Basic quantifications of agarose gels were performed with ImageQuant TL software (GE Healthcare). We assessed embryos for morphological changes at 2 days post-fertilization. We grouped the embryos into four classes by morphology: normal embryos, embryos with clear midbrain and/or hindbrain ventricle swelling, curved embryos, and embryos with both phenotypes. The degree of hydrocephaly was not measured; hydrocephaly was assessed by clear deviation from the normal embryo morphology (see Results). Curved embryos showed caudal axis curvature. They were clearly distinguishable from the straight anterior-posterior axis of normal 2-day-old embryos (see Results). The most severely affected embryos had a combination of hydrocephaly and caudal axis curvature (see Results).

Results

We first identified compound heterozygous missense variants in *KIAA1109* in a Lithuanian family with two affected siblings (LT.II.1 and LT.II.2) presenting with a constellation of severe global developmental delay, cerebral parenchymal rarefaction and ventriculomegaly (observed at 20 months of age), plagiocephaly, paretic position of hands and feet at birth, early-onset epilepsy, muscle hypotonia, stereotypical movements, hypermetropia, and lack of walking function (Table 1, Figure 1). As a homozygous stop-gain allele in this gene was suspected to cause a syndromic neurological disorder in a fetus (described in more details in this manuscript as fetus SA1.II.1) with cerebellar malformations, hydrocephalus, micrognathia, club feet, arthrogryposis with flexed deformity, pleural effusion, and death 1 hr after birth,[13] we hypothesized that *KIAA1109* variants cause an autosomal-recessive (AR) brain development disorder with arthrogryposis.

Our searches for more case subjects led to the identification of a total of 19 affected individuals from 10 families (including 6 undiagnosed miscarriages) recruited in Algeria (AL), Lithuania (LT), Saudi Arabia (SA1–SA3), Singapore (SG), Tunisia (TU1, TU2), the United Kingdom (UK), and the United States of America (US) (Figure 2A). Genetic variants associated with the complex phenotype of interest were uncovered through exome sequencing of the affected individuals and their healthy parents with the exception of SG.II.4. We found only one gene, *KIAA1109*, compliant with AR Mendelian expectations and bearing two putatively deleterious variants in all affected individuals. GENCODE[20] catalogs in Ensembl 16 isoforms of *KIAA1109*; two encode the full-length 5,005-amino acids protein, six have no coding potentials, and the remaining eight isoforms encode protein of lengths varying from 164 to 1,674 amino acids. All the mutations reported in this manuscript affect the full-length GenBank: NP_056127 protein. Consistent with consanguineous unions, the affected members of families AL, TU1/TU2, SA1, SA2, and SA3 were homozygous for variants c.9149C>A (p.Pro3050His), for c.10153G>C (p.Gly3385Arg), for c.1557T>A (p.Tyr519Ter), for c.11250−1G>A (r.11250_11465del, p.His3751_Arg3822del), and for c.12067G>T (p.Glu4023Ter), respectively, whereas the affected individuals from LT, SG, UK, and US families were heterozygote for c.3986A>G (p.Tyr1329Cys) and c.5599G>A (p.Val1867Met), for c.2902C>T (p.Arg968Cys) and

c.3611delA (p.Asn1204Thrfs*6), for c.4719G>A (p.Met1573Ile) and the *de novo* c.5873G>A (p.Arg1958Gln), and for c.997dupA (p.Ile333Asnfs*5) and the deletion g.123254885_123263438delinsG (c.11567_12352delinsG, p.Lys3856Argfs*44), respectively (nomenclature according to GenBank: NM_015312.3, NP_056127.2; Figures 2A and 2B, Table S1). The fact that families TU1 and TU2 are not known to be related suggests a Tunisian founder effect of variant c.10153G>C (p.Gly3385Arg). Sanger sequencing in each family confirmed the anticipated segregation of the *KIAA1109* variants, with the exception of family TU1 whose parents declined to be assessed. It also confirmed the genetic status of the SG.II.4 affected sibling (Figure 2A). All variants are either absent or encountered (as heterozygous variants) with a frequency lower than 1/10,000 in ExAC (v.0.3.1)[21] (Table S1). The missense variants are predicted to be functionally damaging at least by two of the three PolyPhen-2,[11] Provean,[22] and SIFT[10] predictors with the exception of the UK.II.1 variants predicted to be benign, neutral, and tolerated, respectively (Table S1). They might be under "compensated pathogenic deviation in human, a phenomenon that contributes to an unknown, but potentially large, number of false negatives to the evaluation of functional sites" as demonstrated in Jordan et al.[23] Missense variants and CNVs are underrepresented compared to expectation in ExAC (missense Z score = 4.97; CNV Z score = 0.77) indicating that *KIAA1109* is under constraint. The identification of 50 LoF variants compared to the 176.1 expected, while not significant with a pLI = 0.0, does not contradict this hypothesis. In agreement with a possible contributing role of bi-allelic *KIAA1109* LoF variants to the phenotype of affected individuals SA1.II.1, SA2.II.1, SA3.II.1, and US.II.3, ExAC does not report homozygous LoF variants in *KIAA1109*. The splice variant c.11250−1G>A identified in fetus SA2.II.1 is predicted to abolish the consensus acceptor site of intron 66.[24] A prediction validated by our RT-PCR experiments that showed a partial skipping of 216-nucleotides-long exon 67 in lymphoblastoid cell line of the affected SA2.II.1 fetus (Figure S4). The corresponding transcript would encode a protein lacking 72 amino acids. All missense variants identified in the AL, LT, SG, TU, and UK families affect highly conserved residues within evolutionary conserved region of the encoded protein (Figure S5).

As exemplified by the LT.II.1 and LT.II.2 siblings and the SA1.II.1 proband, the phenotype of the 19 affected individuals ranges from global developmental delay with/without inability to stand to stillbirth. Many of the more severely affected case subjects harbor homozygous or compound heterozygote truncating alleles (families SA1–SA3 and US) (Table 1, Supplemental Note). While the phenotype of proband SA1.II.1 is summarized above, SA2.II.1 and SA3.II.1 stillborn fetuses shared hydrocephalus, cerebellar hypoplasia, arthrogryposis, and skeletal anomalies (Figures 3 and S6). Proband SA2.II.1 had bilateral overlapping fingers, apparent contractures of the hands and feet, and bilateral sandal gaps, along with shortened long bones and nuchal thickening. He also presented with absence of corpus callosum and abnormal kidneys. Proband SA3.II.1 showed skin edema, bilateral talipes, and arthrogryposis (Figure 3; Table 1; Supplemental Note). The US.II.3 stillborn fetus resulted from a 3rd pregnancy attempt of the couple (Figure 2A). The fetus demonstrated major central nervous anomalies including thin cerebral parenchyma with lissencephalic pattern, prominent germinal matrix, ventriculomegaly, brain stem vermian dysgenesis (kinked brain stem and elongated pons), and absence of corpus callosum, as well as closed spinal defect at L4-L5, associated with extra-central nervous anomalies including coarctation of the aorta, small omphalocele, echogenic bowel, hydrops, cystic hygroma, pleural effusion, possible anal atresia, low-set ears, short penis, clinodactyly, talipes, and abnormal posturing of the limbs (Figure 3). The SG family had four pregnancy attempts; two resulted in miscarriages (SG.II.2 and SG.II.3) and two in fetuses who did not pass the first semester (Figures 2A and 4). The SG.II.1 elder brother had minimal respiratory effort at birth and required immediate intubation and mechanical ventilation. He presented with macrocephaly, hypertelorism, posteriorly rotated ears, flattened nasal bridge, congenital cataract, and microphthalmia. He had generalized arthrogryposis and bilateral congenital talipes equinovarus. He also had hypotonia and an ano-rectal malformation with recto-perianal fistula (Figure 4). Brain MRI showed major cerebral parenchymal thinning with lissencephalic aspect, severe ventriculomegaly, absence of corpus callosum, and severe cerebellar and pontine hypoplasia (Figure 4). He passed away at 3 months of age from pneumonia and septic shock. The SG.II.4 younger sibling was remarkably similar to his elder with hypertelorism, bilateral low-set ears, short nose, anteverted nares, bilateral congenital cataract, microphthalmia, webbed neck, bilateral structural congenital talipes equinovarus, generalized arthrogryposis, and hypotonia. Brain MRI showed severe hydrocephalus with marked thinning of the cerebral parenchyma. The corpus callosum was absent, the cerebellum and brainstem were hypoplastic, and there was a pontomesencephalic kink. He remained ventilator dependent from birth and passed away at 1 month of age (Figure 4). The AL.II.1 fetus presented with an equally severe phenotype so the parents elected to terminate the pregnancy. He showed multiple brain malformations including hydrocephalus, vermis fusion, lamination defect of cerebellar cortex, and absence of the corpus callosum, combined with arthrogryposis with flexed deformity and bilateral adductus thumbs, diffuse effusion, and other clinical features (Figures 3 and S6; Table 1; Supplemental Note). Affected individuals and fetuses TU1.II.1, TU1.II.4, and TU2.II.2 had arthrogryposis and the same cerebral malformative pattern, associating cerebellar and brainstem dysgenesis, parenchymal thinning with major lack of gyration, corpus callosum agenesis, and hyperplastic germinal matrix protruding within ventriculomegaly. The severity of features of the AL.II.1

Table 1. Overlapping Clinical Features of Individuals with KIAA1109 Variants

Family #	Individual #	Gender, Age	Ethnicity	Gene Mutations	ID	Mutation Coordinates (GRCh37/hg19)	Cerebral Anomalies (Pre/Post-natal Images) and Pathological Findings	Head and Face
LT	LT.II.1 (brother)	male, 13 yo	Lithuanian	compound heterozygote	severe, global developmental delay, no language, cannot stand or walk without support	Chr4:123160823; c.3986A>C, Chr4:123170727; c.5599G>A	post-natal brain MRI: small posterior fossa arachnoid cyst, discrete vermian atrophy, slight increase of the fluid-filled retro and infra-cerebellar space and mild enlargement of subarachnoid spaces of frontal regions.	plagiocephaly
LT	LT.II.2 (sister)	female, 7 yo	Lithuanian	compound heterozygote	severe, global developmental delay, no language, cannot sit or stand without support	Chr4:123160823; c.3986A>C, Chr4:123170727; c.5599G>A	post-natal brain MRI: discrete parenchymal rarefaction involving the frontal lobes	plagiocephaly
UK	UK.II.1, DDD# 263241	female, 11 yo	British	compound heterozygote with one *de novo* missense mutation	global developmental delay, mild to moderate learning disability	Chr4:123164200; c.4719G>A and Chr4:123171679; c.5873G>A	prenatal imaging (US and MRI): major microcephaly (HC -5 SD) with reduced white matter volume and mild ventriculomegaly	hypertelorism, slightly upslanting palpebral fissures
AL	AL.II.1	male, termination of pregnancy at 21 weeks of amenorrhea	Algerian	homozygous missense mutation	not applicable	Chr4:123207807; c.9149C>A	prenatal US findings: triventricular ventriculomegaly and corpus callosum agenesis; neuropathological findings: absence of cortical lamination and diffuse migration anomalies within a thin parenchymal mantle, ventriculomegaly, and voluminous germinal matrix. Corpus callosum was not identified. Infra-tentorial space: hypoplasia of the pons with absence of the longitudinal and transversal fibers and dysplasia of the cerebellum characterized by lack of foliation and poorly identified vermis; narrowing of the aqueduct	hypertelorism, posteriorly rotated ears

Eyes	Mouth	Joints	Limbs	Gastro-intestinal	Urogenital	Heart	Muscles	Behavior	Other Symptoms
hypermetropia, strabismus, astigmatism	delayed eruption of permanent teeth	mild contractures of large joints	syndactyly of 2nd and 3rd toes, hands and feet paresis at birth, talipes valgus	normal	scrotum hypoplasia	none	muscle hypotonia, atrophy	stereotypic movements, spontaneous paroxysms of laughter	early-onset epilepsy
hypermetropia, strabismus, astigmatism	normal	mild contractures of large joints	paretic position of hands and feet in infancy, talipes valgus	chronic constipation	none	none	muscle hypotonia, atrophy	stereotypic movements	early onset epilepsy, dermatitis, psoriasis
ocular motor apraxia, hypermetropia, strabismus	dental crowding, high palate	mild bilateral talipes managed by physiotherapy only; asymmetry of the thorax	syndactyly of 2nd and 3rd toes, 5th toe clinodacytly, hallux valgus	gastro-esophageal reflux	none	complex congenital heart disease (tetralogy of Fallot with pulmonary atresia)	none	poor concentration, immature behavior with minor self-harm (head-banging) when angry/frustrated	none
bilateral cataract with crystalline fibers of variable size and orientation	retrognathism, big horizontalized mouth	arthrogryposis (flexed deformity of shoulders, elbow and hips, and bilateral adductus thumbs)	bilateral equinovarus foot	choanal atresia	scrotum hypoplasia	pericardial effusion	not available	not applicable	slight pleural effusion, peritoneal effusion, dilatation of lymph vessels in lung with lympho-hematopoietic elements

(*Continued on next page*)

Table 1. *Continued*

Family #	Individual #	Gender, Age	Ethnicity	Gene Mutations	ID	Mutation Coordinates (GRCh37/hg19)	Cerebral Anomalies (Pre/Post-natal Images) and Pathological Findings	Head and Face
TU1	TU1.II.1	female, died at 3 days of age	Tunisian	homozygous missense mutation	not applicable	Chr4:123230520; c.10153G>C	prenatal imaging (US and MRI): cerebellar hypoplasia and brainstem dysgenesis (flat and elongated pons and slightly kinked brainstem with increased fluid filled retro-cerebellar spaces); severe parenchymal thinning with major lack of gyration (lissencephalic aspect) associated with voluminous germinal matrix protruding within moderate ventriculomegaly and absence of corpus callosum. Cephalic biometry was normal.	hypotelorism
TU1	TU1.II.4	male, termination of pregnancy at 23 weeks	Tunisian	homozygous missense mutation	not applicable	Chr4:123230520; c.10153G>C	prenatal US findings: severe parenchymal thinning with lack of gyration associated with ventriculomegaly and corpus callosum agenesis. Neuropathological findings: complete corpus callosum agenesis, ventricular dilatation, severe cortical malformations with a reduced cortical plate, neuronal depletion, heterotopia within white matter, dysplasia of brainstem and cerebellum.	none
TU2	TU2.II.2	female, died at 12 days of age	Tunisian	homozygous missense mutation	not applicable	Chr4:123230520; c.10153G>C	prenatal imaging (US and MRI): cerebellar hypoplasia and dysgenesis associated to severe brainstem dysgenesis characterized by flat and elongated pons and slightly kinked brainstem with increased fluid filled retro-cerebellar spaces. Corpus callosum was not identified. Supratentorial anomalies include severe parenchymal thinning associated with lissencephalic aspect as well as voluminous germinal matrix protruding within severe ventriculomegaly.	none
SA1	SA1.II.1, 13DG1900	female, death at 1 hr after delivery	Saudi	homozygous nonsense mutation	not applicable	Chr4: 123128323; c.1557T>A	prenatal US findings: severe ventriculomegaly with supratentorial cerebral mantle thinning associated with cerebellar hypoplasia	small eyes, low-set ears

Eyes	Mouth	Joints	Limbs	Gastro-intestinal	Urogenital	Heart	Muscles	Behavior	Other Symptoms
none	deep palate	left club foot	long fingers	none	none	left heart hypoplasia	not available	not applicable	none
none	none	arthrogryposis (hip and shoulder contractures)	clenched hands, camptodactyly, club feet	none	none	none	not applicable	not applicable	none
microphthalmia, blepharophimosis	none	club feet	club feet and hands	none	none	none	hypotonia	not applicable	narrow chest
small eyes	micrognathia	severe arthrogryposis (fixed elbows, fixed bilateral talipes, bilateral overlapping fingers, bilateral clinodactyly)	bilateral club foot	not available	not available	not available	not available	not applicable	pleural effusion

(*Continued on next page*)

Table 1. *Continued*

Family #	Individual #	Gender, Age	Ethnicity	Gene Mutations	ID	Mutation Coordinates (GRCh37/hg19)	Cerebral Anomalies (Pre/Post-natal Images) and Pathological Findings	Head and Face
SA2	SA2.II.1, 15DG0595	female, stillborn	Saudi	homozygous splice mutation	not applicable	Chr4:123252480; c.11250−1G>A	prenatal US findings: hydrocephalus, absent corpus callosum, hypoplastic cerebellum	not available
SA3	SA3.II.1, 15DG1933	female, stillborn	Saudi	homozygous nonsense mutation	not applicable	Chr4:123258092; c.12067G>T	prenatal US findings: hydrocephalus, hypoplastic cerebellum	not available
US	US.II.3	male, termination of pregnancy at 19 weeks	Caucasian	compound heterozygote	not applicable	Chr4:123113479; c.997dupA and Chr4: 123254885_ 123263438del; c.11567_ 12352delinsG	prenatal imaging (US and MRI): severe ventriculomegaly, thin cerebral parenchyma and cortical mantle associated with lissencephalic pattern, prominent germinal matrix, brain stem and vermian dysgenesis (kinked brain stem) and elongated pons; corpus callosum agenesis	low-set ears, webbed neck
SG	SG.II.1	male, died at 3 months of age	Chinese	compound heterozygote	not applicable	Chr4:123147970; c.2902C>T and Chr4:123159280; c.3611delA	post-natal MRI: supratentorial findings include both severe parenchymal (or cerebral mantle) thinning and smooth cortical surface, germinolytic cysts involving voluminous germinal matrix protruding within severe ventriculomegaly without any identification of corpus callosum. Infratentorial findings include severe cerebellar hypoplasia with severe brain-stem dysgenesis characterized by a kinking aspect.	macrocephaly; hypertelorism; posteriorly rotated ears; flattened nasal bridge
SG	SG.II.4	male, died at 1 month of age	Chinese	compound heterozygote	not applicable	Chr4:123147970; c.2902C>T and Chr4:123159280; c.3611delA	post-natal MRI: supratentorial findings include both severe parenchymal (or cerebral mantle) thinning and smooth cortical surface, germinolytic cysts involving voluminous germinal matrix protruding within severe ventriculomegaly without any identification of corpus callosum. Infratentorial findings include severe cerebellar hypoplasia with severe brain-stem dysgenesis characterized by a kinking aspect	macrocephaly; hypertelorism; bilateral low-set ears, short nose; anteverted nares

Eyes	Mouth	Joints	Limbs	Gastro-intestinal	Urogenital	Heart	Muscles	Behavior	Other Symptoms
not available	not available	arthrogryposis multiplex	bilateral overlapping fingers, bilateral cleft feet, bilateral cleft toes, and bilateral sandal gaps	normal	bilaterally abnormal kidneys	not available	not available	not applicable	skeletal shortening, nuchal thickening
not available	not available	arthrogryposis	bilateral talipes	normal	normal	absent fetal heart	not available	not applicable	skin edema
normal	unremarkable	severe arthrogryposis with flexion contractures and pterygia, hyperflexed wrists, bilateral clinodactyly	bilateral talipes	normal	echogenic malrotated bowel without ascites, short penis with bulbous shaft	coarctation of the aorta	muscle atrophy	not applicable	low conus non-immune hydrops with scalp edema, cystic hygroma, anal atresia, bilateral pleural effusion
congenital cataract; microphthalmia	no structural anomalies	arthrogryposis (involving bilateral shoulders, elbows, wrists, hands, knees)	bilateral structural congenital talipes equinovarus (CTEV)	ano-rectal malformation with recto-perianal fistula	no structural anomalies	small atrial septal defect/ patent foramen ovale	hypotonia	not applicable	excess skin folds of neck
congenital cataracts; microphthalmia	no structural anomalies	arthrogryposis (involving bilateral elbows, wrists, hands, knees, hips)	bilateral structural congenital talipes equinovarus (CTEV)	normal	no structural anomalies	small to moderate fenestrated atrial septal defect	hypotonia	not applicable	webbed neck; inverted nipples

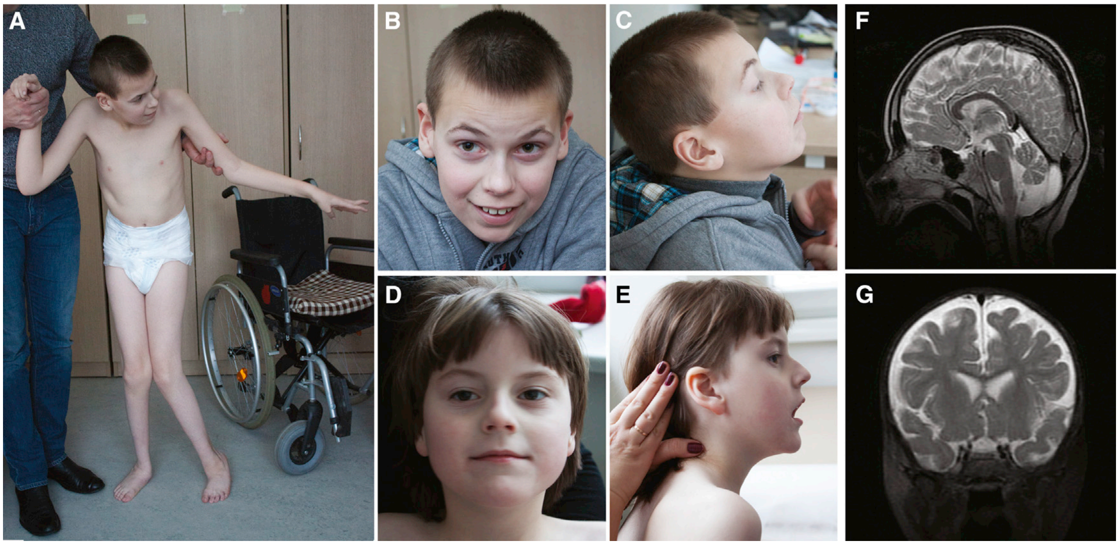

Figure 1. Pictures and Brain MRI from Surviving Individuals
Front and side views of the LT affected brother LT.II.1 (A–C) and sister LT.II.2 (D, E) at the ages of 13 years and 7 years, respectively. Brain MRI images of affected individual LT.II.1 at age of 8 years showed small posterior fossa arachnoid cyst, discrete vermian atrophy, and slight increase of the fluid-filled retro and infra-cerebellar space (F). Brain MRI images of affected individual LT.II.2 at age of 1 year showed discrete parenchymal rarefaction involving mainly the frontal lobes (G).

fetus, the SG.II.1 and SG.II.4 siblings, and the TU1.II.1, TU1.II.4, and TU2.II.2 Tunisian affected individuals and their resemblance with those observed in carriers of truncating variants suggest that the missense variants p.Pro3050His, p.Gly3385Arg, and p.Arg968Cys act as LoF or strong hypomorphs. Consistent with this hypothesis, the C>T transition in exon 24 of the latter variant is predicted to alter an exonic splicing enhancer site and thus proper splicing. More experiments are warranted to further demonstrate these assumptions.

All case subjects compatible with life carry missense variants (Figure 2; Table S1; Supplemental Note). Whereas the two LT.II.1 and LT.II.2 Lithuanian siblings are briefly described above, the UK.II.1 British proband showed global developmental delay, microcephaly, absence of the pulmonary valve, tetralogy of Fallot and ventricular septal defect, ocular motor apraxia, hypermetropia, dental crowding, 5th toe clinodactyly, syndactyly of the 2nd and 3rd toe like the LT.II.1 elder sibling, hallux valgus, and pes planus (Supplemental Note).

In line with the clinical presentation of LoF affected individuals, ablation in fruit flies and mice of the *KIAA1109* orthologs, *tweek* and *Kiaa1109*, respectively, resulted in lethality. Whereas *Kiaa1109*$^{-/-}$ mice engineered and phenotyped by the International Mouse Phenotyping Consortium[25,26] exhibited complete penetrance of preweaning lethality, some rare homozygous *tweek* mutants survive to adulthood.[27] These survivors presented with severe neurological defects such as seizures, inability to stand upright for long periods or walk, suggesting that *tweek* was involved in synaptic function.[27] These results further support a causative role of LoF of *KIAA1109* in the phenotypes observed in families AL, SA1–SA3, and US. Consistent with this hypothesis, *KIAA1109* has higher expression in the pituitary, the cerebellum, and the cerebellar hemispheres according to GTex.[28]

To further assess the consequences of decreased *KIAA1109* activity, we used both CRISPR/Cas9 genome editing and morpholinos (MO) technology in zebrafish. We generated three different stable lines with frameshift variants in exons 1, 4, and 7 of *kiaa1109*. Crosses of each heterozygote line with themselves suggest that these mutations are not lethal. To explain the discrepancy between these results and what was observed in mice and fruit flies, we profiled the transcriptome of homozygotes larvae. While we observed subtle differences between homozygous fish and their wild-type clutchmates, by and large we see no changes in expression of the different *kiaa1109* exons (Table S2). Our results suggest that the expression of *kiaa1109* isoforms containing only downstream exons encode proteins providing all the non-redundant functions of kiaa1109. More work is warranted to assess whether the engineered variants are inducing nonsense-mediated decay and whether there is any maternal contribution. In parallel, we knocked down *kiaa1109* using two different non-overlapping morpholinos (MOs). While we are aware that unspecific effects have been reported when using MOs,[29] we still favored this approach to mimic to a certain degree the situation observed in the LT.II.1 and LT.II.2 siblings and the UK.II.1 affected individual. Injection of early zebrafish embryos with 6.7 ng of sbE4MO resulted in a 50% reduction of the *kiaa1109* transcripts through skipping of 65 nucleotides long exon 4 (Figure S7). 49% of morphants were hydrocephalic or presented with other head defects, whereas only 3% of the mock-injected fish showed such phenotypes (Figures 5A

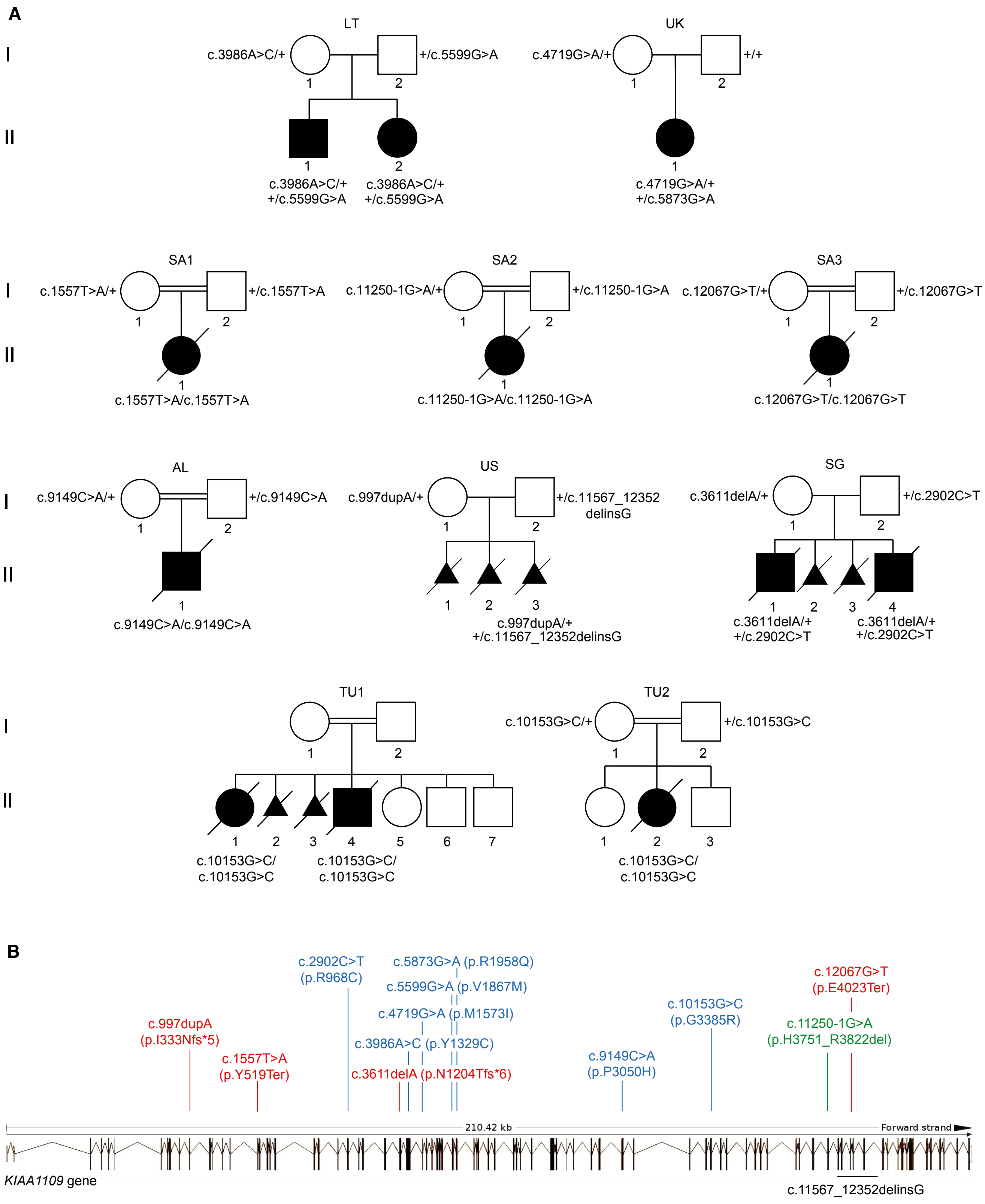

Figure 2. ***KIAA1109* Pedigrees and Variants**

(A) Pedigrees of the ten families carrying *KIAA1109* variants. The affected individuals of the Lithuanian (LT), Singaporean (SG), British (UK), and American (US) families are compound heterozygotes for rare variants, whereas the probands of the Algerian (AL), Saudi Arabian (SA1–SA3), and Tunisian (TU1, TU2) consanguineous families are homozygous for *KIAA1109* variants.

(B) Distribution of variants along the schematically represented 86 exons of *KIAA1109*. Missense variants are depicted in blue, nonsense in red, and the splice site variant in green. The extent of the deletion identified in the proband of the US family is indicated in black below.

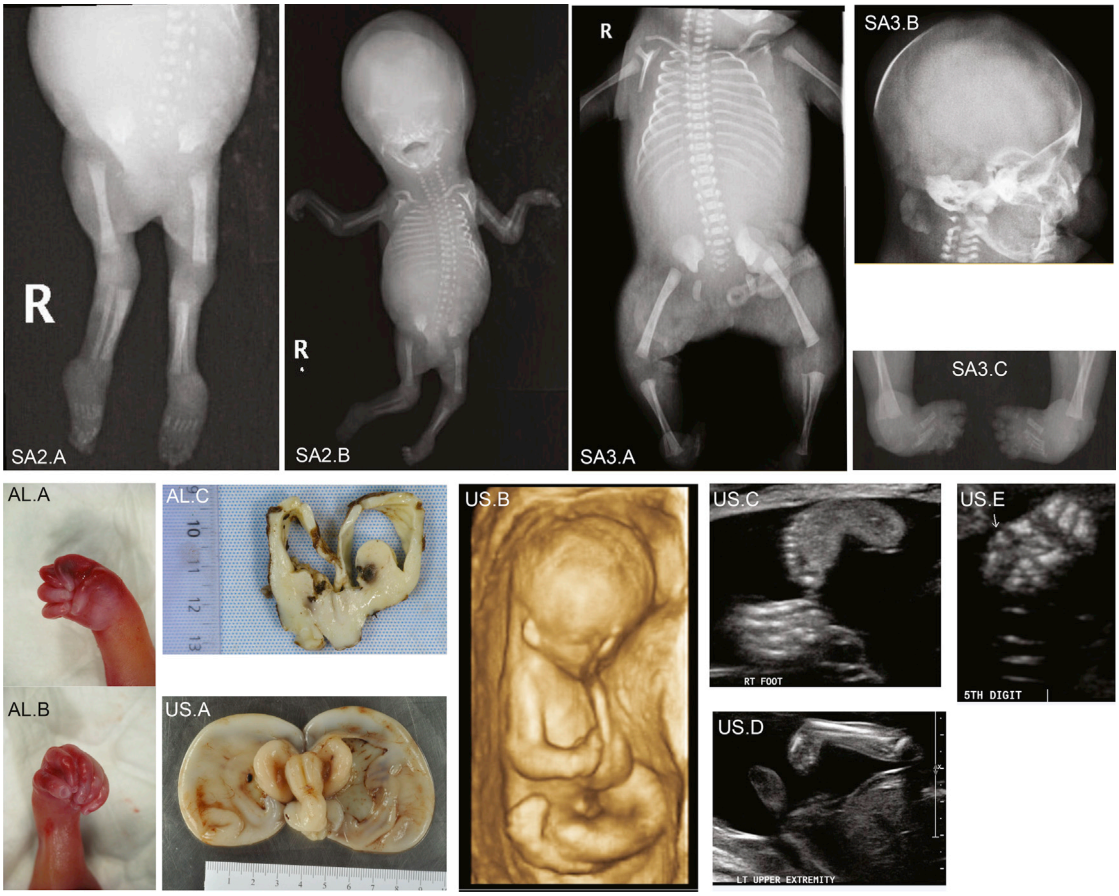

Figure 3. Ultrasound, X-Rays, and Autopsy Images of the SA2.II.1, SA3.II.1, AL.II.1, and US.II.3 Fetuses
X-ray images showing arthrogryposis of SA2.II.1 fetus (SA2.A and SA2.B).
X-ray images showing SA3.II.1 skeleton (SA3.A), head (SA3.B), and club feet (SA3.C).
Autopsy pictures from the AL.II.1 fetus showing right (AL.A) and left (AL.B) adductus thumbs of the fetus, and dilatation of cerebral ventricles with agenesis of corpus callosum (AL.C).
Autopsy image of the brain from US.II.3 fetus showing hydrocephalic brain with diaphanous pallium (US.A). The colliculi appear as single elongated ridges separated by a midline futter and the midline appears angulated on the brainstem, which is small as is the cerebellum. Antenatal ultrasound scan showed general arthrogryposis (US.B), one hyperflexed wrist (US.C), club feet (US.D), and bilateral clinodactyly of one hand (US.E).

and 5B). The second MO, sbE2MO, acts through retention of 88-nucleotide-long intron 2 and decreases *kiaa1109* levels by about 50% after injection of a large dose of 16.9 ng (Figure S7). Under such condition, about two-thirds (66%) of MO-sbE2 morphants were hydrocephalic or presented with other head defects compared to 8% of mock-injected fish affected at such dose (Figures 5A and 5C). Of note, rescue experiments of MO-injected zebrafish could not be performed due to the large size of the *kiaa1109* transcript. In summary, knockdowns of the zebrafish *KIAA1109* ortholog using two different MOs resulted in hydrocephalic animals reminiscent of probands' features.

Discussion

Data aggregation of exome sequencing from multiple laboratories allowed associating homozygous and compound heterozygote variants in *KIAA1109* with a syndrome that we suggest naming Alkuraya-Kučinskas syndrome (AKS), as these clinicians first described affected individuals at the severe and mild ends of the phenotype, respectively. AKS combines severe brain malformations (13 affected individuals out of 13), in particular hydrocephaly/ventriculomegaly (11/13) and corpus callosum agenesis (8/13) with arthrogryposis/contractures (10/13) and/or talipes valgus/talipes equinovarus/club foot (12/13) and heart defects (6/13).

AKS presents multiple overlaps with Aase-Smith syndrome 1 (ASS1 [MIM: 147800]) characterized by arthrogryposis, hydrocephalus, Dandy-Walker malformation, talipes equinovarus, cardiac defects, and risks of stillbirth or premature death. However, the two described families with one father and two children affected[30] and one mother and her affected daughter[31] are suggestive of a dominant rather than a recessive mode of transmission. Consistent with the view that AKS and ASS1 have different

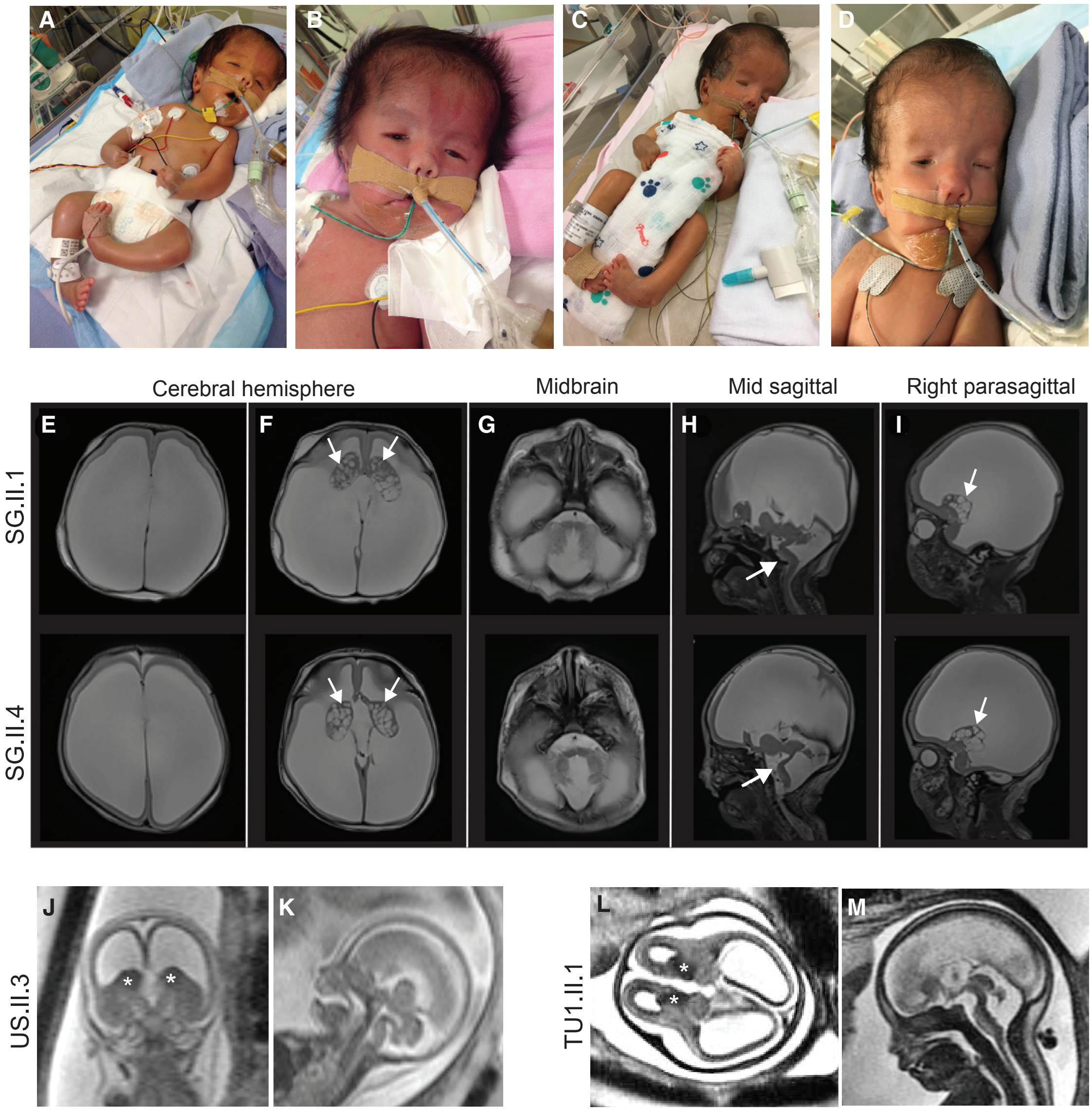

Figure 4. Pictures and Brain MRI Images of the SG.II.1 and SG.II.4 Babies and US.II.3 and TU1.II.1 Fetuses
(A–D) Photographs of SG.II.1 (A and B) and SG.II.4 (C and D) babies showing their whole bodies (A and C) and a close up of their faces (B and D).
(E–I) Brain MRI images of the elder brother SG.II.1 (top) and the younger brother SG.II.4 (bottom). Axial T2 weighted images showed severe ventriculomegaly, associated with severe thinning of the brain parenchyma (E, F). The brain parenchyma showed absence of normal gyral/sulcal pattern with smooth appearance in keeping with lissencephaly (E, F). Corpus callosum appeared to be absent (E, H). Note the prominent germinal matrix with germinolysis cysts (solid arrows) (F, I). The pons and cerebellum appeared hypoplastic with dilatation of the 4th ventricle (G, H) and Z shaped appearance of the brainstem (solid arrows) (H).
(J–M) Coronal (J), axial (L), and midsagittal (K, M) T2-weighed fetal prenatal MRI images of US.II.3 at 18.5 weeks of pregnancy (J and K) and TU1.II.1 at 28 weeks of pregnancy (L and M) demonstrating a similar imaging pattern including thin parenchyma (lissencephalic aspect), prominent germinal matrix marked by an asterisk, ventriculomegaly, and brain stem and vermian dysgenesis (kinked brain stem and elongated pons).
In summary, we observe a similar brain malformation pattern both prenatally—US.II.3 in (J) and (K), TU1.II.1 in (L) and (M), AL.II.1 (see text), TU1.II.4 (see text), and TU2.II.2 (see text)—and postnatally (SG.II.1 [E–I top] and SG.II.4 [E–I bottom]).

etiologies, both ASS1-affected families presented individuals affected with cleft palate, a birth defect not present in the 13 *KIAA1109* individuals described here, including those at the severe end of the phenotypic spectrum. Interestingly, we have identified by exome sequencing an individual with partially overlapping features carrying a single *de novo* variant in *KIAA1109*. Although we cannot exclude that a second variant is present outside of the open reading frame, we might, alternatively, be dealing (1) with a spurious association or (2) with another syndrome related to AKS and associated with single variants in *KIAA1109*, similar to the *de novo* and biallelic variation recently associated with mitochondrial dynamics pathologies.[32] The high missense ExAC Z-score of

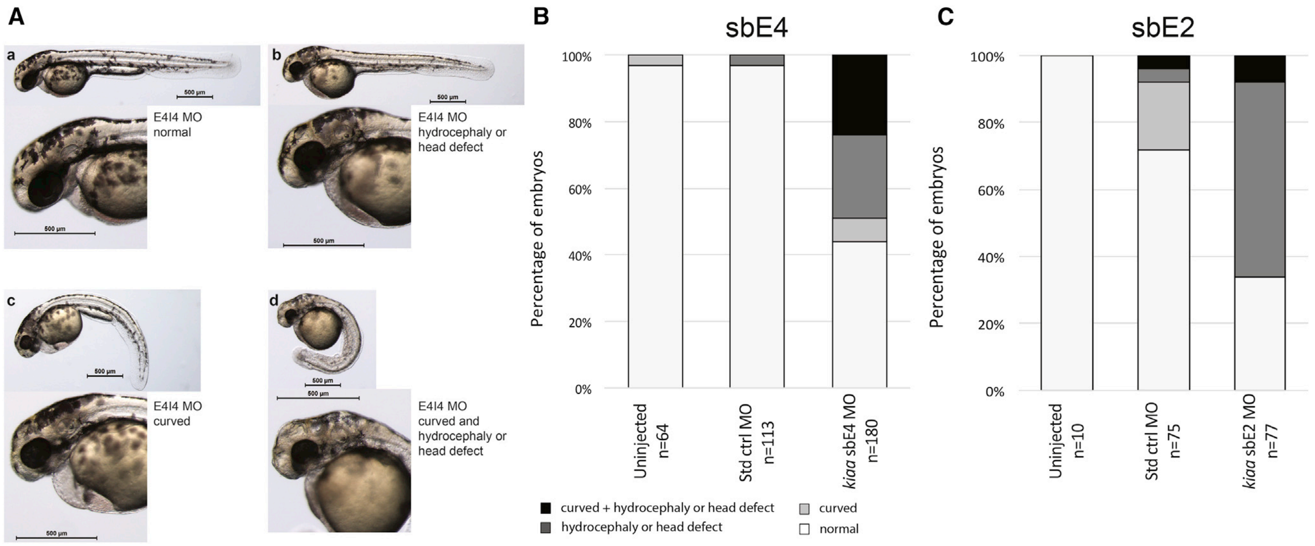

Figure 5. *kiaa1109* Knockdown in Zebrafish Results in Phenotypes Reminiscent of Probands' Clinical Features
(A) Lateral views representing the four classes of observed phenotypes in 2 dpf TU zebrafish embryos injected with sbE4-MO (morpholino) targeting *kiaa1109*: from top left to bottom right, normal, hydrocephalic or other head defects, curved and curved with head defect.
(B) Results for uninjected embryos (left) and those injected with equivalent amounts of standard control MO (center) or *kiaa1109* sbE4-MO (6.7 ng, right). Phenotyping and scoring were performed at 2 dpf in two independent experiments.
(C) Results for uninjected embryos (left) and those injected with equivalent amounts of standard control MO (center) or *kiaa1109* sbE2-MO (16.9 ng, right). Phenotyping and scoring were performed at 2 dpf in two independent experiments.

KIAA1109 is compatible with such hypothesis. The identification of other similarly affected individuals will allow disentangling this conundrum.

The observed combination of intellectual disability, corpus callosum hypoplasia, hydrocephalus, and talipes equinovarus is also reminiscent of the constellation of features seen in the *L1CAM*-associated (neural cell adhesion molecule L1 [MIM: 308840]) HSAS (hydrocephalus due to congenital stenosis of aqueduct of sylvius [MIM: 307000]) and CRASH (corpus callosum hypoplasia, retardation, adducted thumbs, spastic paraplegia, and hydrocephalus [MIM: 303350]) syndromes. Whereas HSAS syndrome leads to neonatal or infant death, *L1CAM* variants survivors are described as affected by CRASH syndrome. Similarly, ten of the herein described AKS-affected case subjects did not survive past infancy (16 if accounting for undiagnosed miscarriages), whereas the living UK.II.1, LT.II.1, and LT.II.2 individuals presented other prominent features reported in CRASH syndrome, such as adducted thumbs, short stature, microcephaly, language impairment, and abnormalities of tone. L1CAM is a cell adhesion molecule that plays critical roles in neuronal migration and differentiation.[33] Congenital joint contractures, limb deformities, hydrocephalus, corpus callosum agenesis, hypoplastic brainstem, cortical thinning, and high proportions of stillborn or neonatal death also are reminiscent of the PVHH (proliferative vasculopathy and hydranencephaly-hydrocephaly [MIM: 225790]) syndrome, a recessive disorder caused by variant in the transmembrane calcium transporter, *FLVCR2* (feline leukemia virus subgroup C receptor 2 [MIM: 610865]).

The *Drosophila* ortholog of *KIAA1109* named *tweek* is widely expressed but enriched in the brain lobes and in the ventral nerve cord.[27] Neuronal phosphatidylinositol-4,5-bisphosphate [PI(4,5)P(2)] levels are critical in restricting synaptic growth via localization and activation of presynaptic Wiscott-Aldrich syndrome protein/WASP, a phenomenon dependent on *tweek* but not on bone morphogenetic protein signaling.[34] The 5,005-amino-acid-long KIAA1109 protein is conserved from nematodes to vertebrates (Figure S8) in spite of a lack of recognizable domains, with the exception of a 22-residue amino-terminal transmembrane segment and a small central coiled-coil of 22 residues. It is described by specialists as an unconstrained peptide thought to adopt a definite conformation upon binding to its interactors.[35] Consistent with this hypothesis, multiple high-throughput protein-protein interactions screens coupling near-endogenous expression levels with quantitative proteomics and mass spectrometry have identified human or mouse KIAA1109 interactors. For example, CTNNB1 (catenin beta-1), a protein associated with a dominant form of intellectual disability (MIM: 615075), interacts with two separate regions of KIAA1109.[36] Another set of experiments showed high-confidence interactions with BUB3, DNAJB1, and PTPA, three proteins implicated in cell division.[37] BUB3 participates to the spindle-assembly checkpoint signaling and the establishment of kinetochore-microtubule attachments. It inhibits the ubiquitin ligase activity of the anaphase-promoting complex (APC/C) by phosphorylating its activator CDC2. PTPA, one of four major Ser/Thr phosphatases, negatively controls cell growth and division. DNAJB1 (a.k.a. HSP40)

interacts with HSP70 and stimulates its ATPase activity and its association with HIP. Interestingly, lower-confidence KIAA1109 protein interactors include BAG2 that competes with HIP for binding to the HSC70/HSP70 ATPase domain, as well as DRC1 and SMAD2. *DRC1* (MIM: 615288) encodes a central component of the nexin-dynein complex that regulates the assembly of ciliary dynein and is associated with primary ciliary dyskinesia (MIM: 615294). *SMAD2* (MIM: 601366) regulates cell proliferation, apoptosis, and differentiation through mediation of TGF-β signaling.

Conclusion

We propose that bi-allelic LoF and missense variants in *KIAA1109* cause an autosomal-recessive brain malformation disorder with cerebral parenchymal underdevelopment ranging from major cerebral parenchymal thinning with lissencephalic aspect to moderate parenchymal rarefaction, severe to mild ventriculomegaly, and cerebellar hypoplasia with brainstem dysgenesis, associated with club foot and arthrogryposis. Severe cases are incompatible with life. Although further studies have to be engaged, our findings suggest that *KIAA1109* is potentially involved in cell cycle control, particularly of the central nervous system.

Accession Numbers

Alkuraya-Kucinskas syndrome as described in this paper has been assigned MIM: 617822.

Supplemental Data

Supplemental Data include Supplemental Note, eight figures, and two tables and can be found with this article online at https://doi.org/10.1016/j.ajhg.2017.12.002.

Acknowledgments

We thank the affected individuals and their families for their contribution to this study. We are grateful to Keith Joung for reagents. This work was supported by grants from the Swiss National Science Foundation (31003A_160203) to A.R., the Lithuanian-Swiss cooperation program to reduce economic and social disparities within the enlarged European Union (CH-3-ŠMM-0104, Unigene project) to V.K. and A.R., and King Abdulaziz City for Science and Technology grant 13BIO-1113-20 to F.S.A. We also acknowledge the support of the Saudi Human Genome Program. The DDD Study presents independent research commissioned by the Health Innovation Challenge Fund (grant number HICF-1009-003), a parallel funding partnership between the Wellcome Trust and the Department of Health, and the Wellcome Trust Sanger Institute (grant number WT098051). The study has UK Research Ethics Committee approval (10/H0305/83, granted by the Cambridge South REC, and GEN/284/12 granted by the Republic of Ireland REC). The research team acknowledges the support of the National Institute for Health Research, through the Comprehensive Clinical Research Network. The funders had no role in study design, data collection and analysis, decision to publish, or preparation of the manuscript.

Received: December 2, 2016
Accepted: December 4, 2017
Published: December 28, 2017

Web Resources

1000 Genomes, http://browser.1000genomes.org/index.html
Burrows-Wheeler Aligner, http://bio-bwa.sourceforge.net/
dbSNP, https://www.ncbi.nlm.nih.gov/projects/SNP/
Ensembl genome assembly GRCh37, http://grch37.ensembl.org/Homo_sapiens/Info/Index
ExAC Browser, http://exac.broadinstitute.org/
ExomeDepth, https://cran.r-project.org/web/packages/ExomeDepth/index.html
GATK, https://software.broadinstitute.org/gatk/
GenBank, https://www.ncbi.nlm.nih.gov/genbank/
GTEx Portal, https://www.gtexportal.org/home/
NHLBI Exome Sequencing Project (ESP) Exome Variant Server, http://evs.gs.washington.edu/EVS/
OMIM, http://www.omim.org/
Picard, http://broadinstitute.github.io/picard/
PolyPhen-2, http://genetics.bwh.harvard.edu/pph2/
SIFT, http://sift.bii.a-star.edu.sg/
SnpEff, http://snpeff.sourceforge.net/
Splicing Finder, http://www.umd.be/HSF3/HSF.html
Swiss PDB Viewer, https://spdbv.vital-it.ch/

References

1. Yang, Y., Muzny, D.M., Reid, J.G., Bainbridge, M.N., Willis, A., Ward, P.A., Braxton, A., Beuten, J., Xia, F., Niu, Z., et al. (2013). Clinical whole-exome sequencing for the diagnosis of mendelian disorders. N. Engl. J. Med. *369*, 1502–1511.
2. Anazi, S., Maddirevula, S., Faqeih, E., Alsedairy, H., Alzahrani, F., Shamseldin, H.E., Patel, N., Hashem, M., Ibrahim, N., Abdulwahab, F., et al. (2017). Clinical genomics expands the morbid genome of intellectual disability and offers a high diagnostic yield. Mol. Psychiatry *22*, 615–624.
3. Vissers, L.E., Gilissen, C., and Veltman, J.A. (2016). Genetic studies in intellectual disability and related disorders. Nat. Rev. Genet. *17*, 9–18.
4. Mirzaa, G.M., and Paciorkowski, A.R. (2014). Introduction: Brain malformations. Am. J. Med. Genet. C. Semin. Med. Genet. *166C*, 117–123.
5. Kato, M. (2015). Genotype-phenotype correlation in neuronal migration disorders and cortical dysplasias. Front. Neurosci. *9*, 181.
6. Spalice, A., Parisi, P., Nicita, F., Pizzardi, G., Del Balzo, F., and Iannetti, P. (2009). Neuronal migration disorders: clinical, neuroradiologic and genetics aspects. Acta Paediatr. *98*, 421–433.
7. Verrotti, A., Spalice, A., Ursitti, F., Papetti, L., Mariani, R., Castronovo, A., Mastrangelo, M., and Iannetti, P. (2010). New trends in neuronal migration disorders. Eur. J. Paediatr. Neurol. *14*, 1–12.
8. Parrini, E., Conti, V., Dobyns, W.B., and Guerrini, R. (2016). Genetic basis of brain malformations. Mol. Syndromol. *7*, 220–233.
9. Graziano, C., Wischmeijer, A., Pippucci, T., Fusco, C., Diquigiovanni, C., Nõukas, M., Sauk, M., Kurg, A., Rivieri, F., Blau, N., et al. (2015). Syndromic intellectual disability: a new phenotype caused by an aromatic amino acid decarboxylase gene (DDC) variant. Gene *559*, 144–148.

10. Ng, P.C., and Henikoff, S. (2001). Predicting deleterious amino acid substitutions. Genome Res. *11*, 863–874.
11. Adzhubei, I.A., Schmidt, S., Peshkin, L., Ramensky, V.E., Gerasimova, A., Bork, P., Kondrashov, A.S., and Sunyaev, S.R. (2010). A method and server for predicting damaging missense mutations. Nat. Methods *7*, 248–249.
12. Salgado, D., Desvignes, J.P., Rai, G., Blanchard, A., Miltgen, M., Pinard, A., Lévy, N., Collod-Béroud, G., and Béroud, C. (2016). UMD-Predictor: a high-throughput sequencing compliant system for pathogenicity prediction of any human cDNA substitution. Hum. Mutat. *37*, 439–446.
13. Alazami, A.M., Patel, N., Shamseldin, H.E., Anazi, S., Al-Dosari, M.S., Alzahrani, F., Hijazi, H., Alshammari, M., Aldahmesh, M.A., Salih, M.A., et al. (2015). Accelerating novel candidate gene discovery in neurogenetic disorders via whole-exome sequencing of prescreened multiplex consanguineous families. Cell Rep. *10*, 148–161.
14. Xue, S., Maluenda, J., Marguet, F., Shboul, M., Quevarec, L., Bonnard, C., Ng, A.Y., Tohari, S., Tan, T.T., Kong, M.K., et al. (2017). Loss-of-function mutations in LGI4, a secreted ligand involved in Schwann cell myelination, are responsible for arthrogryposis multiplex congenita. Am. J. Hum. Genet. *100*, 659–665.
15. Schwarz, J.M., Cooper, D.N., Schuelke, M., and Seelow, D. (2014). MutationTaster2: mutation prediction for the deep-sequencing age. Nat. Methods *11*, 361–362.
16. Deciphering Developmental Disorders Study (2015). Large-scale discovery of novel genetic causes of developmental disorders. Nature *519*, 223–228.
17. Xue, Y., Ankala, A., Wilcox, W.R., and Hegde, M.R. (2015). Solving the molecular diagnostic testing conundrum for Mendelian disorders in the era of next-generation sequencing: single-gene, gene panel, or exome/genome sequencing. Genet. Med. *17*, 444–451.
18. Li, H., and Durbin, R. (2009). Fast and accurate short read alignment with Burrows-Wheeler transform. Bioinformatics *25*, 1754–1760.
19. Sander, J.D., Maeder, M.L., Reyon, D., Voytas, D.F., Joung, J.K., and Dobbs, D. (2010). ZiFiT (Zinc Finger Targeter): an updated zinc finger engineering tool. Nucleic Acids Res. *38*, W462–W468.
20. Harrow, J., Frankish, A., Gonzalez, J.M., Tapanari, E., Diekhans, M., Kokocinski, F., Aken, B.L., Barrell, D., Zadissa, A., Searle, S., et al. (2012). GENCODE: the reference human genome annotation for The ENCODE Project. Genome Res. *22*, 1760–1774.
21. Lek, M., Karczewski, K.J., Minikel, E.V., Samocha, K.E., Banks, E., Fennell, T., O'Donnell-Luria, A.H., Ware, J.S., Hill, A.J., Cummings, B.B., et al.; Exome Aggregation Consortium (2016). Analysis of protein-coding genetic variation in 60,706 humans. Nature *536*, 285–291.
22. Choi, Y., and Chan, A.P. (2015). PROVEAN web server: a tool to predict the functional effect of amino acid substitutions and indels. Bioinformatics *31*, 2745–2747.
23. Jordan, D.M., Frangakis, S.G., Golzio, C., Cassa, C.A., Kurtzberg, J., Davis, E.E., Sunyaev, S.R., Katsanis, N.; and Task Force for Neonatal Genomics (2015). Identification of cis-suppression of human disease mutations by comparative genomics. Nature *524*, 225–229.
24. Desmet, F.O., Hamroun, D., Lalande, M., Collod-Béroud, G., Claustres, M., and Béroud, C. (2009). Human Splicing Finder: an online bioinformatics tool to predict splicing signals. Nucleic Acids Res. *37*, e67.
25. Brown, S.D., and Moore, M.W. (2012). The International Mouse Phenotyping Consortium: past and future perspectives on mouse phenotyping. Mamm. Genome *23*, 632–640.
26. Skarnes, W.C., Rosen, B., West, A.P., Koutsourakis, M., Bushell, W., Iyer, V., Mujica, A.O., Thomas, M., Harrow, J., Cox, T., et al. (2011). A conditional knockout resource for the genome-wide study of mouse gene function. Nature *474*, 337–342.
27. Verstreken, P., Ohyama, T., Haueter, C., Habets, R.L., Lin, Y.Q., Swan, L.E., Ly, C.V., Venken, K.J., De Camilli, P., and Bellen, H.J. (2009). Tweek, an evolutionarily conserved protein, is required for synaptic vesicle recycling. Neuron *63*, 203–215.
28. Melé, M., Ferreira, P.G., Reverter, F., DeLuca, D.S., Monlong, J., Sammeth, M., Young, T.R., Goldmann, J.M., Pervouchine, D.D., Sullivan, T.J., et al.; GTEx Consortium (2015). Human genomics. The human transcriptome across tissues and individuals. Science *348*, 660–665.
29. Kok, F.O., Shin, M., Ni, C.W., Gupta, A., Grosse, A.S., van Impel, A., Kirchmaier, B.C., Peterson-Maduro, J., Kourkoulis, G., Male, I., et al. (2015). Reverse genetic screening reveals poor correlation between morpholino-induced and mutant phenotypes in zebrafish. Dev. Cell *32*, 97–108.
30. Aase, J.M., and Smith, D.W. (1968). Dysmorphogenesis of joints, brain, and palate: a new dominantly inherited syndrome. J. Pediatr. *73*, 606–609.
31. Patton, M.A., Sharma, A., and Winter, R.M. (1985). The Aase-Smith syndrome. Clin. Genet. *28*, 521–525.
32. Harel, T., Yoon, W.H., Garone, C., Gu, S., Coban-Akdemir, Z., Eldomery, M.K., Posey, J.E., Jhangiani, S.N., Rosenfeld, J.A., Cho, M.T., et al.; Baylor-Hopkins Center for Mendelian Genomics; and University of Washington Center for Mendelian Genomics (2016). Recurrent de novo and biallelic variation of ATAD3A, encoding a mitochondrial membrane protein, results in distinct neurological syndromes. Am. J. Hum. Genet. *99*, 831–845.
33. Itoh, K., and Fushiki, S. (2015). The role of L1cam in murine corticogenesis, and the pathogenesis of hydrocephalus. Pathol. Int. *65*, 58–66.
34. Khuong, T.M., Habets, R.L., Slabbaert, J.R., and Verstreken, P. (2010). WASP is activated by phosphatidylinositol-4,5-bisphosphate to restrict synapse growth in a pathway parallel to bone morphogenetic protein signaling. Proc. Natl. Acad. Sci. USA *107*, 17379–17384.
35. Toth-Petroczy, A., Palmedo, P., Ingraham, J., Hopf, T.A., Berger, B., Sander, C., and Marks, D.S. (2016). Structured states of disordered proteins from genomic sequences. Cell *167*, 158–170.e12.
36. Miyamoto-Sato, E., Fujimori, S., Ishizaka, M., Hirai, N., Masuoka, K., Saito, R., Ozawa, Y., Hino, K., Washio, T., Tomita, M., et al. (2010). A comprehensive resource of interacting protein regions for refining human transcription factor networks. PLoS ONE *5*, e9289.
37. Hein, M.Y., Hubner, N.C., Poser, I., Cox, J., Nagaraj, N., Toyoda, Y., Gak, I.A., Weisswange, I., Mansfeld, J., Buchholz, F., et al. (2015). A human interactome in three quantitative dimensions organized by stoichiometries and abundances. Cell *163*, 712–723.

The American Journal of Human Genetics, Volume *102*

Supplemental Data

KIAA1109 Variants Are Associated with a Severe Disorder of Brain Development and Arthrogryposis

Lucie Gueneau, Richard J. Fish, Hanan E. Shamseldin, Norine Voisin, Frédéric Tran Mau-Them, Egle Preiksaitiene, Glen R. Monroe, Angeline Lai, Audrey Putoux, Fabienne Allias, Qamariya Ambusaidi, Laima Ambrozaityte, Loreta Cimbalistienė, Julien Delafontaine, Nicolas Guex, Mais Hashem, Wesam Kurdi, Saumya Shekhar Jamuar, Lim J. Ying, Carine Bonnard, Tommaso Pippucci, Sylvain Pradervand, Bernd Roechert, Peter M. van Hasselt, Michaël Wiederkehr, Caroline F. Wright, DDD Study, Ioannis Xenarios, Gijs van Haaften, Charles Shaw-Smith, Erica M. Schindewolf, Marguerite Neerman-Arbez, Damien Sanlaville, Gaëtan Lesca, Laurent Guibaud, Bruno Reversade, Jamel Chelly, Vaidutis Kučinskas, Fowzan S. Alkuraya, and Alexandre Reymond

Supplemental note: case reports

Detailed description of affected individuals

LT Family:

The elder brother of the Lithuanian family LT.II.1 was born at 40 weeks of gestation. His birth weight was 4100 g (75 centile), his height was 55 cm (90 centile), and the Apgar score was 9 at 1 minute and 10 at 5 minute. Movements of the eyes in up direction were noted from 3 months of age. EEG was normal. At age of 13 months paroxysmal loss of consciousness with drooling, eyes rolling up, repetitive blinking, and muscle spasms had started. They lasted 3-10 minutes and occurred once a week or two. The proband was treated with valproic acid, nitrazepamum, diazepamum. Later lamotrigin and topiromate were added. The last paroxysm was at age of 3.5 years. Brain MRI at age of 8 years revealed small posterior fossa arachnoid cyst, discrete vermian atrophy and slight increase of the fluid-filled retro and infra-cerebellar space as well as mild enlargement of subarachnoid spaces of frontal regions. The boy has hypermetropia (+5.5), strabismus and astigmatism. Global psychomotor retardation and hypotonia were noted from two months of age. The proband could sit independently and walk for a few steps with support until age of 10 years. Later in life, his motor abilities declined and he could not stand without support. Spontaneous paroxysms of laughter manifested from 2 years of age. He shows stereotypic movements as arm flapping or waving, and rhythmic body rocking. Additional clinical features are delayed eruption of permanent teeth, enuresis and encopresis. At 13 years and 8 months his head circumference was 53 cm (10 centile), his weight 35 kg (3-10 centile) and height 154 cm (10-25 centile). He presented with muscular hypotonia, plagiocephaly, strabismus, fat deposit in pubis, mild contractures of large joints, hypermobile small joints, talipes valgus, partial cutaneous syndactyly of 2nd and 3rd toes and a hypoplastic scrotum. He has no speech and severe ID (IQ<35). Array-CGH with Agilent 44K revealed no potentially pathogenic genomic structural abnormalities.

The younger sister LT.II.2 was born from an uncomplicated pregnancy at 38 weeks of gestation. Her birth weight was 4200 g (90 centile), Apgar 9, 9. She had clubfoot and her hands and feet were in paretic position. Her affected brother showed a similar posture after birth. Myoclonic movements of arms have been noted few days after birth. Hypotonia, movements of the eyes in up direction, nystagmus and strabismus manifested during the first month. At age of 2 months brain CT and EEG

showed no abnormalities. Paroxysmal loss of consciousness with drooling, eyes rolling up, repetitive blinking, and muscle spasms manifested at age of 10 months. Brain MRI at age of 1 year 8 months showed enlargement of subarachnoid spaces facing discrete parenchymal rarefaction involving the frontal lobes. Hypermetropia (+6), strabismus and astigmatism were diagnosed by an ophthalmologist. Psychomotor retardation was noted from birth. She could roll over at age of 5 years, could never sit without support or stand. She has problems with developing the ability to chew food and has chronic constipation. She showed stereotypic movements as arm flapping or waving. She has excessive drooling and frequently grinds her teeth. At 7 years and 5 months her head circumference was 49 cm (3-10 centile), her weight 25 cm (50 centile) and her height 134 cm (90 centile). She presented with muscular hypotonia, plagiocephaly, strabismus, mild contractures of large joints, talipes valgus and psoriasis. She has no speech and severe ID (IQ<35). Array-CGH with Agilent 105K revealed no potentially pathogenic genomic structural abnormalities.

AL Family:

The AL family pregnancy was the first one of healthy consanguineous parents. It was marked by the discovery at 21 WA (weeks of amenorrhea) of multiple cerebral malformation including triventricular hydrocephalus and thalamic fusion and bilateral equinovarus foot by ultrasound, which resulted in decision to terminate the pregnancy. Measurements were in the normal range according to a 22 WA fetus, i.e. occipital frontal circumference (OFC) = 21cm (+2SD), weight= 478g, talus-vertex length = 27cm, except for the brain weight (44g <10th percentile). A thorough examination of the AL.II.1 fetus revealed an arthrogryposis of the upper and lower limbs with crisped hands, adductus thumbs and bilateral equinovarus feet. The facial dysmorphism encompassed hypertelorism, a big horizontal mouth with retrognathism and miss-oriented ears. A scrotal hypoplasia and choane atresia was found. A slightly diffuse sero-hemorragic effusion was noticed. Neuropathological examination demonstrated, on the supra-tentorial space, both absence of cortical lamination and diffuse migration anomalies within a thin parenchymal mantle, as well as ventriculomegaly and voluminous germinal matrix. Corpus callosum was not identified. On infra-tentorial space, pathological findings include hypoplasia of the pons with absence of the longitudinal and transversal fibers and dysplasia of the

cerebellum characterized by lack of foliation and poorly identified vermis as well as narrowing of the aqueduct. Note that gyration analysis could not be performed due to poor conservation. Other tissues analysis revealed a dilatation of lymph vessels in the fibrous septa of the lung suggestive of a pulmonary lymphangiectasia and bilateral cataract with crystalline fibers of variable size and orientation. Skeletal X-rays examinations were normal. Standard blood chromosomes and a 60K quator PréCytoNEMv2 array-CGH (Agilent Technologies, CA, USA) were normal.

TU1 family:

The proband TU1.II.1 was the first child of first cousins parents from Tunisia. While her mother is healthy, her father is unable to read and write. He is epileptic with cognitive impairment secondary to encephalitis occurring at the age of five years. His MRI performed at 27 years was normal. During pregnancy, hypoplastic left heart, arthrogryposis with club feet and cerebral abnormalities were found. Prenatal imaging (ultrasound and MRI) showed on the infra-tentorial space cerebellar hypoplasia and brainstem dysgenesis characterized by flat and elongated pons and slightly kinked brainstem with increased fluid-filled retro-cerebellar spaces. Supra-tentorial anomalies include severe parenchymal (or cerebral mantle) thinning with major lack of gyration (lissencephalic aspect) associated with voluminous germinal matrix protruding within moderate ventriculomegaly and absence of corpus callosum. Cephalic biometry was normal. She was born at 34 GW. She presented facial dysmorphism with hypotelorism, deep palate, long fingers and left club foot. Cardiac ultrasound confirmed left hypoplastic heart with mitral and aortic atresia. Cerebral ultrasound confirmed antenatal ascertainments. Karyotype and FISH 22q11.2 were normal. She died at 3 days of life. Pathological examination was not performed according to parental decision.

This first pregnancy was followed by two spontaneous miscarriages (TU1.II.2 and TU1.II.3). During the fourth pregnancy of this couple, the mother was referred due to recurrence of a polymalformative fetus (TU1.II.4) characterized on ultrasound examination at 22 weeks by severe parenchymal thinning with lack of gyration associated with ventriculomegaly and corpus callosum agenesis. Extra-cerebral findings included arthrogryposis but a normal cardiac anatomy. The pregnancy was terminated at 23 GW. Pathological examination showed clenched hands with bilateral

camptodactyly, bilateral clubfeet, shoulder and hip joints contractures. No visceral malformation was observed. Brain examination showed a complete agenesis of the corpus callosum, ventricular dilatation, severe cortical malformations with a reduced cortical plate and vermian agenesis. Hyperplastic germinal matrix was protruding within ventricles. Neuropathological examination showed dysplasia of brainstem and cerebellum with neuroglial ectopia. At the supra-tentorial level, no callosal fibers were identified. The cortical plate showed neuronal depletion, numerous foci of heterotopia were observed within white matter. The three next pregnancies of the couple resulted in three healthy children.

TU2 family:

TU2.II.2 was the second child of consanguineous parents from Tunisia with no known genealogical links with the TU1 family. Prenatal imaging (ultrasound and MRI) showed on the infra-tentorial space both cerebellar hypoplasia and dysgenesis associated to severe brainstem dysgenesis characterized by flat and elongated pons and slightly kinked brainstem with increased fluid-filled retro-cerebellar spaces. Corpus callosum could not be identified. Supra-tentorial anomalies include severe parenchymal thinning with major lack of gyration demonstrating a pseudo-lissencephalic aspect as well as voluminous germinal matrix protruding within severe ventriculomegaly. Moreover, microphthalmia and club feet were present. She was born at 37 GW with a birth weight of 3100g, birth length of 45 cm and head circumference of 33 cm. She presented with microphthalmia, blepharophimosis, narrow chest, club feet and hands. She was hypotonic, with a reduced mobility and feeding and sucking difficulties. Post-natal MR confirmed prenatal data showing both thin cortical ribbon and global parenchymal thinning with lissencephalic aspect as well as bands of gray matter situated between the lateral ventricle and cerebral cortex, suggestive of neuronal migration disorder. This examination demonstrated also multiple germinolytic cysts within bilateral voluminous residual germinal matrix protruding in a severe dilated ventricular system. On infra-tentorial space, cerebellar and brainstem dysgenesis were confirmed associated with increased fluid-filled retro-cerebellar spaces and showed also a large arachnoid cyst responsible for mass effect on the distal part of the cerebellar tentorium. She died at 12 days of life, secondary to hypoventilation in a context of pulmonary hypoplasia. Autopsy was not performed according to parental decision. High-throughput sequencing of a panel of

29 genes involved in cortical malformation failed to pinpoint possibly causative variants.

UK family:

The British proband UK.II.1 (DDD #263241) is a 11 year old girl. She presented global developmental delay, behavioral problems (poor concentration, immaturity and minor self-harm when angry or frustrated) with mild to moderate learning disability allowing her to be in mainstream school but 2 years behind her peers. Prenatal imaging, including MRI, showed major microcephaly (HC -5 SD) with reduced white matter volume and mild ventriculomegaly but no abnormality of the cerebral hemispheres and no midline abnormalities. There was hypertelorism, slightly upslanting palpable fissures and oculo-motor apraxia, hypermetropia and strabismus. Dental crowding and high palate were also noticed. Skeletal abnormalities were also observed in this proband such as asymmetry of the thorax, mild bilateral talipes managed by physiotherapy, syndactyly of the 2nd and 3rd toes, 5th toe clinodactyly and hallux valgus. Finally, she had a complex congenital heart disorder with tetralogy of Fallot and pulmonary atresia, along with gastroesophageal reflux.

SA1 family:

The detailed phenotype of this individual was described in reference 13. Briefly, the SA1.II.1 fetus had severe ventriculomegaly with supra-tentorial cerebral mantle thinning associated with cerebellar hypoplasia, pleural effusion, severe arthrogryposis (fixed elbows, fixed bilateral talipes, bilateral overlapping fingers, bilateral clinodactyly, and bilateral club foot), low set ears, small eyes and micrognathia. The baby died after around one hour of delivery in the NICU. Parents are first cousins and they have had a previously affected child who is now deceased.

SA2 family:

First cousin Saudi parents presented to King Faisal Specialist Hospital & Research Center (KFSHRC) Maternal-Fetal Medicine for further evaluation when a screening ultrasound at a local hospital revealed multiple fetal anomalies of SA2.II.1. Ultrasonographic assessment at 18 weeks revealed hydrocephalus with hypoplastic cerebellum, absent corpus callosum, and shortened upper and lower limb bones. Multiple flexion deformities were noted. Heart could not be visualized but the chest,

abdomen and face appeared normal. No fetal movements were detected. Delivery was induced and resulted in a stillbirth with severe hydrocephalus and arthrogryposis multiplex. Babygram revealed poorly ossified calvarium and mildly shortened long bones. Parents did not authorize clinical photographs or autopsy.

SA3 family:

First cousin Saudi parents presented to KFSHRC Maternal-Fetal Medicine because of two previous intrauterine fetal deaths diagnosed with severe hydrocephalus and multiple skeletal anomalies (no records available). Ultrasonographic assessment of SA3.II.1 at 30 weeks revealed intrauterine fetal death with absent heart activity. Hydrocephalus, hypoplastic cerebellum, multiple flexion deformities and skin edema were noticed. Labor was induced and a dead fetus was delivered with severe hydrocephalus and arthrogryposis. The family declined clinical photographs or autopsy.

US Family:

The US family pregnancy was the third of healthy non-consanguineous parents. The couple's first pregnancy (US.II.1) resulted in a spontaneous miscarriage of unknown etiology at 11 weeks, cytogenetic testing revealed a normal 46, XY karyotype. The couple's second pregnancy (US.II.2) resulted in a spontaneous miscarriage at 20 weeks, secondary to findings of cystic hygroma, hydrocephaly, clinodactyly and talipes. Cytogenetic testing revealed 46, XY karyotype. No other testing was performed. The US.II.3 affected fetus was evaluated at 18 weeks 5 days gestation for concern for multiple congenital anomalies including cystic hygroma, ventriculomegaly, echogenic bowel, low conus and closed spinal defect with splayed vertebral arches at L4-L5, small liver only omphalocele, non-immune hydrops with scalp edema, bilateral pleural effusions, possible anal atresia, talipes, hyperflexed wrists, bilateral clinodactyly, low-set ears, short penis with bulbous shaft identified by ultrasound. High resolution fetal MRI detected ventriculomegaly, narrowing of the frontal lateral ventricles, thin cortical mantle, abnormal hypo-intense basal ganglia, absent corpus callosum and 3rd ventricle, kinked brainstem, hypoplasia or the cerebral hemispheres, and arthrogryposis. Fetal echocardiogram revealed a coarctation of the aorta. Additionally, during the evaluation the fetus was exhibiting

abnormal umbilical artery dopplers showing decreased end diastolic flow. The family elected for pregnancy termination and autopsy. A thorough neurological and genetic autopsy was completed. Physical examination detected severe arthrogryposis with flexion contractures and pterygia formation in all joints of the extremities as well as muscle atrophy, webbed neck, partially malrotated bowel and small placenta. Evaluation of the brain was significant for hydrocephaly, diaphanous pallium, small cerebellum and brainstem, heterotopic grey tissue, thickened basal meninges, all concerning for a migration defect and brainstem hypoplasia. Prenatal genetic testing included a normal 46, XY male karyotype via chorionic villus sampling.

SG Family:

The Singaporean siblings were two sons born to healthy, unrelated parents. The elder SG.II.1 brother was born at 34 weeks of gestation, the product of the couple's first pregnancy. Prenatally, severe ventriculomegaly was detected on ultrasound scan and fetal magnetic resonance imaging (MRI). Prenatal genetic testing included a normal male karyotype (46,XY) via amniocentesis. At birth, his weight was 2425 g (50-90th centile), his length was 43 cm (10-50th centile) and his head circumference was 38 cm (4 cm > 97th centile). He had minimal respiratory effort at birth and required immediate intubation and mechanical ventilation. On examination, macrocephaly, hypertelorism, posteriorly rotated ears, flattened nasal bridge, and excess skin fold of the neck were observed. He had generalized arthrogryposis, involving bilateral shoulders, elbows, wrists, hands and knees. He also had bilateral structural congenital talipes equinovarus (CTEV). Eye examination showed bilateral congenital cataracts and microphthalmia. He also had hypotonia, an ano-rectal malformation with recto-perianal fistula, and a small atrial septal defect/patent foramen ovale. Cerebral MR demonstrated severe ventriculomegaly with decreased pericerebral spaces, severe parenchymal thinning and smooth cortical surface, germinolysis cysts involving voluminous germinal matrix protruding within lateral ventricles as well as non identification of corpus callosum. Infra-tentorial findings include severe cerebellar hypoplasia with severe brain-stem dysgenesis characterized by a kinking aspect. Array-CGH with Agilent 44K revealed no potentially pathogenic genomic structural abnormalities. A ventriculo-peritoneal shunt was inserted at 2 months of age. He did not have adequate spontaneous respiration and was ventilator-dependent from birth, apart from two days when a trial of

extubation was done. He passed away at 3 months of age, from pneumonia and septic shock.

The next two pregnancies of the parents resulted in miscarriages (SG.II.2 and SG.II.3). Their fourth pregnancy resulted in the birth of the SG.II.4 younger brother, also at 34 weeks of gestation. Antenatally, bilateral ventriculomegaly, and club feet and hands were observed on ultrasound scan. At birth, his weight was 2130 g (10-50th centile), his length was 41 cm (10th centile) and his head circumference was 38.5 cm (4.5cm > 97th centile). His clinical findings were remarkably similar to his elder brother, with similar facial features (hypertelorism, bilateral low-set ears, short nose, anteverted nares), webbed neck, generalized arthrogryposis (involving bilateral elbows, wrists, hips and knees), bilateral structural congenital talipes equinovarus (CTEV), bilateral congenital cataracts and microphthalmia, and hypotonia. However, the younger brother did not have an ano-rectal malformation, and his cardiac defect was a fenestrated atrial septal defect. Magnetic resonance imaging (MRI) of the brain showed imaging findings similar to those that were described on the index case. He also remained ventilator-dependent from birth. The parents decided on withdrawal of invasive ventilation, and the baby passed away at 1 month of age.

Figure S1

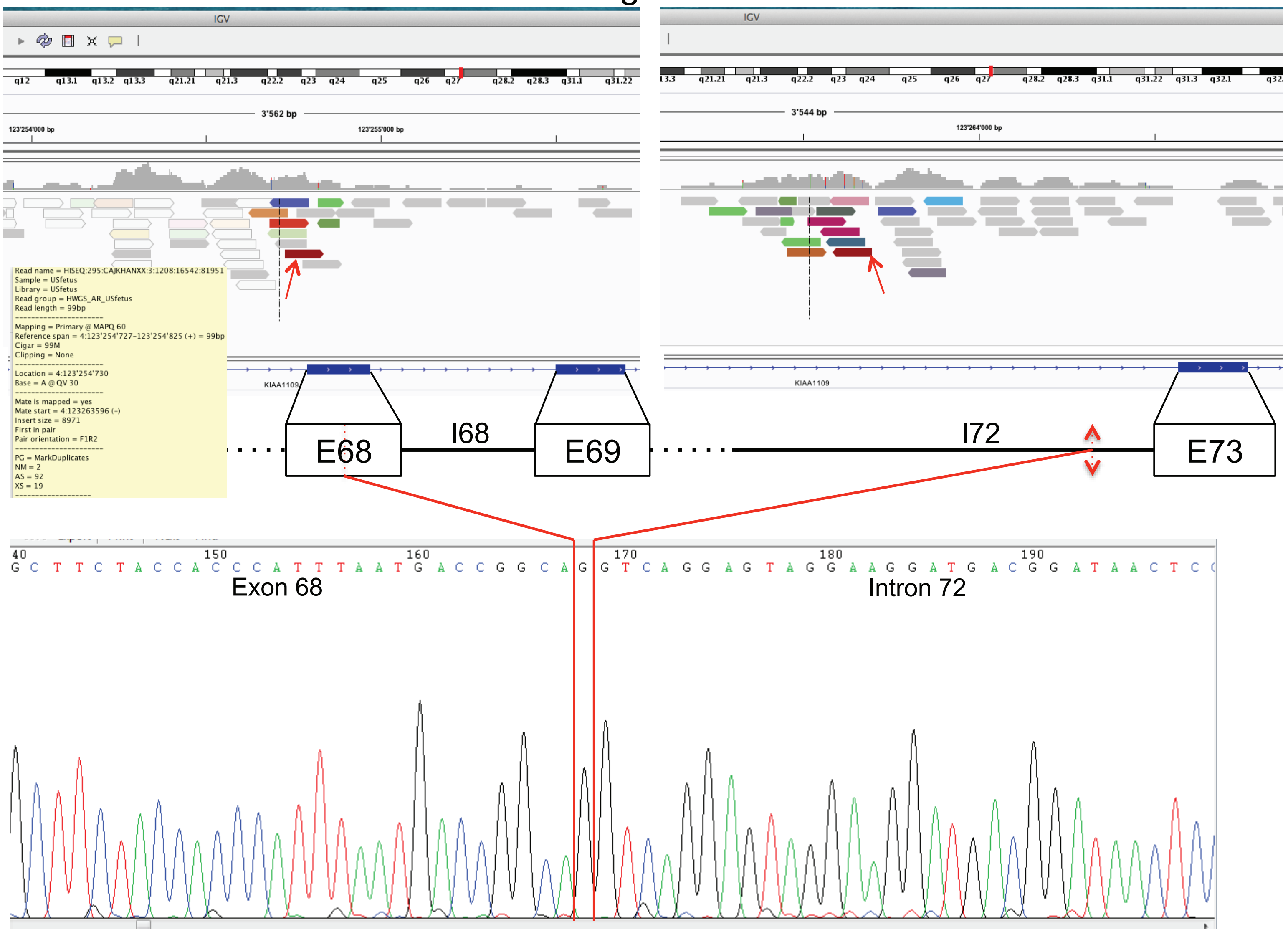
IGV
3'562 bp
123'254'000 bp
123'255'000 bp
Read name = HISEQ:295:CAJKHANXX:3:1208:16542:81951
Sample = USfetus
Library = USfetus
Read group = HWGS_AR_USfetus
Read length = 99bp
Mapping = Primary @ MAPQ 60
Reference span = 4:123'254'727-123'254'825 (+) = 99bp
Cigar = 99M
Clipping = None
Location = 4:123'254'730
Base = A @ QV 30
Mate is mapped = yes
Mate start = 4:123263596 (-)
Insert size = 8971
First in pair
Pair orientation = F1R2
PG = MarkDuplicates
NM = 2
AS = 92
XS = 19
KIAA1109
3'544 bp
123'264'000 bp
KIAA1109
E68
I68
E69
I72
E73
Exon 68
Intron 72

Figure S1: Breakpoint mapping of the US family large deletion

From top to bottom: whole genome paired-end sequences of the US.II.3 fetus are visualized on the Integrative Genomics Viewer (IGV). Reads aligning to *KIAA1109* intron 67, exon 68 (E68), intron 68 (I68), exon 69 (E69) (left panel), intron 72 (I72) and exon 73 (E73) (right panel) are schematically shown. A single pair of reads (chestnut brown pointed-head rectangles pinpointed by red arrows) mapping unequivocally 8971 bp apart was identified (see yellow inset) allowing to narrow down the mapping of the breakpoints of the paternally-inherited deletion of the US.II.3 proband. They were then finely mapped using PCR and Sanger sequencing to coordinates chr4:123,254,885 (hg19) within E68 and chr4:123,263,438 within I72 respectively with the insertion of a guanine nucleotide. The breakpoints are depicted by a dotted line in E68, a double pointed arrow in I72 and two vertical red lines on each side of the inserted G nucleotide in the Sanger chromatogram, respectively.

Figure S2

Exon1
chr13:12,668,517-12,668,866

agtgtgcatttgcagtttacttgataagtgagaggctgatgcagaccctgtgtaatttgatagtgatgtccgctctgt
ttatctgcagtggggtgtaaccATGGATAAAGGCAACAACAGTCTCCCCACTTACGATGAAATAGATGAATACCTCAG
CAGGCGCAACTCCACCTTCGTGTGGCTCCTTGTGGgtaagtgatttatgatttgtgcttcgtctacactttgcaatca
gcacagttacagtctacactggaggtgcctagtagattgcattgagttttaaaggtacagttcacccaaaaatgaacc
ttttactgaacttacataaacctgtatgtgtcctcctt

Exon 4
chr13:12,671,867-12,672,216

acacacacacacacaaacacacgcaaataaacttttgagactctgcttattgctgttgtctgtgagaccttttttcatt
tgatatgggttatatttcatctttatttactctgttgtttatatttgtcattttcagAATTCAGGATGGGTTACTCAT
ATTTCGTTGGTGGAAGATGTACAACCCAAAGCAGAAGCAACATGgtcagtgcaaaaacagaacaaacagcaaagaatt
gttcaatttgagcacatcatgtcagtctcaatagtctcagtctcagtacaccaatcccacaatatctgtaaatgcttg
tcattttagttgctctatggttgggtttatctgtgctt

Exon 7
chr13:12,683,417-12,683,816

aattgaatgtcttatttctatgagtgtcctatcctcaagtattgtgaccaaagaaaaacctaataaatattgactgac
attactctttctggttttgccttcagGGTCGCGTAGCATTTGGTAATCACCATCTTCCTCAGACCCTCTGCATGAACT
TTGACGATGCCTTCTTGACATATGCCACCAAACCACCCAGCAGCCATCTGGATCAGTTCATGCACATTGTGAAGGGTT
CATTGGAGAACGTTCGTGTCATGCTGGTGCCCAGTCCACGATACCTAGGCCTTCAGAATGACGAgtgagtcaaataat
ctccagtatatcagattagattagattctactttattgtcattgggcaggaatagagccatttaaaggatattcacc
aaaaaattac

Figure S2: Zebrafish *kiaa1109* editing target sites

CRISPR Cas9 target sites in exons 1, 4 and 7 of zebrafish *kiaa1109* are shown. Exon numbering and chromosome 13 coordinates according to NM_001145584.1 and zebrafish genome assembly GRCz10/danRer10, respectively. Exon sequences are in blue and upper case, nucleotides at exon edges are in light blue. sgRNA target sites are underlined with PAM sequences boxed. The expected position for Cas9 DNA cleavage and mutations introduced by non-homologous end joining is between the two red nucleotides. The position for annealing of genotyping oligonucleotides is highlighted yellow; with the reverse oligonucleotide having the reverse complement of the second highlighted sequence (one DNA strand is represented).

Figure S3

A. Exon 1

WT

del TCGT

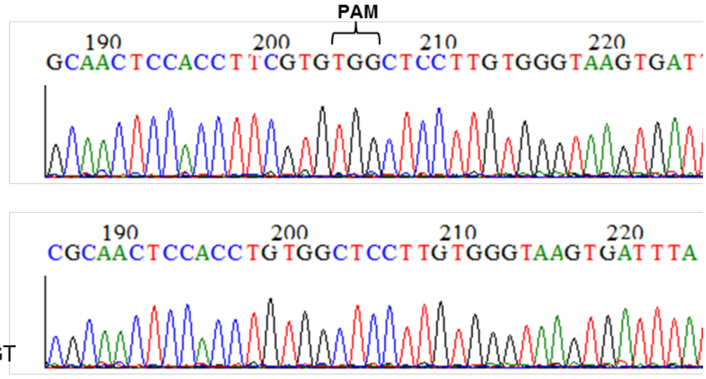

Reference sequence from exons 1 and 2.
Exon border nucleotides in blue, PAM in red

```
atggataaaggcaacaacagtctccccacttacgatgaaatagatgaatacctcagcagg
 M   D   K   G   N   N   S   L   P   T   Y   D   E   I   D   E   Y   L   S   R
cgcaactccaccttcgtgtggctccttgtggccactattatgtcatgcggatggatcata
 R   N   S   T   F   V   W   L   L   V   A   T   I   M   S   C   G   W   I   I
tatctgacatactacaattctcgcaatattggcctcgtcctcaccctcatcattaaccga
 Y   L   T   Y   Y   N   S   R   N   I   G   L   V   L   T   L   I   I   N   R
ctctacaagaatggatacatacacatcg
 L   Y   K   N   G   Y   I   H   I
```

Mutation in exon 1 (deletion of TCGT), NM_001145584.1:c.74_77del

```
atggataaaggcaacaacagtctccccacttacgatgaaatagatgaatacctcagcagg
 M   D   K   G   N   N   S   L   P   T   Y   D   E   I   D   E   Y   L   S   R
cgcaactccacctgtggctccttgtggccactattatgtcatgcggatggatcatatatc
 R   N   S   T   C   G   S   L   W   P   L   L   C   H   A   D   G   S   Y   I
tgacatactacaattctcgcaatattggcctcgtcctcaccctcatcattaaccgactct
 -   H   T   T   I   L   A   I   L   A   S   S   S   P   S   S   L   T   D   S
acaagaatggatacatacacatcg
 T   R   M   D   T   Y   T   S
```

B. Exon 4

WT

delT insGTAAGATGTAA

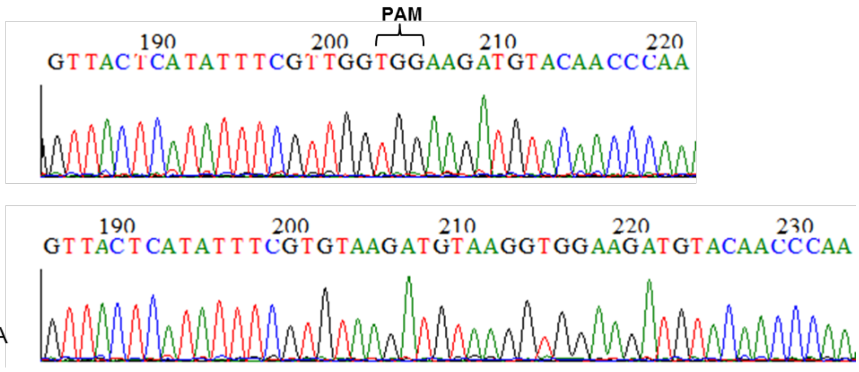

Reference sequence from exons 4 and 5.
Exon border nucleotides in blue, PAM in red

```
aattcaggatgggttactcatatttcgttggtggaagatgtacaacccaaagcagaagcaa
  I  Q  D  G  L  L  I  F  R  W  W  K  M  Y  N  P  K  Q  K  Q
catgacccgaaggctgagacgcgtctctatgttactgttaacggctttgagttccatgtt
 H  D  P  K  A  E  T  R  L  Y  V  T  V  N  G  F  E  F  H  V
tataatcggacggatctgtacactcggcttcaggaaatatttggcctcgagcccacccttt
 Y  N  R  T  D  L  Y  T  R  L  Q  E  I  F  G  L  E  P  T  L
atccaatccaaccgggatgaggagaaaggccgagaacagagggataaatccttggagag
 I  Q  S  N  R  D  E  E  K  G  R  E  Q  R  D  K  S  L  E
```

Mutation in exon 4 (indel, delT insGTAAGATGTAA),
NM_001145584.1:c.316delTinsGTAAGATGTAA

```
aattcaggatgggttactcatatttcgtgtaagatgtaaggtggaagatgtacaacccaaa
  I  Q  D  G  L  L  I  F  R  V  R  C  K  V  E  D  V  Q  P  K
gcagaagcaacatgacccgaaggctgagacgcgtctctatgttactgttaacggctttga
 A  E  A  T  -  P  E  G  -  D  A  S  L  C  Y  C  -  R  L  -
gttccatgtttataatcggacggatctgtacactcggcttcaggaaatatttggcctcga
 V  P  C  L  -  S  D  G  S  V  H  S  A  S  G  N  I  W  P  R
gcccacccttatccaatccaaccgggatgaggagaaaggccgagaacagagggataaatc
 A  H  P  Y  P  I  Q  P  G  -  G  E  R  P  R  T  E  G  -  I
cttggagag
 L  G  E
```

C. Exon 7

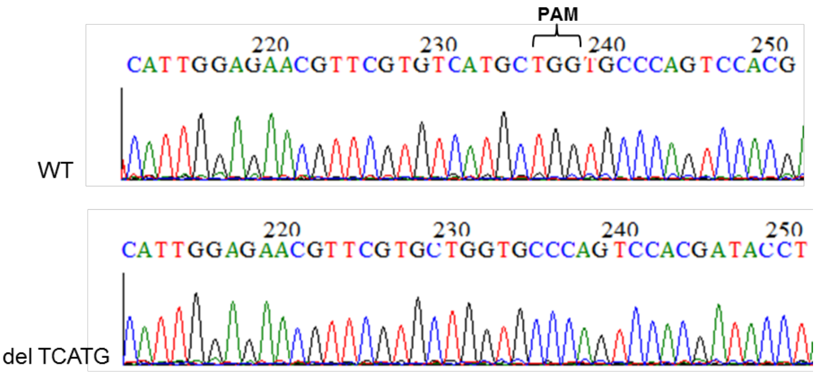

Reference sequence from exons 7 and 8.
Exon border nucleotides in blue, PAM in red

```
ggtcgcgtagcatttggtaatcaccatcttcctcagaccctctgcatgaactttgacgat
 G  R  V  A  F  G  N  H  H  L  P  Q  T  L  C  M  N  F  D  D
gccttcttgacatatgccaccaaaccacccagcagccatctggatcagttcatgcacatt
 A  F  L  T  Y  A  T  K  P  P  S  S  H  L  D  Q  F  M  H  I
gtgaagggttcattggagaacgttcgtgtcatgctggtgcccagtccacgatacctaggc
 V  K  G  S  L  E  N  V  R  V  M  L  V  P  S  P  R  Y  L  G
cttcagaatgacgaacctccgaggctcatgggtgagggatttgtggtcatgcagtcgaat
 L  Q  N  D  E  P  P  R  L  M  G  E  G  F  V  V  M  Q  S  N
gatgtggacatttactactatcaagatgaaccag
 D  V  D  I  Y  Y  Y  Q  D  E  P
```

Mutation in exon 7 (del TCATG), NM_001145584.1:c.758_762del

```
ggtcgcgtagcatttggtaatcaccatcttcctcagaccctctgcatgaactttgacgat
 G  R  V  A  F  G  N  H  H  L  P  Q  T  L  C  M  N  F  D  D
gccttcttgacatatgccaccaaaccacccagcagccatctggatcagttcatgcacatt
 A  F  L  T  Y  A  T  K  P  P  S  S  H  L  D  Q  F  M  H  I
gtgaagggttcattggagaacgttcgtgctggtgcccagtccacgatacctaggccttca
 V  K  G  S  L  E  N  V  R  A  G  A  Q  S  T  I  P  R  P  S
gaatgacgaacctccgaggctcatgggtgagggatttgtggtcatgcagtcgaatgatgt
 E  -  R  T  S  E  A  H  G  -  G  I  C  G  H  A  V  E  -  C
ggacatttactactatcaagatgaaccag
 G  H  L  L  S  R  -  T
```

Figure S3: Zebrafish *kiaa1109* variants

We generated three different stable zebrafish lines with frameshift variants, i.e. c.74_77del, c.316delTinsGTAAGATGTAA and c.758_762del in exons 1 **(A)**, 4 **(B)** and 7 **(C)** of *kiaa1109* (a.k.a. si:ch211-233a24.2, exon numbering and variant nomenclature according to NM_001145584.1). Representative sequencing chromatograms are shown for unedited (WT allele, top) and edited (mutant allele, bottom) sequences. The traces are from clones from genotyping PCRs, using tail clips of heterozygous adult fish. The predicted frameshift resulting from these mutations is given as a block of cDNA, with its single letter translated amino acid sequence under the DNA, in the regions of the editing target sites. Predicted stop codons are shown as hyphens.

Figure S4

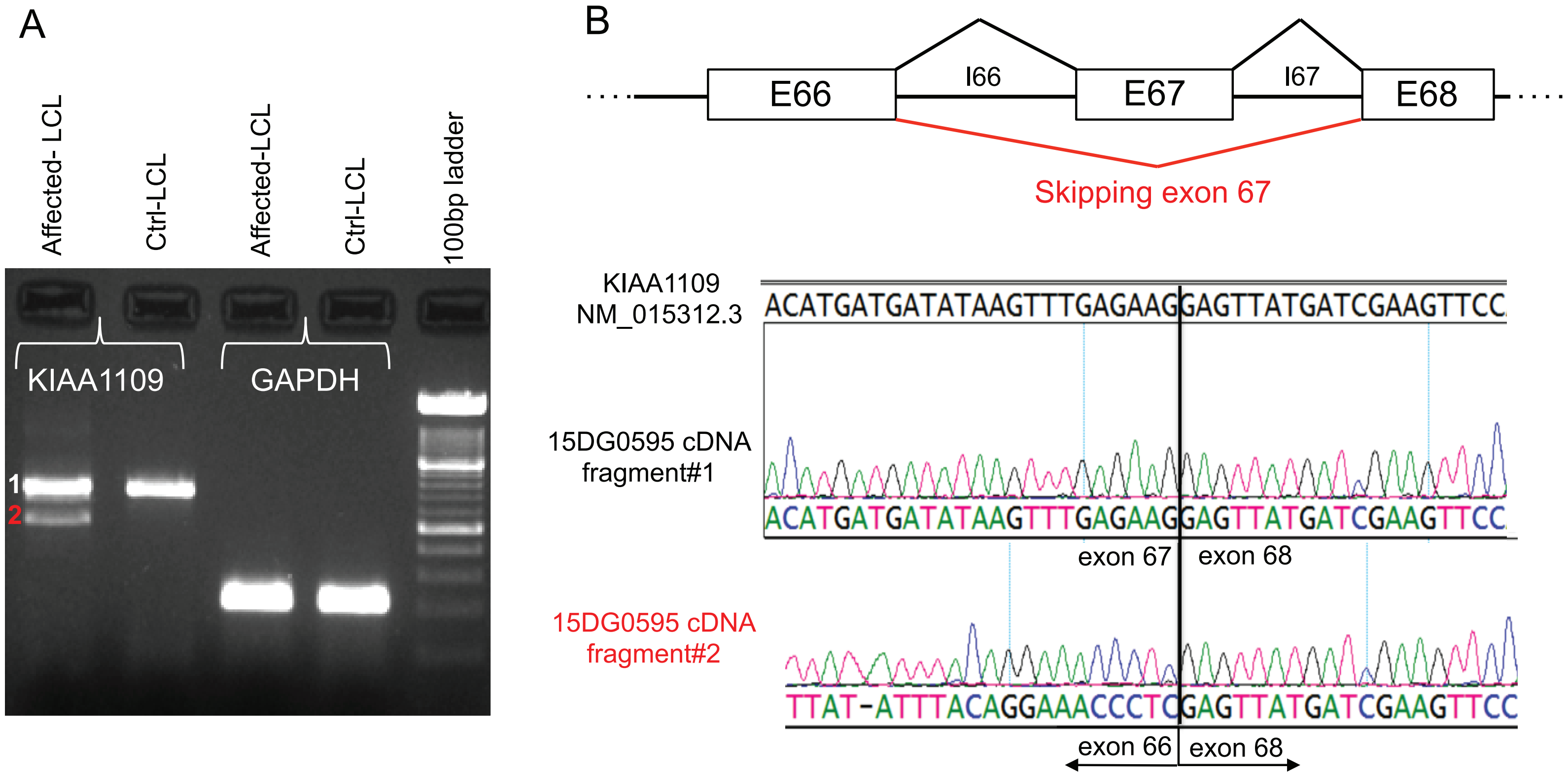
A
Affected- LCL
Ctrl-LCL
Affected-LCL
Ctrl-LCL
100bp ladder
KIAA1109
GAPDH
1
2
B
E66
I66
E67
I67
E68
Skipping exon 67
KIAA1109
NM_015312.3
ACATGATGATATAAGTTTGAGAAGGAGTTATGATCGAAGTTCC
15DG0595 cDNA
fragment#1
ACATGATGATATAAGTTTGAGAAGGAGTTATGATCGAAGTTCC
exon 67
exon 68
15DG0595 cDNA
fragment#2
TTAT-ATTTACAGGAAACCCTCGAGTTATGATCGAAGTTCC
exon 66
exon 68

Figure S4: The *KIAA1109* c.11250-1G>A variant induces skipping of exon 67 in the SA2.II.1 fetus.

(A) Agarose gel separation of RT-PCR amplicons of *KIAA1109* exons 66 to 68 from lymphoblastoid cell lines (LCL) from the affected SA2.II.1 fetus (affected-LCL) and a control individual (Crtl-LCL) (left). We observe two bands corresponding to an amplicon with all exons (fragment #1) and an amplicon missing exon 67 (2) specifically in the LCLs of the affected fetus. *GAPDH* was used as positive control (right). **(B)** Sanger sequencing of fragment 1 (top) and 2 (bottom) amplicons from the affected-LCL compared with that of the Ctrl-LCL amplicon. Sanger sequencing of fragment 2 showed the skipping of exon 67.

Figure S5

```
                                      R968C
Human        --------FHVVCREYELERPKSVIICQHGIDRRFCESKLSCIPGPCPTSDDLKYTMIRL 995
Pig          --------FHVVCREYELERPKSVIVCQHGIDRRFCESKLSCIPGPCPTSDDLKYTMTRL 994
Mouse        --------FHVVCREYELERPKSVIVCQHGIDRRFCESKLSCIPGPCPTSDDLKYTMTRL 995
Opossum      --------FHVVCREFELERPKSVILCQHGIDRRFCESKLSCVPGPCSTSDELKYTMTRL 1000
Chicken      --------FHVVCREFELERPKSVIICQHGIDRRFCDSKMNCVPGPCPTSDDLKYTMTRL 994
Coelacanth   STVLFCHLLFSSCREFIEKSI----------------KQICCLLPCEP--EVRYTFCYL 866
Zebrafish    --------FHVMSREFQLEQPKPSVTCQHGVDRRICDAKHAGLPGHCRTSEDLKYTMTRL 977
                     :.  .**:  :                  *        *     :::**:  *
                                             Y1329C
Human        TQDKSVGQSPLRSPLKRQASVCSTRLGSTKSLTAAFYGDKQPVTVGVQFSSDVSRSDENV 1352
Pig          TQDKSVGQSPLRSPLKRQASVCSTRLGSTKSLTAAFYGDKQPVTVGVQFSSDVSRSDENV 1351
Mouse        TQDKSVGQSPLRSPLKRQASVCSTRLGSTKSLTAAFYGDKQPVTVGVQFSSDVSRSDENV 1352
Opossum      TQDKSGVQSPLRSPLKRQASVCSTRLGSTKSLTAAFYGDKQPVTVGVQFSSDVSRSDENV 1357
Chicken      SQERPVGQSPMRSPLKRQASVCSTRLGSTKSLTAAFYGDKQPVPVGVQFSSDVSRSDENV 1350
Coelacanth   SQDKPSVHSPLKSPLKRQASVCSTRLGSTKSLTAAFFGEKQPAPAGVQFSSEVSRSDENV 1218
Zebrafish    IPEGPPLRSPLRSPLKRQSSVQSARLGSTKSLSAAVFVEKALPPAGVQFSSEVSRSDENV 1307
               :     :**::******:**  *:*********:**.: :*     .******:********
                                                     M1573I
Human        SLHRPLDLDTPTSEESSSSFEQLSVPTFKVIKQGLTANSLLDRGMQLSGSTSNTPYTPLE 1588
Pig          SLHRPLDLDTPTSEESSSSFEQLSVPTFKVIKQGLTANSLLDRGMQLSGSTSNTPYTPLE 1587
Mouse        SLHRPLDLDTPTSEESSSSFEQLCVPTFKVIKQGLTANSLLDRGMQLSGSTSNTPYTPLD 1588
Opossum      SLHRPLDLDTPTSEESSSSFEHLSVPTFKVIKQGLTANSLLDRGMQLTGTTSNTPYTPLE 1593
Chicken      SLHRPLDLDTPTSEESSSSFEQLSVPTFKIVKQGLTANSLLDRGMQLTGSTSNTPYTPLD 1588
Coelacanth   SLHRPLDLDTPTSEESSSSFDQLSVPTFKVVKQGLTANALLDRGVQLMGSTSTAPYTPLE 1456
Zebrafish    SLHRPLDLDTPTSEESSTCFDQLSIPTFKMVKAGLSASSLLDRGVQLMGDINSTPYTPLD 1534
             ****************:.*::*.:****::* **:*.:*****:** *  ..:*****:
                                                V1867M
Human        VHGQLRGLDT-TDIGTCAITAIPFEKSKVLFTLEELDEFTFVDETDQQ--------AVPD 1877
Pig          VHGQLRGLDT-TDIGTCAITAIPFEKSKVLFTLEELDEFAFVDETDQQ--------AVPD 1875
Mouse        VHGQLRGLDT-TDIGTCAITAIPFEKSKVLFTLEELDEFTFVDETDQQ--------AIPD 1877
Opossum      VHGQLRGLDT-TDNGTCAITAIPFEKSKVLFTLEELDEFTFVDETDHQ--------AIPD 1881
Chicken      VHGQLRGLDT-TDIGTCAITAIPFGKSKVLFTLEELDEFTFVDETDQQ--------AVPD 1878
Coelacanth   IHGQLRGLDAAEDIGTCAITAIPFEKSKVLFTLEELDEFNFVDETEQQ--------NPSD 1745
Zebrafish    IHSQLRGLDS-SDIGACAITAIPFEKSKVLFSLEEIDDFVLVDETEPSISTEHMPEHNPD 1824
             :*.******:  * *:******** ******:***:*:*: :****: .          *
                                 R1958Q
Human        DSPTGSGYNTDVSDDNLPCDRTSPSSDLNGNSVSDEQDEGVESDDLKKDLPLMPPPPDSC 1997
Pig          DSPTGSGYNTDVSDDNLPCDRTSPSSDLNGNSVSDEQDEGVESDDLKKDLPLMPPPPDSC 1995
Mouse        DSPTGSGYNTDVSDDNLPCDRTSPSSDINGNSVSDEQDEGVESDDLKKDLPLMPPPPDSC 1997
Opossum      DSPTGSGYNTDVSDDNLPCDRISPSSDINGNSISDEQDEGVESDDLKKDLPLMPPPPDSC 2001
Chicken      DSPTGSGYNTDVSEDNLPCDRISPSSDINGNSVSDEQDEGVESDDLKKDIPLMPPPPDSC 1998
Coelacanth   GSPTGSGYNTDVSDDNLPHDGVSPSSDNNGNSESDEQDEGVESDDLKKELPLMPPPPDSS 1865
Zebrafish    GSQTGSGYSTDVSDDNLPNDAQSPASEPNNNSDSDEQDEGVESDDLKKDLPLLPPPPDSS 1944
             .* *****.****:**** *  **:*: *.** **************::**:******.
                                                               P3050H
Human        KAEYKMGRMRSHGMTGAQTRFTFELPNHRLRFTSKVSATDMSTIPPSASLNLPPVTMSGK 3057
Pig          KAEYKMGRMRSHGMTGAQTRFTFELPNHRLRFTSKVSATDMSTIPPSASLNLPPVTMSGK 3055
Mouse        KAEYKMGRMRSHGMTGAQTRFTFELPNHRLRFTSKVSATDMSTIPPSASLNLPPVTMSGK 3057
Opossum      KAEYKMGRMRSHGMTGAQTRFTFELPNHRLRFTSKVSATDMSTIPPSASLNLPPVTMSGK 3062
Chicken      KAEYKMGRMRSHGMTGAQTRFNFELPSHRLRFTSKVSATDMSTIPPSASLNLPPVTMSGK 3056
Coelacanth   KAEYKMGRMKSHGMTGAQTRFTFELPNHRLRFTSKVSPVDMSTIPPSASLNLPPVTMSGE 2928
Zebrafish    KAEYKMGRMKSHGMTGAQTRFTFELPNHKLCFQSKVSPVDVSAMPPTASLTLPPVTMSGQ 2991
             *********:**********.****.*:* * ****  .*:*::**:***.********:
                                      G3385R
Human        GDLDTGSALVLTIESTLITACSSESLVSKGHFKNFCIRFADGFETSWDDWKPEIHGDLVM 3415
Pig          GDLDTGSALVLTIESTLITACSSESLVSKGHFKNFCIRFADGFETSWDDWKPEIRGDLVM 3413
Mouse        GDLDTGSALVLTIESTLITACSSESLVSKGHFKNFCIRFADGFETSWDDWKPEIRGDLVM 3415
Opossum      GDLDTGSALVLTIESTLITACSSESLVSKGHFKNFCIRFADGFETTWDDWKPEIRGDLVM 3420
Chicken      ADLDTGSALVLTIESTLITACSSESLVSKGHFKNFCIRFADGFETSWDDWKPEIRGDLVM 3414
Coelacanth   VDFDAGSALVLTIESTLITACSSESLVSKGHFKNFCIRFADGFETTWDDWKPEVRTDLVM 3288
Zebrafish    IDFDTGSALVLTIESTLITACSSESLVSKGHFKNFCIRFAEGFETTWDDWKPEIRGDLVM 3344
              *:*:*************************************:****:*******:: ****
```

Figure S5: The missense variants identified in the probands affect conserved *KIAA1109* residues.

Multialignments of the regions of KIAA1109 harboring the missense variants identified in the Singaporean (R968C), the Lithuanian (Y1329C and V1867M), the British (M1573I and R1958Q), the Algerian (P3050H) and the Tunisian (G3385R) affected individuals. The position of the putatively changed residue is boxed. The identified codon modifications are indicated above.

Figure S6

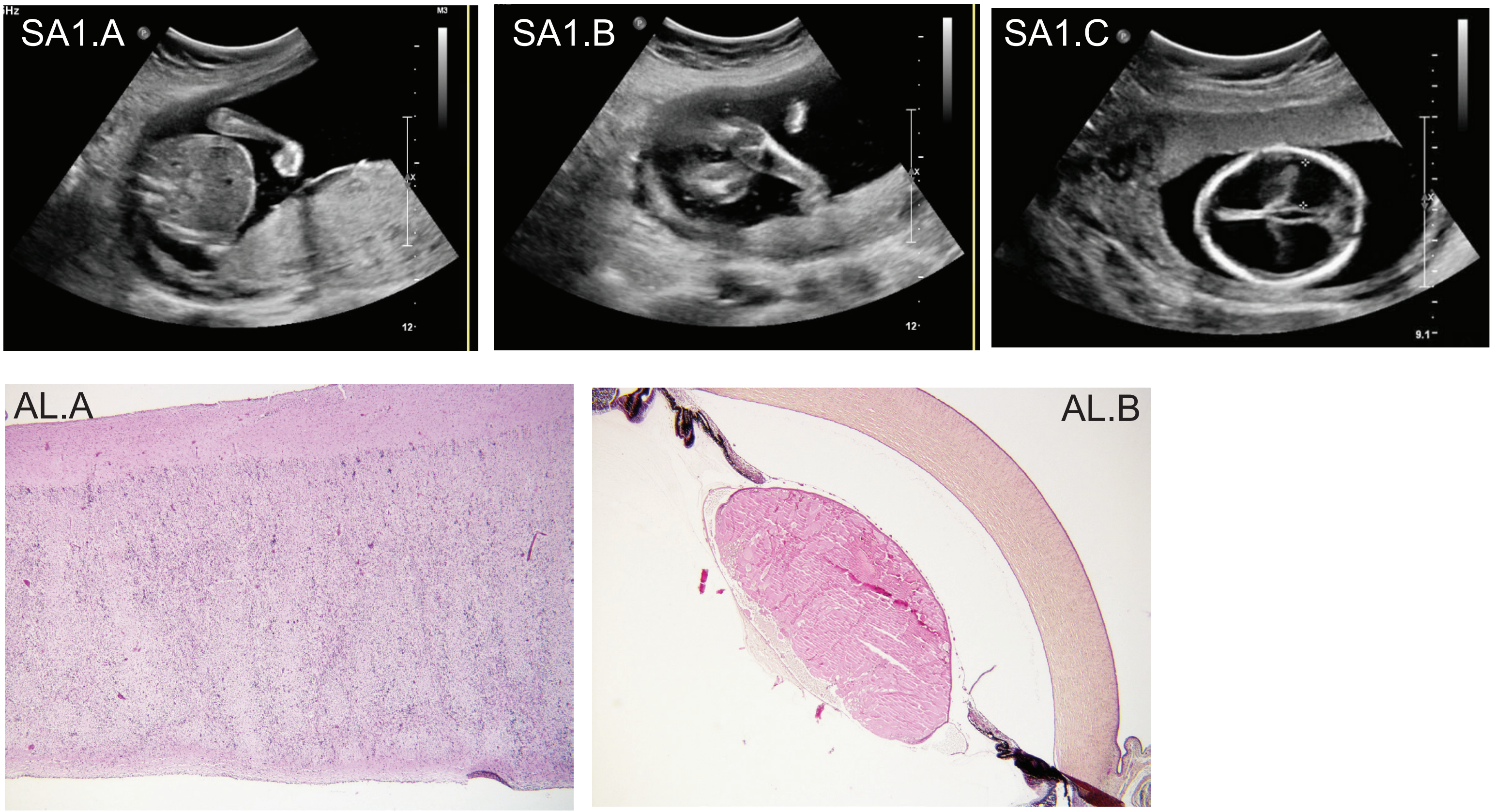

Figure S6. Ultrasound images of the SA1.II.1 and US.II.3 stillborn fetuses and histology pictures of AL.II.1.

SA1(A-C) Antenatal ultrasound scans from the SA1.II.1 fetus showing a fixed flexed hand **(A)**, a club feet **(B)** and cerebellar hypoplasia **(C)**.

AL(A-B) Histology pictures from the AL.II.1 fetus showing lamination defect **(A)** and cataract **(B).**

Figure S7

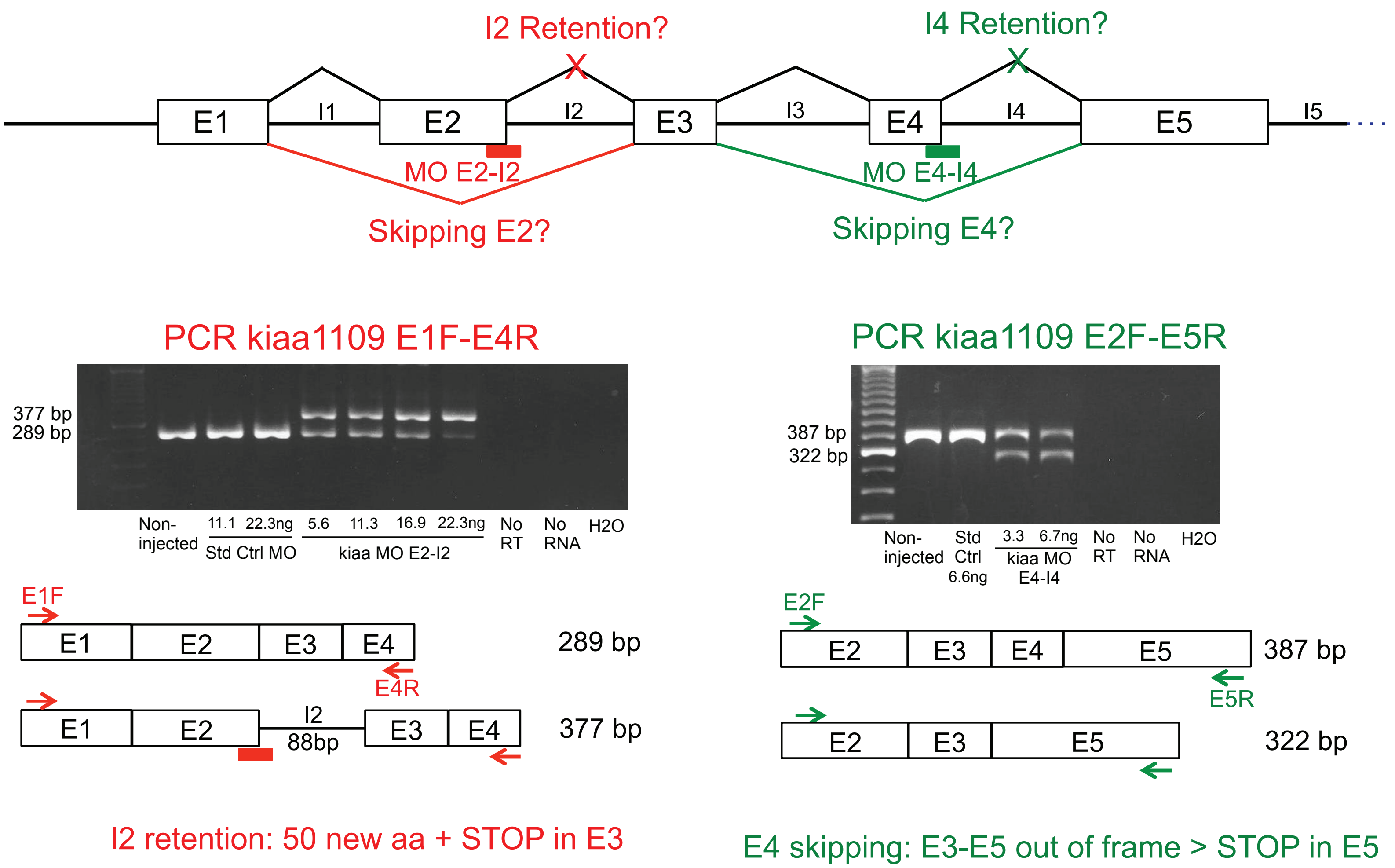

I2 Retention?
I4 Retention?
E1
I1
E2
I2
E3
I3
E4
I4
E5
I5
MO E2-I2
MO E4-I4
Skipping E2?
Skipping E4?
PCR kiaa1109 E1F-E4R
377 bp
289 bp
Non-injected
11.1 22.3ng
Std Ctrl MO
5.6 11.3 16.9 22.3ng
kiaa MO E2-I2
No RT
No RNA
H2O
PCR kiaa1109 E2F-E5R
387 bp
322 bp
Non-injected
Std Ctrl 6.6ng
3.3 6.7ng
kiaa MO E4-I4
No RT
No RNA
H2O
E1F
E1 E2 E3 E4
289 bp
E4R
E1 E2 I2 88bp E3 E4
377 bp
E2F
E2 E3 E4 E5
387 bp
E5R
E2 E3 E5
322 bp
I2 retention: 50 new aa + STOP in E3
E4 skipping: E3-E5 out of frame > STOP in E5

Figure S7: *kiaa1109* morpholino (MO) knockdown strategy in zebrafish.

The top panel shows the schematic representation of the exon-intron structure of the 5' portion of the zebrafish *kiaa1109*. The sites targeted by the two MOs (morpholinos) are indicated in red (sbMO E2-I2) and green (sbMO-E4-I4) and can result in either exon skipping or intron retention.

The consequences of injection of sbMO E2-I2 (left) and sbMO-E4-I4 (right) on the *kiaa1109* transcript are assessed by RT-PCR. The outcomes are schematically depicted at the bottom, i.e. retention of intron 2 (left) and skipping of exon 4 (right). The positions of the used primers (red and green arrows) and the sizes of the amplicons are indicated. The sequences of the used primers are as follow: E1F-5'-GAATACCTCAGCAGGCGCAA-3'; E4R-5'-GGTTGTACATCTTCCACCAACG-3'. E2F-5'- ATCTGACATACTACAATTCTCGCAA-3' and E5R-5'-TATCCCTCTGTTCTCGGCCT-3'. Different doses of both MO were tested. They resulted in the same abnormal transcripts with increased efficiency with increased doses. The agarose gel pictures presented in the middle panels show from left to right RT-PCR performed in uninjected, standard control-MO injected and kiaa1109-MO injected embryos, respectively. The sbMO E2-I2 resulted in intron 2 retention whereas the sbMO E4-I4 resulted in exon 4 skipping. The 377bp band corresponds to the amplimer from the transcript keeping intron 2, whereas the 322bp band corresponds to the amplimer from the transcript skipping exon 4. Both MOs result in early stop codon and are predicted to encode truncated kiaa1109 proteins.

Supplementary Figure S8

A

```
H.sapiens          -----MDQRKNESIVPSITQLEDF---------LTEHNSNVVWLLVATI-LSCGWIIYLT 45
D.melanogaster     MEALEVEDTSDDSLPMGFPEVGTWNSTHNVSLNDMNLDARMIWLLASLL-TTITWVTYIT 59
C.elegans          --------------------MSDFDIQIKIDDAQLDLKGVSFWVTASVVTLFLAWSTFVV 40
                                       :  :            : ..  .*: .: :     *  ::.

H.sapiens          YYNSRNVGLILTLVLNRLY---K-HGYIHIGSFSFSV-LSGKVMVREIYYITEDMSIRIQ 100
D.melanogaster     FYNSRVIGMLITKIANRWF---IKGAYFKIGSVALNP-LAGKIMFRDFVYITYDYTVRAQ 115
C.elegans          LFFSRVSALFFTFVIDKYLRLSKNGIHFKIGGISISGLHAGKIMFRNVIYDNGDMTIKVN 100
                    : **  .:::* : ::          :::**..::.   :**:*.*:. * . * ::: :

H.sapiens          DGFIIFRWWKMYNPKQKQHD-PKAETRLYITVNDFEFHVYNRSDLYGRLQELFGLEPTII 159
D.melanogaster     DGYFIFRWWRSYVPKDVSEDLSHSDTRLSVQLNGYELHIYNRSDLYDTLEKTFGLEPSLL 175
C.elegans          DGHLLFKYWKSVEHRHLNLS-TKRASRLHLVLNGLHVNIYNNLTKYTEIARIRRFDWFFE 159
                   **.::*::*:     :. .  .  :  :** : :*. ..::**.    *  : .   ::  :

H.sapiens          PPKKDDDKTRE---------IGRTRTQSKIERVKVKTESQDPTSSWRS---LIPVIKVNV 207
D.melanogaster     IPTDAASNEERNKLKEHHMNLENARQSQRIQ--NVKNSEAMQATTWRD---LIPVIKIDV 230
C.elegans          N-----------------TNMNDARRPQ-----TKPPDTSPPSSVWENMWNLLGIVHIEV 197
                                       :  :*  .      .   .    :: *..   *: :::::*

H.sapiens          STGRLAFGNHYQPQTLCINFDDAFLTYTTKPPSSHLDQFMHIVKGKLENVRVMLVPSPRY 267
D.melanogaster     CSGRFVFGNRLTPTTLSISVEEAHCTYSTKPAVCRLDHFMHFVKAKVENAKVLFCPSPKY 290
C.elegans          SAGCILVGNKFLPYALWTRFENLNSKTSV--TESANDRALLTFEGETENVAVSLIKNEQF 255
                   .:* : .**:  * :*   .::   . :.    .  *: :  .:.: **. * :  . ::

H.sapiens          VG-LQNDEPPRLMGE-GFVVMQSNDVDIYYYMDEPGLVPEETEENIEGEMSSEDCKLQDL 325
D.melanogaster     TG-LI-DEPPRYMGE-GFVVMMSNQMDLYFYMDEPGVVPEHPVQIVL----PNGDVVEPS 343
C.elegans          DFTAKDKDPPRTMGNDGCPLLQSASLEFVYKQDLLGYVTDDEPQS-----------ITLK 304
                          .:*** **: *  :: * .::: :  *  * * :.  :            :

H.sapiens          PPCWGLDIVCGKGTDFNYGPWADRQRDCLWKFFFPPDYQVLKVSEIAQPGRPRQILAFEL 385
D.melanogaster     PPVWGINARCLRGTDFSYGPWADRQRDHLYRYFYPSDWKEAEVTPTPQPGELRSYQSFDV 403
C.elegans          LPLWSSEWRFGNNTVLSYGPWAEQQRFLIYSFFYPPDFQNSTATAMPTRGKKRIHVKHDV 364
                    * *. :     ..* :.*****::**  :: :*:* *::    .:      *. *    .::

H.sapiens          RMNIIADATIDLLFTKNRETNAVHVNVGAGSYLEINIPMTVEENGYTPAIKGQLLHVDAT 445
D.melanogaster     TLCVLNEATIDILFSKEKETNAMHITVGPASYVEMTIPWVTQPDGYTSKIQGQLFHVEAT 463
C.elegans          KIILTKETCMDIWFMRGEQLESIRTRCGPLSSLDMSILWITTEKGFYWNMKAEFLNFEAT 424
                    : :  :: :*: * : .: ::::    *  * :::.*    .  .*:   ::.:::::.:**

H.sapiens          TSMQYRTLLEAEMLAFHINASYPRIWNMPQTWQCELEVYKATYHFIFAQKNFFTDLIQDW 505
D.melanogaster     TSLQYRSLAEFESLEYKVRIHYPTKWNAPQDWSISLSGCKTSAFIVYKHKCFFQDLIEDW 523
C.elegans          TSLIFTKLFSCKKFNVDGSFVYPLTWNGEQTWTIDYAFTKANAWFVWDHKRLFTDLINDW 484
                   **: : .* . : :  .     **  **  * *  .    *:.  ::: :* :* ***:**

H.sapiens          SSDSPPDIFSFVPYTWNFKI-MFHQFEMIWAANQHNWIDCSTKQQENVYLAACGETLNID 564
D.melanogaster     ANKARPDILSFVPYTCNFSI-RLHEFEILMLCNEYNWIDCSSANQENNHLAFCGDVFEMS 582
C.elegans          IGDDPSDISKFVPFRVHNRMKVVDGFEVIMLLNESNWVDTADMNAENVEVAIVGEKLSFE 544
                    ..    ** .***:  :  :  .. **::    *: **:* :  : **  :*  *: :.:.

H.sapiens          FSLPFTDFVPATCNTKFSLRGEDVD-LHLFLPDCHPSKYSLFMLVKNCHPNKMIHDTGIP 623
D.melanogaster     FALPFDDFLPKTVTLKFWIHGEGLD-LSLYVPEVSSVRPIVLAIDENAR---LLTREG-- 636
C.elegans          CELPFVDFLPQTQMVKYEMRGEKSVAMRAKFPPDSATAPIRAALSRLAR----------- 593
                     *** **:* *   *: ::**    :   .*            : . .:

H.sapiens          AECQSGQKTVKPKWRNVTQEKSGWVECWTVPSVMLTIDYTWHPIYPQKADEQLKQSLSEM 683
D.melanogaster     -KLIRRPELYSKKWRKICQRSAGWIDCWAVPILALSIQYVYHPVPPLGPDPQADITTPEK 695
C.elegans          --CNSY--APPSKHGTHSLDTDVWFELWRTELVKMDFDHHYRPLIVKSNIPS-D------ 642
```

```
                          *  .    .  *.: * .  : : ::: ::*:       . .

H.sapiens                 EETMLSVLRPSQKTSDRVVSS-PSTSSRPPIDPSELPPDKLHVEMELSPDSQITLYGPLL 742
D.melanogaster            EEILLSPMRIPKVRKSPVSSWQQPPEQYSKFDPGTLAADHVTVELEIGS-SVLMAYGNVL 754
C.elegans                 ------------IPFSILSDYLPPP----ANHPWDLEPDYLGVDILIEG-SDVKFTGLLV 685
                                         . : .           .*  *  * : *:: :   * :   * ::

H.sapiens                 NAFLCIKENYFGEDDMYMDFEEVISSP---VLSLSTSSSSGWTA------VGMENDKKEN 793
D.melanogaster            RNFISLKENIFGEDQNFTDMEQSNVNMKEPGVAQVNPK-DQLLAKEKELANKSISETQPP 813
C.elegans                 KLLFELKNNYFGWYDSMTSVDDEKIDD--PI-KLK-----------------ASFD-KTN 724
                          . :: :*:* **  :   ..::   .                             : :

H.sapiens                 EGSAKSIHPLALRPWDITVLVNLYKVHGRLPV-----HGTTDGPECPTAFLERLCFEMKK 848
D.melanogaster            EEKRKPFDPRLYRPLEVMVSVIVHDIQAHVMK-----NCNEDDPPCPVVLIERFGFEMNK 868
C.elegans                 ANGMKPV--EYFRTMNVDVTVRVCNVRAEMLLYSPAIDEGAEPEKVPVVFVEEVAVEVKK 782
                              * .     *  :: * * : .::..:       .   :    *..::*.. .*::*

H.sapiens                 GFRETMLQLILSPLNVFVSDNYQQRPPVDEVLREGHINLSGLQLRAHAMFSAEGLPLGSD 908
D.melanogaster            KYHETTLQVLVSPSYLLTSDCL-QRSQREQHINQGHLMLSAVQVRGHAMFSNEGCALDED 927
C.elegans                 TKTQALIQVGVSPACAYLDKSS-------QGSGPGCITLSGFQFRGHAMYSAKEVAWNMG 835
                             :: :*: :**     ..         :    * : **..*.*.***:* :    . .

H.sapiens                 SLEYAWLIDVQAGSLTAKVTAPQLACLLEW-GQTFVFHVVCREYELERPKSVIICQHGID 967
D.melanogaster            TLEYSWLVEVQLGKLTGKLTLPQLVNVVTG-LETLILLAIDPENCLKSPKTVRNCHHGVP 986
C.elegans                 LVEYGWIMEILVGDIAGTLDFPAHAHVLHQIMESLLMFVISPDDATKVPDRMQFCQHGQL 895
                           :**.*::::  *.::..:  *  . ::    ::::: .:  :   : *. :  *:**

H.sapiens                 RRFCE---SKLSCIPGPCPTSDDLKYTMIRLAVDGADIYIVEHGCATNIKMGAIRVANCN 1024
D.melanogaster            SNLCP---QTKEEKKYKCPSSEDIKYKMTRVSVDAVDVYLIESGTALHAWISPIRLANCN 1043
C.elegans                 IKACSIAGKKTNEILGPCKTEEQMKYRQIRISVDSVNLTFVEEKTILQISADPVRVTICN 955
                           . *    .. .     * :.:::**   *::**..:: ::*     :   . :*:: **

H.sapiens                 LHNQSVGEGISAAIQDFQVRQYIEQLNNCRI----------------------------- 1055
D.melanogaster            LHGQRVKSGISGLLPSILLRLFMLHTTNSTFNTNTTGSNRSGKLRRADQDSLKSQDAGGS 1103
C.elegans                 AHESRFTEHVCIRVPGISIRQAVRIK---------------------------------- 981
                           * . . . :.  : .: :*  :

H.sapiens                 --------------------------------------GLQPAVLRRAYWLEAGSANLGL 1077
D.melanogaster            HYASHGKTGKRSSNSFSRRDSREEATRKLRGSFSETHTKRTPETEITENWVEVGCTSLGP 1163
C.elegans                 -------------------------------------------EKPENIWIEGANAAIEG 998
                                                                           *:* . : :

H.sapiens                 ITVDI--ALAADHH---SKHEAQRHFLETHDARTKRLWFLWPDDI--LKNKRCRNKCGCL 1130
D.melanogaster            ILLEGASALPIPDH---ELHLVQHNFLREHDAKFKRLWFLWSNNGSALSSGSEISRCGCI 1220
C.elegans                 VSLDI--ELPTPKSASPTIGKERLEFVRMHDADTKRLHFLWADHS--------VWGCACF 1048
                          : ::    *   .         : .*:. ***  *** *** :.            *.*:

H.sapiens                 GGCRFFGGTVTGL-DFFKLEELTPSSSS---AFSSTSAESDMYYGQSLLQPGEWIITKEI 1186
D.melanogaster            GGCAFFGSNRNGQ-KFFKPTAQDAHDNYNIARYFIINNNKDFGFGESILHQGQLVFHTPP 1279
C.elegans                 GNTCFFGDVDEIGSTFMETLT--------KKKFFVPGIERNPEKQPQVM-QSVILKNKPI 1099
                          *.  ***.       *::              :   . : :     .::  .  :  .

H.sapiens                 PKII------DGNVNGMKRKEWENKSVGIEVERKTQHLSLQV--PLRSHSSSSS----SE 1234
D.melanogaster            YSLHCVSLYDTADFNGKGRLYRPAGDLRNGSLKKTDLCSLPDGTKFKIGASGTTGVEKPE 1339
C.elegans                 LS---------------------------------------------------------- 1101
                           .

H.sapiens                 EN----SSSSAAQP--LLAGEKESPSS------VADDHLV-----QKEFLHGTKR-D--- 1273
D.melanogaster            PNCRNKSRESIGSPNTLERRTKRYPCTRQTSVDVPYARLLDSPSKKLQLQHEASAGDAGS 1399
C.elegans                 -----------NQPHMFYKKPKNA--------DVV----I--------TIRKESTGDT-- 1128
                                      .*  :    *.          *     :          :  .  *

H.sapiens                 ------------------------------DGQASIPTEISGNSPVSPNTQDKSVGQSPL 1303
```

```
D.melanogaster        SHRRGSDSNRLRVSPPKTSISDSRLTGDVLDDETEIPDEMSHSAPH-------------- 1445
C.elegans             ------------------------------------------------------------ 1128

H.sapiens             RSPLKRQASVCSTRLGSTKSLTAAFYGDKQPVTVGVQFSSDVSRSDENVLDSPKQRRSFG 1363
D.melanogaster        SHPLEFEIA-------DIALHVQGLGGDQLPREVQRTI---------------------- 1476
C.elegans             ------------------------------------------------------------ 1128

H.sapiens             SFPYTPSADSNSFHQYRSMDSSMSMADSEAYFSAAEEFEPISSDEGPG-----------T 1412
D.melanogaster        --------------------SLTSENPSEMFFSAEEDISNVLSQRGSMKQRNSVNSGSVV 1516
C.elegans             ----------------------------ESFHSARS-------QQSPGL----------- 1142
                                                  * :.** .       :..

H.sapiens             YPGRKKKKK-----------QTQQIDYSRGSIYHSVEGPLTG-----HGESIQDSRTLP- 1455
D.melanogaster        LSGKKRFSSDLSIGAQNDNGSHTLPTYRSDLEIHAPDGNKTLPKRPQSTTELADSRGSSS 1576
C.elegans             ----RI---------------------------------------------------LQS 1147
                          :

H.sapiens             FKTHPSQASFVSALGGEDDVIEHLYIVEGEKTVESEQITPQQPVMNCYQTYLTQFQVINW 1515
D.melanogaster        GTPSLSSNSFISAMSSQEDVALV----NLHQQV-NRPIIDSPLLMASYLNHLSQVKCFNW 1631
C.elegans             MEMSSSYATFVDNVRV-----------ELPSAITV----P---------QFGEPGAILEW 1183
                           *  :*:. :             :  . :                 .      ::*

H.sapiens             SVKHPTNKRTSKSSLHRPLDLDTPTSEESSSSFEQLSVPTFKVIKQG--------LTANS 1567
D.melanogaster        NGCSFPLGP---DVFSTPLFSENE--DGGLTYIGSKMLPHFDLYSCWREIKVVPRYENAT 1686
C.elegans             CQAHQATRI----------INDV----------------------------------NTS 1199
                                           :                                     :

H.sapiens             LLDRGMQLSGSTSNTPYTPLEKKLA---DNTDDETLTEE-WTLDQPVSQTRTTAIVEVKG 1623
D.melanogaster        GSNSSATFMGGPKSHPWDPSVLLKEEESDKTTNGFDDGEFMSLQAEGGAVCTSVVARLKG 1746
C.elegans             GVNE-VRFLSKPKKS--------------------QDIEYNTSRDTLGKRRLAINGVAAT 1238
                        :    : .  ..                        *  :     .    :

H.sapiens             TVDIVLTPLVAEALDRYIEAMVHCASTRHPAAIVDDLHAKVLREAVQNSKTTFSENLSSK 1683
D.melanogaster        QLNVFLTPLLLEGLQRMVEAAVPTIQSMHLLSVVNFIHTSCIAKVNNDNILKRDQSLSYW 1806
C.elegans             SLDLFVTPIGIEAFERLVTAASHSVPAINPCILVHMCYRDCVLKKHRQPLT---ESL--- 1292
                       :::.:**:  *.::* : *      : :   :*.  : . : :  .:      :.*

H.sapiens             QDIRGTKTEQSTIGTTNQGQAQTNLTMKQDNVTIKGLQTNVSIPKVNLCLLQASVEESPT 1743
D.melanogaster        SQVHS-NSKRSTTERHLQGPGDSFLSDVYEESISTKTQGLIVLPKVSITMLQSSIVEEII 1865
C.elegans             -----------------------FADEDNDSEPISEVDITVDLPRVSIGLFQCGVVKKNIV 1329
                                                   :.   .  :  : :*:*.: ::*..: :.

H.sapiens             TA----PSRSVTHVSLVALCFDRIATQVRMNRGVVEET------------SNNAEPGRTS 1787
D.melanogaster        SVAALDNVQDLSCVSLLTFYMEGISTKFHMGKTTRASMHNVYIQQTVQSGSSNKKGGIMK 1925
C.elegans             KSNHTDHITANMGLL----LIDRA-----------------FIQSKLIPAE-----SVSQ 1363
                      .            :      ::                            .     .  .

H.sapiens             NFDRYV----HATKMQPQSSGSLRSNAGAEKGKEIAAKLNIHRVHGQLRGLDTTDI---- 1839
D.melanogaster        GTRALLAHLSSQTRPDNVQGEPILIETSEKQLEEVVITLDIGRAHAQLRRLKTEGQSCTQ 1985
C.elegans             DFSADTSN-----------------------LSSTLYQLNGSAITVQLLQLTNRDA---- 1396
                      .                               ..    *:      **  * . .

H.sapiens             -GTCAITAIPFEKSKVLFTLEELDEFTFVDETDQQAVPDVTRIGPSQ--EKWGW------ 1890
D.melanogaster        DSPIIVTAIPEHKSKVLFECLKMPE------------------STGI--ESIGY------ 2019
C.elegans             -------------------------------------PDFGSSGTATTPNNWEHCAISRR 1419
                                                                 . .   :.

H.sapiens             -------IMFECGLENLTIKGGRQSGAVLY--------NSFGIMGKASDTERGGVLTSNN 1935
D.melanogaster        -------IMFECGLEGVGVKIVKRSHFEKS-ENSKEELAEMAGAGAGGGASAGGFNLNDL 2071
C.elegans             MNNLEPRVMMDFNVSDTLIIILERRPIILLLPDKSTTAITPIH--SPA------------- 1464
                             :*:: .:..  :   ::                :   . .
```

```
H.sapiens           --SSDSPTGSGYNTDVS--------------DDNLPCDRTSPSSDLN----------GNS 1969
D.melanogaster      VGQGEGAAGSGDGGATSKAEAAWRLITKKPPTPKTPKEKFQPASDSNISAETSGAEKGKS 2131
C.elegans           ----NAPTPT----A----------------MNRTPTLT--------------------- 1479
                        :. : :                       . *

H.sapiens           VSDEQDEG-----------------------VESDDLKKDLPLMPPPPDSCSMKLTIKE- 2005
D.melanogaster      TPKPPDEDVEKGTSPANAQTGAQKPSAGAGTNTKDNYDKVLSNVKETDKTSSCVIELKA- 2190
C.elegans           ----------------------LTPSAGAGGGERAEP-----MRKKKK--MICEHYLKAD 1510
                                                       :                    :*

H.sapiens           -----IWFSFAAPTNVRSHT--HAFSRQLNLLSTATPAVGAWLVPIDQLKSSLNKLETEG 2058
D.melanogaster      -----VWFNFAAPPCVPITRKIDLTRLDWNLLSTASPAITAWMNPSNRLAMKIVSLMKAL 2245
C.elegans           IGSVTTALVMARPQEL-------TAGDEFPIYEALAPVMVSWLSVVENFLRTVDKFIHTV 1563
                           : :* *  :           :  : .: :*.: :*:   :.:  .: .:

H.sapiens           TLRICAVMGCIMTEALENKSVHFPLRSK----YNRLTKVARFLQENPSCLLCNILHHYLH 2114
D.melanogaster      HTRQTAVAACLMAEAMDNEKIQRNPKIKKSRYANNYTLLSKTLQEDPSCQLCYIMQKLVL 2305
C.elegans           ECWKSVAMAKVLKLALDSTDEKVVVKVGKNRM-GRTRVLSA---HQASCPSCILLKTLFR 1619
                         .. . ::  *::. . :   :       ..    ::    .: **  * :::   .

H.sapiens           QANYSIIDDAT------MSDGLPALVTLKKGLVALARQWMKFIVVTPA---FKGVSLHRP 2165
D.melanogaster      DEGVQRIETIF------KQHDVPHLNTLRQGIIVLSRQWKNTLYNPIL---FEHQYKNKL 2356
C.elegans           WFAYAGNAPGAINHRLDIRPEFEIEETRKTALMALLSHWQSDVGKELKLVSYEDAHRFKV 1679
                                         .    * : .::.*  :* . :        ::     :

H.sapiens           AQPLKPQIAMDHEHEDGLGLDNGGGLQSDTSADGAEFEFDAATVSEHTMLLEGTANRPP- 2224
D.melanogaster      SRPINVTFSFPQNEEDAENDECEGDVEMGA--------------------FAGVGENPEE 2396
C.elegans           TRPDEAAIVALTK---------SKRLKR------KMLEKKESSKKETRVVMEVKPEQPK- 1723
                    ::* :  :    :            ::                       :    :.*

H.sapiens           ---PGSSGPVTGAEIMRKLSKTHTHSDSALKIKGIHPYHSLSYTSGDTATDSPVHVGRAG 2281
D.melanogaster      SATYGHEGANHGTASQRS-----------------------------TSTSPNIHHPRRG 2427
C.elegans           ----AKRGRMSPAPLLKKLRKK----------------------AGDDDFD--------- 1748
                        .  *    :   :.                                .

H.sapiens           MPVKDSPRKESLLSYLTG-SFPSLHNLLEGTPQRSSAAVKSSSLTRTGNTVATDMLS-EH 2339
D.melanogaster      ---------IQMLPIISG-QVPEFEY----------GALQEGSL------SSTNSVN-KS 2460
C.elegans           ---------DDSMKFLSDVEMQEFNTLP-----------------LYEDYEDDEMLENLD 1782
                              . :  ::. .. .:.                            : :.

H.sapiens           PLLSEPSSVSFYNWMSNAVGNRGSVLQESPVTKSGHNSLPTGVAPNLPTIPSASDFNTVL 2399
D.melanogaster      WLGTENHKEDLYFWMAKQQDNKKKHFTEKEHAR-----------PAPPKM---------- 2499
C.elegans           SEPKIDDKVDLYTWMRNAQRESTL--RRRKLAGGAEGSVKDDL----------------- 1823
                       .   . .:* ** :   :      .   :

H.sapiens           SSDQNTLDGTHSQHSTSQDDVAGVEEANQGFPAVQLADAQVVFKPLLSHTGIQSQDT--- 2456
D.melanogaster      ------------------TEHAGQTRSGIMQDSIKLLDAHLIFEPLLTCLGVMPQQM--- 2538
C.elegans           ------------------------------------------NLKGYINPMDIQQKAYYYN 1842
                                                              ::  :.  .:  :

H.sapiens           MP------FCYR---MYFGEHLSFSGTLDCLRADIVDSDTAKERKGKRARRQGHVNLP-- 2505
D.melanogaster      INKFSNADISSL---ENFGTNLSLIGTFDSIRVDIVVSEAGDKKNSAQK--PAKLNKKSN 2593
C.elegans           IYRWAQLQWTSLDGIEKDHWHLDYSVTLREVDVRMMAKSI---KNSSD-----HLRQYIT 1894
                    :                   :*.   *:  : . :: ..    ::.       ::.

H.sapiens           ----PLEFK-PALMLGTFSISAVV--MEKSVCTPQNSTSALSFHDLSKRYYNTFHCNFTI 2558
D.melanogaster      GGRASIMMDTPLFLCERVGVELEVLKMSDGMVD-QARQNVIYMSRRQLKKHTSTVINFSL 2652
C.elegans           PAQQKVMQVRNAAVN-----GGMVWKMER-------------DERRKIPLHGQWNISYSG 1936
                         :       :         *  *.                  .   :     .::

H.sapiens           SCQSISQHVDMALVRLIHQFSTMIDDIKATQTDIKLSRYTAGS----------ASPT--P 2606
D.melanogaster      NVRFISQQVNMPLLRLLHQICNMYQNVKDAQNEFHDQPELSKKSQTKDECSLASEPTDIV 2712
```

```
C.elegans           NVEGIRFLIGMATVSLGKELSLVLRVAMEAKNELRMHSTAESFQ---------TPRNEVK 1987
                    . . *   :.*  : * :::. :      ::.:::                  :  .

H.sapiens           TFKTR-KHR-----DFR---S---SDF--------SRSSRGSLNGGNRVN---NAKNKRT 2643
D.melanogaster      PFNSM-SERYNHAENYSDERY---DKFNETMPTMLARPRPGGLAPIIQLTPSPNAKNRPQ 2768
C.elegans           VFKPVVPNQYDLAVEWDEKVLDMTRDYEKHM----------------------------- 2018
                     *:    .:     ::         .:

H.sapiens           NNENNKKESR---NKNSLGRSERRT---SKVSRKGSKDVVDHMTIHMDD----------- 2686
D.melanogaster      SF-AQKLRSTGKSVKGKLGYTNLNESSSSPLRDSPTMSLHEHNILKMSTESKASLNGACA 2827
C.elegans           ----QRMRT------------------------NKEDVVEKVKV--------MVNGSAM 2041
                        :: .:                          . .: ::  :

H.sapiens           ------------SDSITVSEQSEPSAECWQNMYKLLN--------FYSLISDPTGILEKS 2726
D.melanogaster      TSVSGDYQNTLTKAGMPTMAPMLETPNCWKTIYHLLE--------LYGTMPETKTVVQRS 2879
C.elegans           V-----------------S--SIVLESVLNDLYVSVTISQIVLAHSKNPMPDIPVVVHAV 2082
                                              .  : :*  :          . : :   ::.

H.sapiens           SETFGPAGVRSPTEPTCKVVFENEQDN--S---SLTKTQRKRSLVTSEPQHVTLIVFGIG 2781
D.melanogaster      SLNEHKSK---------SAGFAHDDDDLASAPTPLPQHREMLLVDASSQERTRLIVFGVA 2930
C.elegans           TLSTTPTA---------AA---AEDKKTA----TLTKK----SV---------SSTFKID 2113
                    : .   :           .    ::..       * :      :            .* :

H.sapiens           MVNRTHLEADIGGLTMESELKRIHGSFTLKEKMKDVLHQKMTETCATAHIGGVNIVLLEG 2841
D.melanogaster      KIHKTRLLATLSGLKLESEITTLNSTATWRKKARPVS----LECSLTGQVGRAMIVLLEG 2986
C.elegans           D-----LTVSLTKMKL--T---------LSEADSSNKKSDILRCTLNSSSFNVHT----- 2152
                          * . :  :.:              :           .   ..    .

H.sapiens           ITPNIQLEDFPTSPTSTAKQEFLTVVKCSIAKSQALYSAQR-GLKTNNAAVFKVGAISIN 2900
D.melanogaster      VAPSQQ-----------------TVVKVTVGKSQTLYSSLSKRGKDKNSGLLSIGAVNID 3029
C.elegans           -----NLKT-------------LTSAKESNRPKNNL-----INSNIATTATLRLGALEGT 2189
                         :                 * .* :   .: *        :  .:. : :**:.

H.sapiens           IPQHPATLHSMMVRSSHQLSKQISDLIRQPSTAPQPVKED----IATPLPS--------- 2947
D.melanogaster      IPQHPVALHGMMTRSSKQLSSTLQELRVKRNSGRSTMRSHTAEEPESPFHARNSVGASSG 3089
C.elegans           MPMAAYSLHDVVMRHGKELEQQLNRLAAQPASTPLSS--------STPFPSAEQSLLAKV 2241
                    :*    :**.:: * .::*.. :. *  :  :               :*: :

H.sapiens           -----EKTPTS------VNQTPVE----TNEFPQL------PEGLEKKPIVLKFSAMLDG 2986
D.melanogaster      TGVASEMRERTVSGGAGAQQQPQQAARRAAHMAQSKTGTAQHQNGLLQPLVMQFNVLLQS 3149
C.elegans           ADMKTAPEPVVIT---QAEFKPLTTLPATAAHVQDAKG---QIVRRVPVAVVSFSIELTS 2295
                                     .:  *      :    *               *:.*.  * .

H.sapiens           IAIGAALLPSLKAEYKMGRMRSHGMTGAQTRFTFELPNHRLRFTSKVSATDMSTIPPSAS 3046
D.melanogaster      LSINAALLPSLQAQYRMNHVSSMGVTGQRAKFVIDLPTHTLSFNTKIQN--EMNLPSEAC 3207
C.elegans           IEMNIQLLPSLQAKYRINRATSNGITGVQANWSILLDEHFFEFCVTGQGGKT----ETFR 2351
                    : :.  *****:*:*::.:  * *:** ::.: : *  * : *  . .

H.sapiens           LNLPPVTMSGKYIMEEHDSYSDQVWSIDELPSKQGYYLQGNYLRCVAEVGSFEHNLTTDL 3106
D.melanogaster      IGLPPVHVLAEYIPDHRQDHTENVEG--------IVLRQGGYVNASAEIGEFERCLTTDL 3259
C.elegans           LQLPSVTSDGLYQAEQGV------SSQKPSTDKKLIYREGGSLQMTVVLGRVNHIFTTEL 2405
                    : ** *   . *  :.         .          :*. :.  . :* .:: :**:*

H.sapiens           LNHLVFVQKVFMKEVNEVIQKVSGGEQPIPLWNEHDGTADGD------------------ 3148
D.melanogaster      LNHLVFVQKVFMREINEVLQKVYGGEKPVPLWTEESGDSSGA------------------ 3301
C.elegans           LNQLMFAEHSFRTELTALINRIRSSSFASTNSSRSAQSTNDRVNSTANLKLLLPVQTTST 2465
                    **:*:*.:: *  *:. ::::: ...        ..    :..

H.sapiens           ----KPKILLYSLNLQF----------------------------------------KG 3163
D.melanogaster      VQESSLKRILFSINISM----------------------------------------KR 3320
C.elegans           PVHIEKPPLLFSIKIQTKEIPRKDEKTDKDAKTPKASGASGNLDQSQHPSHSTHVKSTPW 2525
                        .   :*:*:::.
```

```
H.sapiens           IQVTATTPSMRAVRFETGLIELELSNRLQTKASPGSSSYLKLFGKCQVDLNLALGQIVKH 3223
D.melanogaster      IQLTATTPCSSAVRFETGILELQLSNRVKNL---GDMSNRKLFFKAHIDFNLSLGQIIRN 3377
C.elegans           LQLTAATPTQTAVRLTVDSLEGELTNKWVVK---EEGSKERIYGNAVIHFNAKLGQLIKP 2582
                    :*:**:**   ***: .. :* :*:*:         . *  ::: :. :.:*  ***:::

H.sapiens           QVYEE-----AGSDFHQVAYFKTRIGLRNALREEISGSSDREAVLITLNRPIVYAQPVAF 3278
D.melanogaster      VIFDE-----AEPEFQQYAFFHTTINLRNAFQDELLN-EDKELILLTLKRPLVYVQPIAV 3431
C.elegans           VPTGDSVAATDVTDLQEFATFMTQVRVENKERNMFNS---SYSYHISLNRPIFLVKAAAI 2639
                        :         :::: * * * : :.*  :: : .         ::*:**:. .:  *.

H.sapiens           DRAVLFWLNYKAAYDNWNEQRMALHKDIHM----------------ATKEVVDMLPGIQQ 3322
D.melanogaster      DKAILVWLNYKNAYEYWAEKRANLCHEHAQHSLLSQYSGQHQNQNMQNPQVFDR-VAFGQ 3490
C.elegans           DKAILLWLNYKNTYDYWRNEREKVVQEKTTTKLSN--------AGMFSP---------TQ 2682
                    *:*:*.***** :*: * ::*  : ::                      .           *

H.sapiens           TSAQAFGTLFLQ-LTVNDLGICLPITNTAQ----SNHTGDLDTGSALVLTIESTLITACS 3377
D.melanogaster      IAGSNLSTLFLQ-LTVEDMGICLPLKQVNTTTFGSRSYQDFDAKGAVVITLENTIISACN 3549
C.elegans           IAEDA--DMNLSLAINNGMYMCMPLYSHDV----------TEGMPALVLSLQKSNLSVLV 2730
                     : .    : *.    :.: :*:*: .                :   *:*::::.: ::.

H.sapiens           SESLVSKGHFKNFCIRFADGFETSWDDWK-PEIHGDLVMNACVVPDGTYEVCSRTTGQAA 3436
D.melanogaster      SGALVSKGKFQGLCLRFADDFETNLDDWK-PNSA-EPIMNVCVVSEGTFEVCSRTTAAK- 3606
C.elegans           KKELTCKASFNGFKCSFVDDFDEQALTQSFLDATHSDQSNCIFFPEGTYQLCSKAEATK- 2789
                    .  *..*. *:.:   *.*.*: .     .  :   .   *  .. :**:::**:: .

H.sapiens           AESSSAGTWTLNVLWKMCGIDVHMDPNIGKRLNALGNTLTTLTGEEDIDDIADLNSVNIA 3496
D.melanogaster      --KGENAKWLLNVKWQMEGVDIHLDVNIGKQLSSLGHTLTMLTGFEEDETQMESPDSDEG 3664
C.elegans           ----GPAKWVLSVSAEMQGVEIDLDTRIGKLAKLLVNTFSMIRTDDDDDMSFWG---DEG 2842
                          ..* *.*  :* *:::.:* .***  . * :*:: :   :: :        : .

H.sapiens           DLSDE-DEVDTMSPTIHTEATDYRRQAASASQPGELRGRKIMKRIVDIRELNEQAKVIDD 3555
D.melanogaster      DQSCR--------DTFVRRRGDFDNLPAFVFDPTI---DSKKRSFMMEKEMAEQLKIIND 3713
C.elegans           ELDSDEEKVE--------GASELKKL------------KAEEKVPWMENKMHEHSRAVFE 2882
                    : .                    :  .                :      .:: *: : : :

H.sapiens           LKKLGASEGTINQEIQRYQQLESVAVNDIRRDVRKKLRRSSMRAASLKDKWGLSYKPSYS 3615
D.melanogaster      LRTLGASHNTVAHEERRLQELQAICYKYFRRDMIQKWKRPSLRRSLKT----------YG 3763
C.elegans           LAARGVSNKLIEAEKHKLRQYELIRFKAFRRNVVEKLKKGTTASRQHTE----------- 2931
                    *   *.*.  :  *  :: :: : :  : :**:: :* :: :       .

H.sapiens           RSKSISASGRPPLKRMERASSRVGETEELPEIRVDAASPGPRVTFNIQDTFPEETELDLL 3675
D.melanogaster      RSHSYIGSGSS--------VSGVGVPQTLDNV----SYTGRRL-----DTIASNDEISSL 3806
C.elegans           ---------TP-------------------------PPQPRP-----DT---------- 2941
                                                             *       **

H.sapiens           SVTIEGPSHYSSNSEGSCSVFS---SPKTPGGFSP-GI----PFQTEEGRRDDSLSSTSE 3727
D.melanogaster      Q---STP--------ASCHSRSASLKHTTGGGVIGGGVNALGRVTFTEAMRQTSLPNADT 3855
C.elegans           ---------------------------------------------TSRRNSRTTS--- 2951
                                                                 . *:    .

H.sapiens           DSEKDEKDEDHERERFYIYRKPSHTSRKKATGFAAVHQLFTERWPTTPVNRSLSGTATER 3787
D.melanogaster      DTETADNELD--------WRGDITPSEIDVDGTSV----------EMRRKHGHGQKQPEP 3897
C.elegans           --TSQKNS-----------------------------------------EDLTTPGDIE 2967
                       . .:.                                          ..

H.sapiens           NIDFELDIRVEIDSGKCVLHPTTLLQEHDDISLRRSYDRSSRSLDQDSPS-------KKK 3840
D.melanogaster      NIDFELDIKVLVNSGKCVLHTKDTGEER-------GYAGGSGAATSGVPASSVKSHKREK 3950
C.elegans           TVNFNLDVKVNITSGTCTLRTQKKEGAN-QL---------------ALPGIL-----KRL 3006
                    .::*:**::* : **.*.*:        .                  *.       :.

H.sapiens           KFQTNYASTTHLMT-GKKVPSSLQTKPSDLETTVFYIPGVDVKLHYNSKTLKTESPN--- 3896
D.melanogaster      SIGNDWGSPTPSRRQRDKSKLRYNANALLADLTIFHIPGLDVKLHYQSKTLAEQLTG--- 4007
C.elegans           NLG--------------TKDIKAMFEPQIITTTTFSIPSVEIKAYHVSDPSNRSTDEFCK 3052
```

```
                    .:               .       :      * * **.:::* :: *.    .

H.sapiens           ----------ASRGSSLPRTLSKESKLYGMKD-SATSPPSPPLPSTVQSKTNTLLPPQPP 3945
D.melanogaster      --------------DQL------------------------------------------- 4010
C.elegans           DKREKISKGLAKDADKLHRDLHNKSRFGNTYINGGGGP-----------KTST-V----- 3095
                                  ..*

H.sapiens           PIPAAKGKGSGGVKTAKLYAWVALQSLPEEMVISPCLLDFLEKALETIPITPVERNYTAV 4005
D.melanogaster      --PT---GRRMGSKRATLCAWMTLQSIPEETIISPHILEFLEQTLEPIPARQSSSVPP-- 4063
C.elegans           ---------PPPPKRGCFYIFVGLASMPSETVVTPHLATYFEQVLEPLPPSAVFQSQNN- 3145
                                 * . :  :: * *:*.* :::* :  ::*:.** :*

H.sapiens           SSQDEDMGHFEIPDPMEE-STTSLVSSSTSAYSSFPVDVVVYVRVQPSQIKFSCLP---- 4060
D.melanogaster      -----------TPSHNTG-VNLDILPANYVTYASFPVDVIVYFHMQPSTFRFSCLP---- 4107
C.elegans           ------TREASVPDDGKGDANNEVHNIMAMDTAAFPIDFVFYLDVQSSTIRFDGKQPTSR 3199
                                *.      . .:        ::**:*.:.*. :* * ::*.

H.sapiens           -VSRVECMLKLPSLDLVFSSNRGELETLGTTYPAETLSPGGNATQSGTKTSASKTGIPGS 4119
D.melanogaster      -VSRVECMLQLPSLDIVFSSKRSSEEENS-------AQPGGHTQ---------------- 4143
C.elegans           SQTQADCLLTLPRLTLELTSKRTRDNID-------------------------------- 3227
                      ::.:*:* ** * : ::*:*   :

H.sapiens           SGLGSPLGRSRHSSSQSDLTSSSSSSSSGLSFTACMSDFSLYVFHPYGAGKQKTAVSGLTP 4179
D.melanogaster      -------------------PDQQLPTGGLSVTGCLADFNVFIFHPYGGKKTSKE------ 4178
C.elegans           ----------------------NYVGGIHISGQFKGFMLKIYNPLELEPDS-------- 3256
                                          .*: .:. : .* : :::*      .

H.sapiens           GSGGLGNVDEEPTSVTGRKDSLSINLEFVKVSLSRIRRSGGASFFESQSVSKSASKMDTT 4239
D.melanogaster      -------TQFSPLSDSERKDSLSINVEFVKFHITRCRKV----YIEPLPSSKRSLDQSRA 4227
C.elegans           ------------------SRALQLSLDLLSFVISRNKNS---------------STEPDN 3283
                                      . :*.:.::::.. ::* :.

H.sapiens           LINISAVCDIGSASFKYDMRRLSEILAFPRAWYRRSIARRLFLGDQTINLPTSG---PG- 4295
D.melanogaster      VIRFSTIVDIGSASFKYDMRRLTEILAFPKAWYRRRIVRRLFLGDLSVQQQQQQQQGNGA 4287
C.elegans           RVRFVFSSQISKASFEYNFRRLGELIQFPKPWYRAAIARRVFFGDQAAPRQKDDASDITG 3343
                     :.:    :*..***:*::*** *:: **: ***  *.**:*:** :      .

H.sapiens           -TPDSIEGVSQHLSPESSRKAYCKTWEQPSQSASFTHMPQSPNVFNEHMTNSTMSPGTVG 4354
D.melanogaster      ETPTGCPPATPTPNEDASRAK-------DNMRLDFDGQPSQ----------QQQQLGHFG 4330
C.elegans           TTRS-------------------------------RLPTDPK------------------ 3354
                     *                                    * .

H.sapiens           QSLKSPASIRSRSVSDSSVPRRDSLSKTST-PFNKSNKAASQQGTPWETLVVFAINLKQL 4413
D.melanogaster      A-------VRH----------LKNLGKSSSAESSGTPPSEKNQITAWETLVIFAVNFTKL 4373
C.elegans           -SLQPP---------------------AASTASTGSGSFVPHQRKPWTALVLAAIQWNEF 3392
                                               :::   . :     :* . * :**: *:: .::

H.sapiens           NVQMNMSNVMGNTTWTTSGLKSQGRLSVGSNRDREISMSVGLGRSQLDSKGGVVGGTIDV 4473
D.melanogaster      NVQMNIGNVCGNVVWLTKDFQSDGRLSIGSTGYKNMYAGIYLGGSALDAKGGIVGGSFEV 4433
C.elegans           EVTAFMSNTMGKTTWKATKGLVWGDAKLNSLNERDVSISFVLGSSELCARDGAISGTIML 3452
                    :*   :.*. *:..* :.     *  .:.*   :::  .. ** * * ::.* :.*:: :

H.sapiens           NALEMVAHI--SEHPNQQPSHKIQITMGSTEARVDYMGSSILMGIFSNADLKLQDEWKVN 4531
D.melanogaster      NKINKRFHI--KEEAGMEPYHTMGLSFMALELRLDYMGTSVLMTRISSFSAAMKDEWRTA 4491
C.elegans           NNLKVSADHSLSADVKRVPVNKAKIRLEWITANIEWMSRRVLIAKWCGPSFKVNDYYKGL 3512
                    * ::   .   . .    * :.  : :     .:::*.  :*:   .. .  ::* ::

H.sapiens           LYNTLD---SSITDKSEIFVHGDLKWDIFQVMISRSTTPDLIKIGMKLQEFFTQQFDTSK 4588
D.melanogaster      SQAAATPAHGKDQPRALIFIHGDLTWDQLQIMISKSTTADLLKMYFKLEEFFTQQFKSSK 4551
C.elegans           KE-------GDHFALSELGMNVQASWKDLQVVITKSTVDDVAAIVNRLISFIDEQLKNSR 3565
                             ..    : : :: : .*. :*::*::**. *:  :  :* .*: :*:..*:

H.sapiens           RALSTWGPVPYLPPKTMTSN----------------LEKSSQEQLLDAAHHRHWPGVLKV 4632
```

```
D.melanogaster      RVFSNLEPRLQDRTASIKRRQQHKKKPANGELVAPPQIHGMIGENTDARHHRHWQKPLAQ 4611
C.elegans           ILLGNLSASTNLKKQAQ----------------------ALIE--SRKPTTHFWEKVLDY 3601
                      :..          :                       .           :.*   *

H.sapiens           VSGCHISL-FQIPLPEDGMQFGGSMSLHGNHMTLACFHGPNFRSKSWALFHLEEPNIAFW 4691
D.melanogaster      AVGLVVPS-LVTRLPRHGNVLGGTVELRGQNISLACFHGINFKSKSWALFSLRSPSINFA 4670
C.elegans           MSEMQMNEQLMGLMEREGAKVGGHIELKAGGISLVMMKG-DMNADTWAVFHLRDACILFD 3660
                         :   :   : ..*  .** :.*:.  ::*. ::* ::.:.:**:* *..  * *

H.sapiens           TEAQKIWEDGSSDHSTY--IVQTLDFHLGHNTMVTKPCGALESPMATITKITRRRHENPP 4749
D.melanogaster      TEARQSE------DEVL--VTQTLTSSLGQTTEVQQQQ---NHSMAIVSRITRN--IIFP 4717
C.elegans           PEARMDFLDNSSQQKIGILLKQTFCLQLGSRHGNQTENR--ANVCRVQTRFNNSRHLQK- 3717
                     **:         ..    : **:   **                   :::..

H.sapiens           HGVASVKEWFNYVTA----TRNEEL-------NLLRN-VDAN-NTEN-----STTVKNSS 4791
D.melanogaster      PQFKTLNEWFHYAFA----NSEIDAVDRFPMLECERE-IASN-SIER-----T----RAS 4762
C.elegans           --AEDILEFFIGDVMKIIGSADHSEKKKLKEVEVIQSPISENENTAKSPTSTFSRFRSPG 3775
                         : *:*         . : .        :  :. :  * .  .            .

H.sapiens           LLSGFRGGSSYNHETETIFALPRMQLDFKSIHVQEPQEPSLQDAS--LKPKVECSVVTEF 4849
D.melanogaster      GSSSAAKAQEHNHNREVIFALPSLQLHFKTEHKQGPTTPEP--SE--NKPEVLCSFITEF 4818
C.elegans           TSKTKESGPATNHNVMELFQFPGLEAKMSSQQLNGVDDGDKYESVFQMPMDVLTTFVCDF 3835
                      .    .   **:   :* :* :: .:.: : :     .   :      .*  :.: :*

H.sapiens           TDHICVTM-DAELIMFLHDLVSAYLKEKEKAIFPPRILSTRP------------------ 4890
D.melanogaster      DDHIFVTV-DADAFFFLHDLITSYVTEKEKVIGAQSARAASPNLSQKANLKPYLTDEILK 4877
C.elegans           FSEVAIETNFNAQVSFLPELLKSYLKESHSGTSSSHS----------------------- 3872
                     ..: :       . ** :*:.:*:.*...

H.sapiens           ---GQKSPIII------------------------------------------------- 4898
D.melanogaster      EKKGASNTNLTAQGKQTSGSKNSLDPLQGSHTSLANAAANMSGAATTNTTTTTATTLSP 4937
C.elegans           ------------------------------------------------------------ 3872

H.sapiens           ----------HDDNSSD-K-------DREDSITYTTVDWRDFMCNTWHLEPTLRLISWTG 4940
D.melanogaster      GAAAGGPSTSATNDSVDGKQQQQEGSPPTFDLESFVRDWRHFECQTWHLEPTVRLLSWAG 4997
C.elegans           ------------TNSSP-----AVSSSKESVVSETSKDPRIFTCQEWKVEPRVRFIDRI- 3914
                                 :*                 :     * * * *: *::** :*::.

H.sapiens           RKIDPVGVDYILQKLGFHHARTTIPKWLQRGVMDPLDKVLSVLIKKLGTALQDEKEKK-- 4998
D.melanogaster      KSIEPYGVDYILNKLGFSHARTTIPKWLQRGFMDPLDKVQALMMLQLLLMVRENKVERDS 5057
C.elegans           -KWTPPVLDDILKKLQIFDHRNTIPKVIQRAVLDPLDATLAASVIATLQIVDNKKTIQKF 3973
                     .  *  :* **:** : . *.**** :**..:**** . :  :      : ::*  :

H.sapiens           ---GKDKEEH------------------------------------- 5005
D.melanogaster      GASGSGKQQQ----QNHRPPTN------------------------- 5075
C.elegans           KKSRTDSMAPTPKRRDSRRSSEEVSVSIDIPDIITDISDASFRPKHN 4020
                        ...
```

B

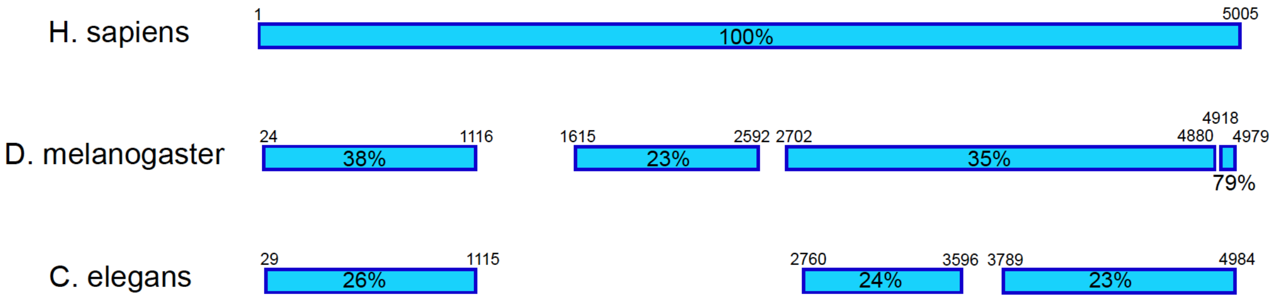
H. sapiens
1
5005
100%
D. melanogaster
24
1116
38%
1615
2592
23%
2702
4880
35%
4918
4979
79%
C. elegans
29
1115
26%
2760
3596
24%
3789
4984
23%

Figure S8: KIAA1109 protein conservation

(A) Multi-alignments of *Homo sapiens* KIAA1109, *Caenorhabditis elegans* lpd-3 and *Drosophila melanogaster* tweek proteins obtained using Clustal Omega (v1.2.3).

(B) Schematic representation of aligned segments of KIAA1109 proteins obtained using BLASTP tool on Ensembl. *Homo sapiens* protein sequence was used as query and *D. melanogaster* and *C. elegans* as subjects. Percentage of identity between the *H. sapiens* sequence and the subjects are indicated. The numbering pinpoints the coordinate of the human residues at the beginning and end of human regions with similarities. *H. sapiens* NM_015312, *D. melanogaster* NM_ 001201898 and *C. elegans* NM_001313537.

Table S1: Predicted pathogenicity and allele frequencies of the variants identified in *KIAA1109* gene

Family	Inheritance	Mutation coordinates (GRCh37/hg19)	Amino Acid change	dbSNP v147	Allele frequency (ExAC v0.3.1)	PolyPhen2 prediction (score)	PROVEAN prediction (score)	SIFT prediction (score)
LT	Compound heterozygous	Chr4:123160823; c.3986A>C	Tyr1329Cys	rs770791100	0.000041	Probably damaging (0.993)	Deleterious (-2.52)	Damaging (0.001)
		Chr4:123170727; c.5599G>A	Val1867Met	-	-	Probably damaging (0.969)	Neutral (-0.45)	Damaging (0.024)
UK	Compound heterozygous	Chr4:123164200; c.4719G>A	Met1573Ile	rs368227278	0.000008	Benign (0.000)	Neutral (-0.64)	Tolerated (0.835)
		Chr4:123171679; c.5873G>A	Arg1958Gln	-	-	Benign (0.001)	Neutral (-0.44)	Tolerated (0.594)
AL	Homozygous recessive	Chr4:123207807; c.9149C>A	Pro3050His	-	-	Probably damaging (1.000)	Deleterious (-7.36)	Damaging (0.000)
TU1	Homozygous recessive	Chr4:123230520; c.10153G>C	Gly3385Arg	-	-	Probably damaging (1.000)	Deleterious (-6.17)	Damaging (0.001)
TU2	Homozygous recessive	Chr4:123230520; c.10153G>C	Gly3385Arg	-	-	Probably damaging (1.000)	Deleterious (-6.17)	Damaging (0.001)
SA1	Homozygous recessive	Chr4:123128323; c.1557T>A	Tyr519Ter	rs730882245	-	-	-	-
SA2	Homozygous recessive	Chr4:123252480; c.11250-1G>A	His3751_Arg3822del	-	-	-	-	-
SA3	Homozygous recessive	Chr4:123258092; c.12067G>T	Glu4023Ter	-	-	-	-	-
US	Compound heterozygous	Chr4:123254885_123263438delinsG; c.11567_12352delinsG	Lys3856Argfs*44	-	-	-	-	-
		Chr4:123113479; c.997dupA	Ile333Asnfs*5	-	-	-	-	-
SG	Compound heterozygous	Chr4:123147970; c.2902C>T	Arg968Cys	-	-	Probably damaging (0.966)	Deleterious (-2.53)	Tolerated (0.058)
		Chr4:123159280; c.3611delA	Asn1204Thrfs*6	-	0.000008309	-	-	
